# Circulating tumour DNA-Based molecular residual disease detection in resectable cancers: a systematic review and meta-analysis

**DOI:** 10.1016/j.ebiom.2024.105109

**Published:** 2024-04-13

**Authors:** Jiachun Zheng, Chuling Qin, Qianxi Wang, Dongbo Tian, Zisheng Chen

**Affiliations:** a Department of Respiratory and Critical Care Medicine, Affiliated Qingyuan Hospital of Guangzhou Medical University, Qingyuan People's Hospital, Qingyuan, 511518, China; b Guangzhou Medical University, Guangzhou, 511436, China

**Keywords:** Molecular residual disease (MRD), Circulating tumour DNA, Surgery, Adjuvant therapy, Meta-analysis

## Abstract

**Background:**

Circulating tumour DNA (ctDNA)-based molecular residual disease (MRD) detection technology has been widely used for recurrence evaluation, but there is no agreement on the efficacy of assessing recurrence and overall survival (OS) prognosis, as well as the sensitivity and specificity of landmark detection and longitudinal detection.

**Methods:**

We systematically searched Pubmed, Embase, Cochrane, and Scopus for prospective studies or randomized controlled trials that collected blood samples prospectively. The search period was from Jan 1, 2013, to Sept 10, 2023. We excluded retrospective studies. The primary endpoint was to assess the hazard ratio (HR) between circulating tumour DNA positive (ctDNA+) and negative (ctDNA-) for recurrence-free survival incidence (RFS), disease-free survival (DFS), progression-free survival (PFS), event-free survival (EFS), time to recurrence (TTR), distant metastasis-free survival (DMFS) or OS in patients with resectable cancers. We calculated the pooled HR of recurrence and OS and 95% confidence interval (CI) in patients with resected cancers using a random-effects model. Pooled sensitivity and specificity were estimated using the bivariate random effects model.

**Findings:**

This systematic review and meta-analysis returned 7578 records, yielding 80 included studies after exclusion. We found that the HR of recurrence across all included cancers between patients with ctDNA+ and ctDNA- was 7.48 (95% CI 6.39–8.77), and the OS was 5.58 (95% CI 4.17–7.48). We also found that the sensitivity, area under the summary receiver operating characteristic curve (AUSROC) and diagnostic odds ratio (DOR) of longitudinal tests were higher than that of landmark tests between patients with ctDNA+ and ctDNA- (0.74, 95% CI 0.68–0.80 vs 0.50, 95% CI 0.46–0.55; 0.88 vs. 0.80; 25.70, 95% CI 13.20–45.40 vs. 9.90, 95% CI 7.77–12.40).

**Interpretation:**

Postoperative ctDNA testing was a significant prognosis factor for recurrence and OS in patients with resectable cancers. However, the overall sensitivity of ctDNA-MRD detection could be better. Longitudinal monitoring can improve the sensitivity, AUSROC, and DOR.

**Funding:**

Special fund project for clinical research of Qingyuan People's Hospital (QYRYCRC2023006), plan on enhancing scientific research in GMU (GZMU-SH-301).

**Research in context**

**Evidence before this study**

More and more studies have demonstrated that circulating tumour DNA (ctDNA)-MRD (minimal residual disease) analysis can detect recurrence earlier than imaging, and we hypothesized that patients with ctDNA+ are at greater risk of recurrence than patients with ctDNA-. We systematically searched Pubmed, Embase, Cochrane and Scopus, supplemented by ASCO, ESMO, and Google, for prospective studies or randomized controlled trials that collected blood samples prospectively. The search period was from Jan 1, 2013, to Sept 10, 2023. Keywords used include “Molecular Residual Disease (MRD),” “circulating tumour DNA,” “surgery,” “Drug Therapy, Adjuvant,” “Chemotherapy,” “Adjuvants, Immunologic,” “Radiotherapy, Adjuvant,” and “Targeted therapy” and their MeSH terms. We excluded retrospective studies and considered only papers published in English.

**Added value of this study**

This meta-analysis provided more comprehensive and standardized evidence of the hazard ratio (HR) of recurrence and OS, and summarized the primary data of patients with different cancers. It indicated that ctDNA was a risk factor for recurrence in resectable cancers, and the sensitivity of longitudinal detection was higher than landmark detection. In addition, the subgroup analysis of detection time and technology determined which time point and which detection technology was the most appropriate for different cancers. At the same time, the benefit of postoperative adjuvant therapy for patients with ctDNA+ in CRC may be a hint that ctDNA detection can be used as clinical evidence of ctDNA-based adjuvant therapy.

**Implications of all the available evidence**

Our findings had several potential implications for clinical practice and future research. First of all, our result showed that ctDNA was a prognostic factor in resectable cancers, and it can be considered an indicator of postoperative recurrence detection, but the detection time and detection technology should be considered simultaneously. Secondly, patients with ctDNA+ in post-adjuvant therapy also had a recurrence risk, and this indicates that further study of the value of ctDNA+ should focus on analyzing the difference and relationship between the ctDNA-based adjuvant therapy group and the conventional management therapy group. What's more, longitudinal detection showed a high sensitivity for detecting recurrence, which made it interesting to think about when to test.

## Introduction

Cancer remains a major public health problem worldwide. According to Cancer Statistics, 2023,^1^ until 2020, in America, colorectal cancer (CRC), lung cancer, pancreatic adenocarcinoma (PAAD), bladder cancer (BLCA), melanoma, breast cancer (BC), gastric cancer (GC), hepatocellular carcinoma (HCC), ovarian cancer (OV) and esophageal carcinoma (ESCA) predicted for 7.81%, 12.17%, 3.27%, 4.20%, 4.98%, 15.35%, 1.35%, 2.10%, 1.01%, 1.10% of new cases in 2023, and the mortality rates were 8.62%, 20.84%, 8.29%, 2.74%, 1.31%, 7.17%, 1.83%, 4.82%, 2.18%, 2.64%, respectively. In China, the morbidity rates in the above cancers at last count were 10.04%, 20.38%, 2.47%, 2.03%, 0.17%, 7.53%, 9.76%, 9.57%, 1.41%, 6.21%, and the mortality rates were 8.10%, 27.22%, 3.64%, 1.40%, 0.16%, 2.97%, 11.95%, 13.94%, 1.13%, 8.03%.^2^

With the large-scale development of low-dose chest computed tomography (LDCT), gastrointestinal endoscope, mammography screening^3^ and other measures, early cancers were more likely to be detected. An increase in treatment methods, such as neoadjuvant single/double immunotherapy, targeting, chemoradiotherapy plus immunotherapy, and combined surgical resection, has potentially cured more people. However, there is still a proportion of patients with relapse after surgery, and traditional methods of monitoring for recurrence post-operation, including imaging, may have difficulty detecting microscopic recurrence.

Circulating tumour DNA (ctDNA) is cancer-related free DNA fragments released into the blood by apoptotic or necrotic tumour cells, which do not exist in normal people. With the development of cancer monitoring technology, ctDNA-MRD was superior to imaging and could detect recurrence 5.01 months before imaging,^4^ indicating the existence of micro-residual and assessing the overall survival (OS). Furthermore, adjuvant therapy based on ctDNA positive (ctDNA+) seems to be beneficial, but ctDNA negative (ctDNA-) adjuvant therapy has not shown benefits.^5^ Based on this, the current National Comprehensive Cancer Network (NCCN) treatment guidelines^6^ and Chinese expert consensus^7^ recommend ctDNA-MRD for CRC and NSCLC detection. However, methods and strategies for detecting ctDNA-MRD in patients vary from study to study and from cancer to cancer, which presents a challenge for clinicians to interpret MRD results. For example, the current detection methods, including Droplet Digital PCR (ddPCR),^8^ multiplex PCR next generation sequencing (mPCR-NGS),^9^ whole genome sequencing (WGS),^10^ Fast Aneuploidy Screening Test-sequencing System (FAST-SeqS),^11^ hybridization capture-based NGS,^12^ circulating single-molecule amplification and resequencing technology (cSMART),^13^ Guardant Reveal,^14^ ctDNA methylation assays,^15^ single-cell universal poly (A)-independent RNA sequencing (SUPeR-seq),^16^ which makes result diversity.

To investigate the role of ctDNA-MRD in survival benefits and landmark/longitudinal detection for resectable cancers, we conducted a systematic meta-analysis to analyze survival benefits between patients with ctDNA+ and ctDNA- and determine the overall sensitivity and specificity of ctDNA-MRD as a prognostic biomarker. Subgroup analyses were conducted based on ctDNA testing time and technique and whether adjuvant therapy was performed.

## Methods

### Search strategy and selection criteria

We systematically searched Pubmed, Embase, Cochrane, and Scopus, supplemented by ASCO, ESMO, and Google, for prospective studies or randomized controlled trials that collected blood samples prospectively. The search period was from Jan 1, 2013, to Sept 10, 2023. Keywords used include “Molecular Residual Disease (MRD),” “circulating tumour DNA,” “surgery,” “Drug Therapy, Adjuvant,” “Chemotherapy,” “Adjuvants, Immunologic,” “Radiotherapy, Adjuvant,” and “Targeted therapy” and their MeSH terms.

The inclusion criteria: (1) prospective studies or randomized controlled trials which collected blood samples prospectively; (2) patients with confirmed resectable cancers (I-IV stage); (3) The analysis group was classified as patients with ctDNA+ and ctDNA-; (4) data available on outcome indicators: disease-free survival (DFS), progression-free survival (PFS), relapse-free survival (RFS), event-free survival (EFS), time to recurrence (TTR), distant metastasis-free survival (DMFS) or OS was reported as HR; (5) ctDNA testing in postoperative or post-adjuvant therapy after surgery; (6) ctDNA-MRD landmark detection or longitudinal detection in postoperative; (7) only papers published in English. The exclusion criteria: (1) unresectable cancer; (2) Individual cases, non-availability of data (HR of DFS, PFS, RFS, EFS, TTR, DMFS and OS or the number of patients with ctDNA+ and ctDNA-); (3) No pre-designed blood samples were collected or it is not clear if there was a pre-design blood collection; (4) ctDNA present in urine or other body fluids; (5) The time of ctDNA blood collection and analysis was non-postoperation; (6) articles with inconsistent titles and abstracts, reviews, animal tests, and systematic reviews. Three researchers (J.Z., C.Q., Q.W.) screened each record, and each report was retrieved independently, and a consensus was reached between 3 reviewers in disputes between them.

### Study quality

Three researchers (J.Z., C.Q., Q.W.) evaluate the quality of the article, and if there is a dispute, all researchers discuss and reach a consensus decision. All studies were analyzed using the Newcastle-Ottawa Scale (NOS)^17^ as a measure of bias. All studies with a performance score of at least 7 were considered high-quality studies.

### Data collection

Data was extracted independently by 3 investigators (J.Z., C.Q., Q.W.), and a consensus was reached between 3 reviewers in disputes between them. The senior investigators reviewed the results (D.T., Z.C.). Information regarding target outcomes was obtained and contained in Microsoft Office software when available. General data was recorded from each study: the first author, year of publication, journal, the age of the subjects, sex, intervention, the population's country, sample extraction time, the number of patients with ctDNA+ and ctDNA-, the number of patients with recurrence and non-recurrence, tumour type and stage, adjuvant treatment, ctDNA detection method, follow-up time and measurement indicators of the study subjects, etc. The HR of RFS, DFS, PFS, EFS, TTR and DMFS for detecting recurrence and OS was obtained to compare patients with ctDNA+ and ctDNA- groups.

Furthermore, we compared the sensitivity and specificity of each cancer landmark and longitudinal detection analysis. Landmark detection is defined as a single test at a pre-specified time point after surgery, and patients with positive detection are classified as ctDNA+. Longitudinal detection is defined as multiple detections within the follow-up time after surgery, and patients with positive detection are also classified as ctDNA+. At the same time, we also included the sensitivity and specificity of ctDNA detection technology.

### Data analysis

Based on each study, we estimated or calculated the HR for each risk factor and outcomes of interest with 95% CI. RFS, DFS, PFS, EFS, TTR and DMFS were measured from the date of surgery to the verified first radiologic recurrence (local or distant) or death as a result of cancer/any cause while OS was detecting death.^13^^,^^18^, ^19^, ^20^ Pooled HR of RFS, DFS, PFS, EFS, TTR and DMFS for detecting recurrence and OS and their 95% CI were generated using a random-effects model due to the prevalence of heterogeneity. Pooled sensitivity and specificity and diagnostic odds ratio (DOR) were estimated using the bivariate random effects model.^21^ The diagnostic performance of each subgroup was compared by drawing sROC curves using a bivariate random effects model.^21^ The AUSROC was estimated using a univariate model^22^ and the bivariate random effects model.^21^ To test for heterogeneity between studies, we use Q-test,^23^ Higgins,^24^ Chi-square,^25^ and Zhou and Dendukuri's approach.^26^ The heterogeneity was defined as I^2^>50%,^27^ and the P-value was testing performance. Furthermore, subgroup analyses were conducted based on ctDNA testing time, technique and whether adjuvant therapy was performed. The reporting bias was assessed using a funnel plot and Egger's and Begg's tests. We used the Trim-and-fill method to estimate and adjust the number and outcomes of missing studies when the reporting bias existed. We performed a regression analysis to monitor time and technique, the quality of included studies, and the proportion of patients with ctDNA- and ctDNA+. We performed a sensitivity analysis by removing each study in turn. The meta (version 6.5-0)^28^ and mada (version 0.5.11)^25^ packages in R (version 4.2.3) were used to conduct all statistical analyses. The forestploter (version 3.1.3)^29^ package in R was used to conduct to form forest map of the data generated by meta package. The adjuvant treatment group was defined as patients all receiving adjuvant treatment, and the non-adjuvant treatment group was defined as no patient in the group receiving adjuvant treatment. Pan cancer was defined as those cancers we included.

In all cases, P-values were 2-tailed; statistical significance was defined as P-value <0.05. It was registered on Prospero before starting the searches (registration number CRD42023438133); an updated search was done on Sept 10, 2023. And until Oct 10, we systematically searched for updates that were relevant to the included abstracts. All information sources were taken from the included studies and their Supplementary Materials.

### Role of the funding source

The funder of the study had no role in study design, data collection, data analysis, data interpretation, or writing of the report.

### Ethics statement

No ethical approvals were required for this study.

## Results

### Search results

**Fig. 1:**
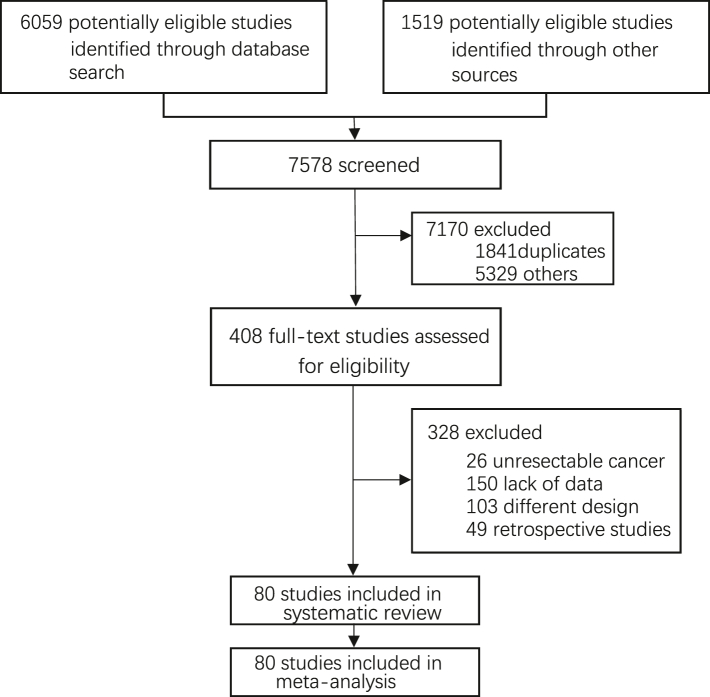
Study selection PRISMA (preferred reporting items for systematic reviews and meta-analyses) 2020 flow diagram for systematic review of HRs of recurrence and survival for resectable cancer in CRC, NSCLC, CRLM, PAAD, BLCA, melanoma, BC, GC, HCC, OV and ESCA.

A total of 7578 articles were retrieved, including 1363 from Pubmed, 3416 from Embase, 454 from Cochrane, 826 from Scopus and 1519 from other databases. One thousand eight hundred forty-one duplicated articles were screened, and 5329 articles with inconsistent titles and abstracts, reviews, animal tests, and systematic reviews were excluded. Three hundred and twenty-eight articles did not meet the inclusion criteria, and as a result, 80 articles were included. Until Oct 10, we have updated one study relevant to the included abstract. In the end, we included 18 abstracts^30^, ^31^, ^32^, ^33^, ^34^, ^35^, ^36^, ^37^, ^38^, ^39^, ^40^, ^41^, ^42^, ^43^, ^44^, ^45^, ^46^, ^47^ and 62 studies,^4^^,^^13^^,^^15^^,^^20^^,^^48^, ^49^, ^50^, ^51^, ^52^, ^53^, ^54^, ^55^, ^56^, ^57^, ^58^, ^59^, ^60^, ^61^, ^62^, ^63^, ^64^, ^65^, ^66^, ^67^, ^68^, ^69^, ^70^, ^71^, ^72^, ^73^, ^74^, ^75^, ^76^, ^77^, ^78^, ^79^, ^80^, ^81^, ^82^, ^83^, ^84^, ^85^, ^86^, ^87^, ^88^, ^89^, ^90^, ^91^, ^92^, ^93^, ^94^, ^95^, ^96^, ^97^, ^98^, ^99^, ^100^, ^101^, ^102^, ^103^, ^104^ 73 of which were prospective studies, and 7 were randomized controlled trials (Table 1). The flow chart is as follows: Fig. 1.

**Table 1:** Characteristics of the eligible trials.

**Report type**	
Clinical trial	62 (78%)
Conference abstract	18 (22%)
**Trial design**	
Prospective studies	73 (91%)
Randomized controlled trial	7 (9%)
**Participant**	
Mid-age	
∼50	3 (4%)
51∼60	18 (22%)
61∼70	33 (41%)
71∼	4 (5%)
Unknown	22 (28%)
**ctDNA detection technology**	
Hybridization capture-based NGS	34 (42%)
mPCR-NGS	25 (31%)
ddPCR	13 (16%)
Guardant Reveal	4 (5%)
cSMART	2 (3%)
ctDNA methylation	2 (3%)
**Cancer**	
CRC	20 (25%)
NSCLC	14 (17%)
CRLM	10 (12%)
PAAD	7 (9%)
ESCA	6 (8%)
BC	6 (8%)
GC	5 (6%)
BLCA	5 (6%)
OV	3 (4%)
Melanoma	3 (4%)
HCC	1 (1%)
**Mid-follow up(month)**	
1∼10	6 (8%)
11∼20	21 (26%)
21∼30	18 (22%)
31∼40	15 (19%)
>40	7 (9%)
Unknown	13 (16%)
**Outcome**	
Only recurrence	54 (67%)
OS and recurrence	24 (30%)
Only OS	2 (3%)
**Detection time**	
Landmark	52 (65%)
Landmark and Longitudinal	10 (12%)
Landmark and Adjuvant therapy	5 (6%)
Landmark, Longitudinal and Adjuvant therapy	7 (9%)
Longitudinal	6 (8%)

Twenty studies were for CRC, 14 for non-small lung cancer (NSCLC), 10 for colorectal liver metastases (CRLM), 7 for PAAD, 6 for ESCA, 6 for BC, 5 for GC, 5 for BLCA, 3 for OV, 3 for melanoma and 1 for HCC (Table 1).

The quality evaluation table of all studies was in Table S1.

### Description of eligible studies

This study included 54 studies with only recurrence, 24 with recurrence and OS, and 2 with only OS. From the perspective of various national studies, China had the most studies, which may be related to the incidence of NSCLC (Table S2). The data and features of the included articles are shown in Table 1 and Tables S2–S6.

### Recurrence and OS detection and analysis

**Fig. 2:**
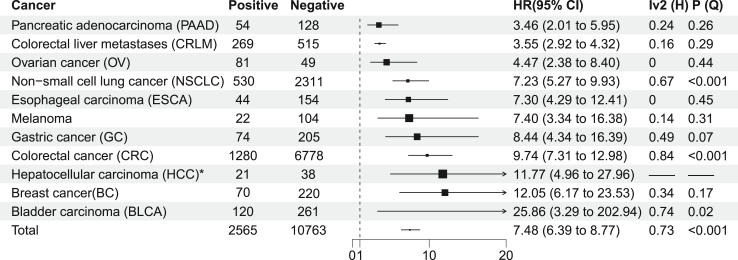
Summary collection of HRs of univariate analysis of recurrence of CRC, NSCLC, CRLM, PAAD, BLCA, melanoma, BC, GC, HCC, OV and ESCA; ∗ = single study; Negative = ctDNA-; Positive = ctDNA+. Vertical dashed line is invalid line, and 95% confidence interval crossing is not statistically significant; H = Higgins' approach; Q = Q-test.

**Fig. 3:**
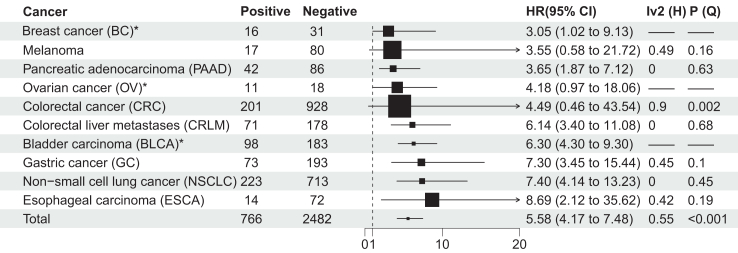
Summary collection of HRs of univariate analysis of OS of CRC, NSCLC, CRLM, PAAD, BLCA, melanoma, BC, GC, OV and ESCA; ∗ = single study; Negative = ctDNA-; Positive = ctDNA+; Vertical dashed line is invalid line, and 95% confidence interval crossing is not statistically significant; H = Higgins' approach; Q = Q-test.

For each cancer, pooled HR of recurrence and OS were shown in Figure S1 and Figure S2. Two thousand five hundred sixty-five patients with ctDNA+ and 10,763 patients with ctDNA- were in recurrence detection, 766 patients with ctDNA+ and 2482 patients with ctDNA- were in OS detection (Figs. 2 and 3). We presented a random effect model-based pan-cancer meta-analysis. By univariate analysis, excluding only single studies (HCC), the highest pooled HR of recurrence was BLCA (25.86, 95% CI 3.29–202.94, p (Q-test) = 0.02, I^2^ (Higgins) = 74%), and the lowest pooled HR was PAAD (3.46, 95% CI 2.01–5.95, p (Q-test) = 0.26, I^2^ (Higgins) = 24%). Excluding only single studies (BC, OV and BLCA), the highest pooled HR of OS was ESCA (8.69, 95% CI 2.12–35.62, p (Q-test) = 0.19, I^2^ (Higgins) = 42%), and the lowest pooled HR was melanoma (3.55, 95% CI 0.58–21.72, p (Q-test) = 0.16, I^2^ (Higgins) = 49%). Summarizing the HR of recurrence and OS in pan-cancer, the pooled HR of recurrence was 7.48 (95% CI 6.39–8.77, p (Q-test) <0.001; I^2^ (Higgins) = 73%) and the OS was 5.58 (95% CI 4.17–7.48, p (Q-test) <0.001; I^2^ (Higgins) = 55%), respectively. Overall, there was no significant heterogeneity among cancers except for NSCLC, CRC, and BLCA for recurrence detection. For OS, there was no significant heterogeneity among cancers except for CRC (Figs. 2 and 3). The multivariable analysis also showed the ctDNA was a prognosis factor for recurrence and OS (pooled HR of recurrence: 7.07 (95% CI 5.66–8.83, p (Q-test)<0.001, I^2^ (Higgins) = 79%); pooled HR of OS: 2.76 (95% CI 1.85–4.12, p (Q-test) = 0.06, I^2^ (Higgins) = 43%) in all cancers (Figures S3 and S4). Most of the studies were adjusted variables for age, sex, and pathological stage (Table S7).

We conducted a subgroup analysis to observe whether there was any difference in HR of recurrence with different detection times. The result showed that the recurrence survival benefit of longitudinal detection was higher than that at the landmark detection in patients with ctDNA- in NSCLC, CRC and BC except ESCA (Figures S5–S8). Although CRLM, GC, and BLCA were the only single studies in longitudinal detection, we can still observe the same phenomenon as many longitudinal combined studies (Figures S9–S11). Patients with ctDNA- in post-adjuvant therapy also have recurrence survival benefits. We also summarized these studies in Figures S12 and S13.

Furthermore, subgroup analysis was also performed in recurrence according to with or without adjuvant therapy in landmark detection in CRC. It showed a significantly reduced risk of recurrence after adjuvant therapy in landmark detection for patients with ctDNA+ compared to those with no treatment (6.63, 95% CI 4.24–10.35, p (Q-test) = 0.57, I^2^ (Higgins) = 0% vs 17.67, 95% CI 9.47–32.96, p (Q-test) = 0.93, I^2^ (Higgins) = 0%, Figure S14).

### Sensitivity and specificity

#### Pool analysis

We extracted data with calculable sensitivity and specificity from the included studies. For each cancer, pooled sensitivity and specificity were shown in Table 2. Summarizing the sensitivity and specificity of pan-cancer, we found that pooled sensitivity and specificity of cancer recurrence were 0.58 (95% CI 0.53–0.62, I^2^ (Zhou and Dendukuri's approach) = 2.40%) and 0.90 (95% CI 0.88–0.92, I^2^ (Zhou and Dendukuri's approach) = 2.40%), respectively.

**Table 2:** The Sens, Fpr, AUSROC (AUC) and DOR of each cancer by bivariate random effects model.

Subgroups	Number of data	Sens	LL	UL	Fpr	LL	UL	Iv2 (Z and D)	DOR	LL	UL	AUC
Cancer												
CRLM	9	0.49	0.40	0.57	0.11	0.07	0.17	11.30%	7.86	4.31	13.20	0.85
1	8	0.45	0.39	0.52	0.11	0.07	0.17	0%	6.70	3.91	10.70	0.71
2	1	–	–	–	–	–	–	–	–	–	–	–
ESCA	5	0.50	0.35	0.66	0.09	0.04	0.18	0%	11.80	3.95	27.70	0.88
1	3	–	–	–	–	–	–	–	–	–	–	–
2	2	–	–	–	–	–	–	–	–	–	–	–
GC	6	0.55	0.38	0.72	0.14	0.09	0.21	0%	8.31	2.91	18.90	0.85
1	5	0.45	0.34	0.57	0.16	0.11	0.23	5.70%	4.69	2.27	8.63	0.77
2	1	–	–	–	–	–	–	–	–	–	–	–
CRC	18	0.58	0.48	0.68	0.08	0.06	0.11	12.90%	16.10	9.14	26.20	0.89
1	14	0.51	0.41	0.61	0.09	0.07	0.11	9.90%	11.40	6.99	17.50	0.89
2	4	0.84	0.73	0.91	0.05	0.01	0.20	0%	154.00	14.80	638.00	0.89
PAAD	4	0.58	0.44	0.71	0.13	0.04	0.32	0%	11.90	2.87	33.40	0.66
1	4	0.58	0.44	0.71	0.13	0.04	0.32	0%	11.90	2.87	33.40	0.66
NSCLC	15	0.61	0.51	0.70	0.11	0.07	0.16	15.90%	13.60	7.73	22.20	0.83
1	7	0.45	0.35	0.55	0.07	0.03	0.13	0%	11.80	6.41	20.10	0.71
2	8	0.75	0.65	0.82	0.14	0.09	0.22	0%	19.50	7.14	43.20	0.86
BC	5	0.65	0.42	0.82	0.04	0.02	0.09	0%	58.40	11.40	182.00	0.96
1	3	–	–	–	–	–	–	–	–	–	–	–
2	2	–	–	–	–	–	–	–	–	–	–	–
HCC	1	–	–	–	–	–	–	–	–	–	–	–
BLCA	4	0.59	0.51	0.66	0.11	0.08	0.16	1.30%	11.40	6.58	18.50	0.75
1	3	–	–	–	–	–	–	–	–	–	–	–
2	1	–	–	–	–	–	–	–	–	–	–	–
OV	2	–	–	–	–	–	–	–	–	–	–	–
1	2	–	–	–	–	–	–	–	–	–	–	–
melanoma	1	–	–	–	–	–	–	–	–	–	–	–
Time	70	0.58	0.53	0.62	0.10	0.08	0.12	2.40%	12.90	9.94	16.50	0.85
1	50	0.50	0.46	0.55	0.09	0.08	0.11	1.70%	9.90	7.77	12.40	0.80
2	20	0.74	0.68	0.80	0.11	0.07	0.15	6.90%	25.70	13.20	45.40	0.88

The Iv2 estimated by Zhou and Dendukuri approach (Z and D); 1 = landmark detection; 2 = longitudinal detection; Sens: sensitivity; Fpr: false-positive rate.

DOR: diagnostic odds ratio; AUSROC (AUC): area under the summary receiver operating characteristic curve.

The meta-regression analysis showed that the heterogeneity of sensitivity was due to the monitoring time (p (z-test) <0.001) and the proportion of patients with positive (p (z-test) <0.001) in the landmark. Detection technology (A = mPCR-NGS, B = ddPCR, C = hybridization capture-based NGS) was a significant difference in landmark (p (z-test) = 0.021, Table S8).

#### Subgroup analysis

Next, we performed a subgroup analysis of the detection time. One thousand five-hundred four patients with relapse were 770 ctDNA+, and 3629 patients without relapse were 288 ctDNA+ at the landmark time. Four hundred ninety-eight patients with relapse were 373 ctDNA+, and 1315 patients without relapse were 139 ctDNA+ at the longitudinal time. Due to insufficient cancer studies, only CRC, NSCLC, CRLM, ESCA, PAAD, BC, BLCA and GC were analyzed via a bivariate model (Table 2). All cancer sensitivity was greater than 0.5 except CRLM and ESCA. In a landmark, the lowest sensitivity of the merge was CRLM, NSCLC and GC, and the highest was PAAD (Table 2).

**Fig. 4:**
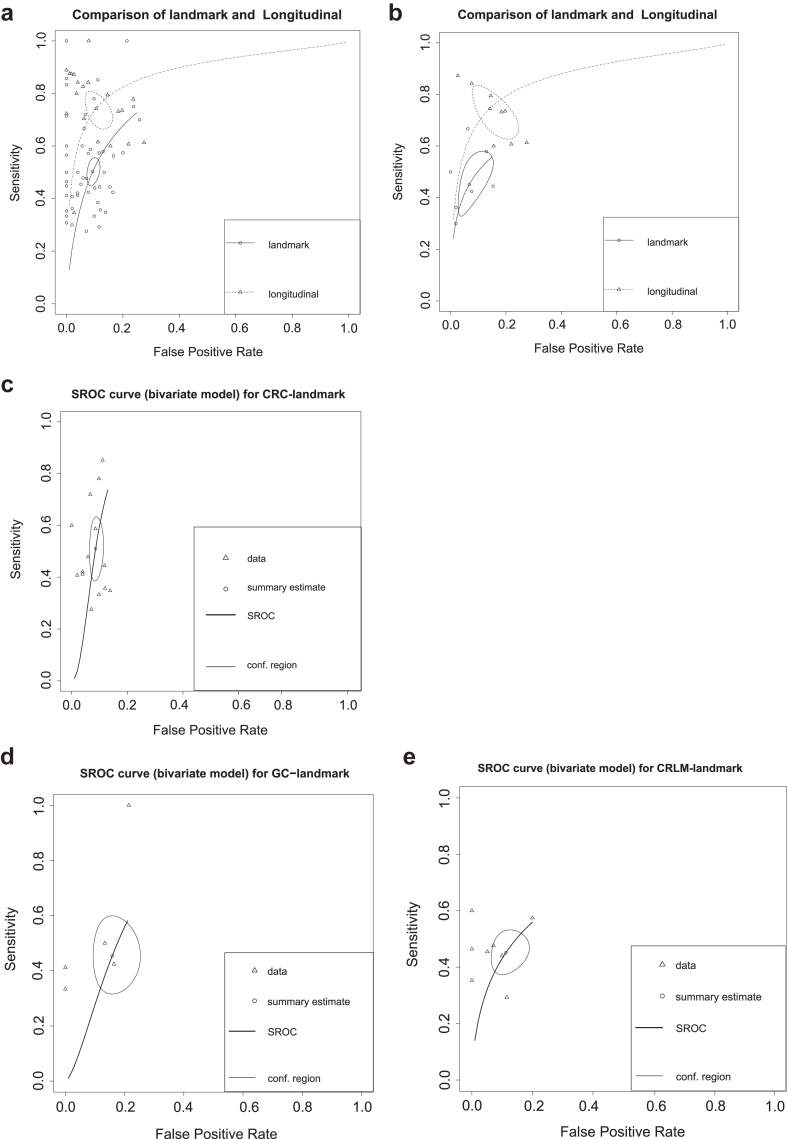
SROC curve (bivariate model) of each cancer: a: sROC curve for all cancers combinations of landmark detection and longitudinal detection; b: sROC curve for NSCLC combinations of landmark detection and longitudinal detection; c: sROC curve for CRC combinations of landmark detection; d: sROC curve for GC combinations of landmark detection; e: sROC curve for CRLM combinations of landmark detection. conf. region: estimate of sensitivity and specificity to show the region containing likely combinations of the mean value of sensitivity and specificity.

CRLM detected the AUSROC of landmark detection with the lowest value and CRC with the highest value by univariate analysis, while NSCLC and CRLM had the lowest value and CRC with the highest value by bivariate analysis (Table 3). GC detected the DOR of landmark detection with the lowest value and with the PAAD highest value (Table 2). AUSROC is a comprehensive indicator that is more reliable than a single indicator such as DOR, so AUSROC results were adopted. In SROC meta-regression models, longitudinal detection was the better method to detect recurrence than landmark detection with AUSROC of 0.88 vs 0.80 and with DOR of 25.7 (95% CI 13.20–45.40) vs 9.90 (95% CI 7.77–12.40) (Table 2) by bivariate analysis. The ROC curve is shown in Fig. 4.

**Table 3:** AUSROC values of each cancer by univariate (u) model and bivariate model (b).

Cancer	AUSROC-u	Low	High	P (Chi-square）	AUSROC-b
CRC					
1	0.79	0.75	0.84	0.23	0.89
NSCLC					
1	0.78	0.71	0.87	0.82	0.71
2	0.87	0.82	0.93	0.22	0.86
CRLM					
1	0.73	0.70	0.80	0.50	0.71
GC					
1	0.78	0.70	0.90	0.26	0.77
All					
1	0.80	0.77	0.82	0.44	0.80
2	0.91	0.88	0.94	0.11	0.88
Ratio					
0∼1	0.87	0.77	0.99	0.25	0.91
1∼5	0.84	0.81	0.86	0.48	0.85
5∼9	0.78	0.73	0.84	0.25	0.68
9∼13	0.77	0.70	0.85	0.26	0.71

1 = landmark detection; 2 = longitudinal detection; The ratio hierarchy is (0∼1), [1, 5), [5, 9), [9, 13); Ratio = the number of patients with ctDNA-/the number of patients with ctDNA+.

The proportion of patients with ctDNA- and ctDNA+ caused heterogeneity. We stratified the proportions and calculated the AUSROC using univariate and bivariate models. AUSROC decreased with the increase in the proportion of patients who were negative in the univariate model except for the ratio hierarchy of [9,13) (Table 3).

Total sensitivity and specificity of landmark time points were 0.50 (95% CI 0.46–0.55, I^2^ (Zhou and Dendukuri's approach) = 1.70%) and 0.91 (95% CI 0.89–0.92, I^2^ (Zhou and Dendukuri's approach) = 1.70%), respectively, while longitudinal time points were 0.74 (95% CI 0.68–0.80, I^2^ (Zhou and Dendukuri's approach) = 6.90%) and 0.89 (95% CI 0.85–0.93, I^2^ (Zhou and Dendukuri's approach) = 6.90%, Table 2). The pooled sensitivity and specificity of the detection time of all cancers were summarized in Table 2.

Subgroup analysis was also performed using monitoring techniques. Due to insufficient studies, this subgroup only analyzed landmark detection. The mPCR-NGS showed the highest sensitivity compared to the other two technologies (ddPCR and hybridization capture-based NGS). Table S9 summarizes the pooled sensitivity and specificity of detection techniques for all cancers. The AUSROC is shown in Tables S9 and S10.

#### Reporting bias analysis

The reporting bias was assessed using a funnel plot and Egger's and Begg's tests (Figure S15). The Egger's and Begg's tests results for HR of Univariate analysis of NSCLC and CRC showed that in this meta-analysis their reporting bias is non-significant (NSCLC: Univariate (u)-egger: t = 0.34, df = 23, p (t-test) = 0.7392, begg: z = 0.91, p (z-test) = 0.3624; CRC: u-egger: t (t-test) = 1.74, df = 33, p (t-test) = 0.0910, begg: z = 0.37, p (z-test) = 0.7120). However, CRLM had to report bias (egger: t = 2.58, df = 11, p (t-test) = 0.0254, begg: z = 2.50, p (z-test) = 0.0124). For multivariable analysis, the Egger's and Begg's tests results of NSCLC showed that its reporting bias is non-significant (multivariable(m)-egger: t = 0.75, df = 9, p (t-test) = 0.4711, begg: z = 0.31, p (z-test) = 0.7555) while CRC showed reporting bias (m-egger: t = 2.33, df = 22, p (t-test) = 0.0293, begg: z = 0.82, p (z-test) = 0.4130). Then, we used the Trim-and-fill method for estimating and adjusting for the number and outcomes of missing studies in CRLM-u and NSCLC-m. It was shown that pooled HR after adjusting still makes sense (CRLM-u: 3.33, 95% CI 2.61–4.23, p (z-test) < 0.001; NSCLC-m: 8.29, 95% CI 5.65–12.19, p (z-test) < 0.001). The meta-regression analysis showed that the heterogeneity was not due to the quality of studies (Tables S8 and S11).

### Sensitivity analysis

We performed a sensitivity analysis (which is used to describe supporting analyses to check the findings' robustness to the model assumptions in this paragraph) by removing each study. The sensitivity analysis results of recurrence and OS are shown in Figures S16–S20, indicating that the pooled estimates were not materially altered by using the leave-one-out sensitivity analysis.

## Discussion

The results of this study showed that ctDNA was a risk factor for resectable cancer recurrence, and its HR ranges from 3.46 to 25.86. In line with this, ctDNA is considered a risk factor for OS, HR from 3.05 to 8.69. BLCA had the highest survival benefit in the pooled HR of univariate analysis in recurrence, while ESCA had the highest survival benefit in the pooled HR of multivariable analysis, indicating that clinicopathological variables had a possible impact on the results of those studies, especially sex, age and pathological stage. Although the survival benefit of PAAD was lower than other cancers, the diagnostic sensitivity of PAAD in landmark detection was higher than that of other cancers (CRLM, GC, CRC and NSCLC). It may correlated with the low five-year survival rate in PAAD. The survival benefit of CRLM was lower than that of other cancers except PAAD, and the diagnostic sensitivity was lower than that of other cancers (PAAD and CRC) in landmark detection.

We also found an interesting phenomenon: longitudinal detection can significantly improve diagnostic efficiency. The sensitivity of longitudinal detection and AUSROC both indicate that longitudinal detection has a higher diagnostic value. From the data extracted in the included study, the time point of longitudinal detection was mostly 3–6 months after surgery, which may also be consistent with the follow-up time of surgery. However, whether this time point was the most appropriate needs to be verified; it is worth researching to find the time when negative turns to positive and the association between potentially positive patients and risk factors.

At present, ctDNA detection mainly focuses on CRC, NSCLC, GC, BC, PAAD, ESCA, CRLM, OV, BLCA, melanoma and HCC; these cancers account for about 65% of all tumor-related deaths in the world,^105^ of which CRC, NSCLC and BC were diagnosed in the early state accounted for 39%, 16% and 62%,^106^ respectively. These patients preferred radical surgical resection treatment, but the 5-year OS rate varies from cancer to cancer, OS in BC up to 99%, NSCLC only 56%.^106^ The main reason for this result may be postoperative minimal residual cancers and occult metastasis. Previous meta-analyses of single cancer all showed that the presence of ctDNA was associated with cancer recurrence (for CRC,^107^ BC,^108^ NSCLC,^109^ and ESCA,^110^ respectively). Our meta-analysis defined the time point as postoperative. The objective was to clarify the prognostic significance of the presence of ctDNA after cancer resection and to explore the significance of single and follow-up time point detection after surgery. Our meta-analysis via coincident inclusion and exclusion criteria makes comparing the results of various cancers more possible and summarizes the value of prognosis assessment and diagnostic accuracy of ctDNA-MRD.

Regarding diagnostic accuracy, the pooled AUSROC and DOR results showed higher longitudinal analysis accuracy than landmark analysis. The results of the sensitivity and specificity analysis can also be confirmed.

Multiple cancer studies have found that longitudinal detection is more sensitive than landmark detection,^63^^,^^69^^,^^75^^,^^77^^,^^92^ and our subgroup analysis result was highly consistent with this rule. In addition, the pooled HR of recurrence showed that regardless of landmark detection or post-adjuvant treatment detection, the survival benefit of longitudinal detection was the highest for BC, NSCLC, and CRC, except for ESCA.

It is not only the detection time that affects the results but also the detection technique. Previous studies have found that the detection efficiency of ddPCR and NGS was different. Loupakis F et al.^71^ and Zhang H et al.^111^ compared ddPCR with NGS technology, and it was found that NGS was more sensitive to detecting positive results. Our subgroup analysis of ctDNA detection technology also found that ddPCR was less sensitive than NGS. At the same time, we also observed that the overall sensitivity could be better, and increasing the sequencing depth and finding other ways to improve the sensitivity of NGS detection are necessary problems to be solved. In addition, the sensitivity and survival benefit of longitudinal detection of ESCA^70^ was down, and it may be associated with using a non-esophageal adenocarcinoma (EAC) cancer-specific gene panel, which was tumor-naïve method while others were tumor-informed method in ESCA. It means there is a need for specific cfDNA panels.

In addition, we found that studies with more patients with ctDNA- were less sensitive, which may understate the test's sensitivity. This means that there could be a discrepancy in the number of patients with ctDNA+ and ctDNA-. Despite the low relapse population in the ctDNA- group, they may still represent a significant portion of the relapse population.

Postoperative adjuvant therapy was a common means for patients with resectable cancer to reduce the risk of recurrence and improve the OS. Related studies^71^^,^^80^^,^^90^ showed that the survival benefit of the ctDNA+ population group would be improved after adjuvant therapy. We analyzed whether adjuvant therapy should be used. The result showed that the survival benefit of patients with ctDNA+ after adjuvant therapy increased in CRC. It suggested whether it would be correlated with the benefit of adjuvant therapy in the future, but it still needs more well-designed clinical studies.

Overall, ctDNA is very valuable for prognostic assessment and shows different prognostic values among different cancers. In addition, postoperative adjuvant therapy significantly improved the prognosis of patients with ctDNA+ in CRC. All these suggest the possibility of detecting ctDNA after surgery. Further study of the value of ctDNA+ should focus on analyzing the difference and relationship between the ctDNA-based adjuvant therapy group and the conventional management therapy group. Besides, verifying the specific longitudinal detection time point is also a problem that needs to be solved. The longitudinal detection has many complicated factors. Firstly, the relationship between the duration time of ctDNA+ and recurrence should be clarified; secondly, the different strengths of association with recurrence risk between ctDNA single positive and multiple positive should be clarified. What's more, it is necessary to explore the association between potentially positive patients and risk factors.

Limitations of our meta-analysis study: there were a few related studies on some cancers in this study, such as melanoma, HCC, and BLCA, which need to be more convincing to confirm the results. Therefore, further exploration and study in this direction are needed. In addition, there needs to be a clear definition of ctDNA-MRD and a uniform standard for NGS detection panel and sequencing depth, which may also lead to differences in study results. Lastly, there was no clear definition of ctDNA+ and ctDNA-, which may affect the false positive rate to some extent. What's more, the ratio of patients with ctDNA- and ctDNA+ affected the sensitivity estimate, which may have influenced the results of the study.

## Contributors

Two researchers (J.Z., Z.C.) conducted the study design. Three researchers (J.Z., C.Q., Q.W.) sifted through the literature, evaluated the quality of the article and extracted independent data. One researcher (J.Z.) conducted all statistical analyses. Two researchers (J.Z., Z.C.) contributed to writing the article. The senior authors (D.T., Z.C.) have directly accessed and verified the underlying data in all research articles and gave guidance on data analysis and writing. All authors critically revised the manuscript and were ultimately responsible for deciding to submit it for publication. All authors read and approved the final version of the manuscript and ensure it is the case.

## Data sharing statement

The data that support the findings of this study are included in the supplement.

## Declaration of interests

We declare no competing interests.

## Appendix A Supplementary data

Table S11

Supplement Search mode
