# Supplementary material for: Circulating tumour DNA-Based molecular residual disease detection in resectable cancers: a systematic review and meta-analysis
Source: eBioMedicine. 2024 Apr 13;103:105109. doi: 10.1016/j.ebiom.2024.105109 (PMC11021841; doi:10.1016/j.ebiom.2024.105109)
Supplement: Table S1 [file mmc1.docx]

Table S1 Quality evaluation of included studies using the Newcastle-Ottawa Scale (NOS)

|  |  |  | Selection |  |  |  | Comparability | Outcome |  |  | score |
| --- | --- | --- | --- | --- | --- | --- | --- | --- | --- | --- | --- |
|  | Year | Author | 1） | 2） | 3） | 4） |  | 1） | 2） | 3） |  |
| 1 | 2017 | Michael J. Overman | 1 | 1 | 1 | 1 | NA | 1 | 1 | NA | 6 |
| 2 | 2020 | Popova, A. | 1 | 1 | 1 | 1 | NA | 1 | NA | NA | 5 |
| 3 | 2020 | Fedyanin, M. | 1 | 1 | 1 | 1 | 2 | 1 | NA | NA | 7 |
| 4 | 2021 | Tan, A. | 1 | 1 | 1 | 1 | NA | 1 | 1 | NA | 6 |
| 5 | 2021 | Chapman, Jocelyn S | 1 | 1 | 1 | 1 | NA | 1 | 1 | NA | 6 |
| 6 | 2021 | Bryant Chee | 1 | 1 | 1 | 1 | NA | 1 | 1 | NA | 6 |
| 7 | 2021 | Anandappa, Gayathri | 1 | 1 | 1 | 1 | 2 | 1 | 1 | NA | 8 |
| 8 | 2022 | Zhou, Jian | 1 | 1 | 1 | 1 | NA | 1 | NA | NA | 5 |
| 9 | 2023 | Yuan, Shu-Qiang | 1 | 1 | 1 | 1 | 2 | 1 | 1 | 0 | 8 |
| 10 | 2022 | Han, S. W | 1 | 1 | 1 | 1 | 2 | 1 | NA | NA | 7 |
| 11 | 2022 | Genta, Sofia | 1 | 1 | 1 | 1 | NA | 1 | NA | NA | 5 |
| 12 | 2022 | Sharma, P. | 1 | 1 | 1 | 1 | NA | 1 | NA | NA | 5 |
| 13 | 2022 | McNamara, Sylvie | 1 | 1 | 1 | 1 | 2 | 1 | NA | NA | 7 |
| 14 | 2023 | Wang, Xiuchao | 1 | 1 | 1 | 1 | NA | 1 | 1 | NA | 6 |
| 15 | 2023 | Takei, Shogo | 1 | 1 | 1 | 1 | NA | 1 | NA | NA | 5 |
| 16 | 2023 | Ananda, Sumitra | 1 | 1 | 1 | 1 | 2 | 1 | NA | NA | 7 |
| 17 | 2023 | Xue, Pei | 1 | 1 | 1 | 1 | NA | 1 | 1 | NA | 6 |
| 18 | 2023 | Watanabe, Jun | 1 | 1 | 1 | 1 | 2 | 1 | 1 | NA | 8 |
| 19 | 2023 | Gerlinger, Marco | 1 | 1 | 1 | 1 | NA | 1 | 1 | NA | 6 |
| 20 | 2015 | Garcia-Murillas, Isaac | 1 | 1 | 1 | 1 | 2 | 1 | 1 | 0 | 8 |
| 21 | 2016 | Tie, J. | 1 | 1 | 1 | 1 | 2 | 1 | 1 | 1 | 9 |
| 22 | 2017 | Chen, Y. H. | 1 | 1 | 1 | 1 | 2 | 1 | 1 | 0 | 8 |
| 23 | 2017 | Schøler, L. V. | 1 | 1 | 1 | 1 | 0 | 1 | 1 | 1 | 7 |
| 24 | 2019 | Chen, K. | 1 | 1 | 1 | 1 | 0 | 1 | 1 | 1 | 7 |
| 25 | 2019 | Coombes, R. C. | 1 | 1 | 1 | 1 | 0 | 1 | 1 | 1 | 7 |
| 26 | 2019 | Lee, B. Lipton, L. | 1 | 1 | 1 | 1 | 2 | 1 | 1 | 0 | 8 |
| 27 | 2019 | Reinert, T. | 1 | 1 | 1 | 1 | 2 | 1 | 1 | 1 | 9 |
| 28 | 2019 | Tan, L. Sandhu, S. | 1 | 1 | 1 | 1 | 2 | 1 | 1 | 1 | 9 |
| 29 | 2019 | Tie, J. | 1 | 1 | 1 | 1 | 2 | 1 | 1 | 0 | 8 |
| 30 | 2019 | Tarazona, N. | 1 | 1 | 1 | 1 | 2 | 1 | 1 | 1 | 9 |
| 31 | 2019 | Christensen, Emil | 1 | 1 | 1 | 1 | 2 | 1 | 1 | 1 | 9 |
| 32 | 2020 | Jiang, J. Ye, S. | 1 | 1 | 1 | 1 | 2 | 1 | 1 | 0 | 8 |
| 33 | 2020 | Kuang, P. P | 1 | 1 | 1 | 1 | 2 | 1 | 1 | 1 | 9 |
| 34 | 2020 | Peng, M. | 1 | 1 | 1 | 1 | 2 | 1 | 1 | 1 | 9 |
| 35 | 2020 | Yang, J. | 1 | 1 | 1 | 1 | 2 | 1 | 1 | 1 | 9 |
| 36 | 2020 | Openshaw, M. R. | 1 | 1 | 1 | 1 | 0 | 1 | 1 | 1 | 7 |
| 37 | 2020 | Yang, W | 1 | 1 | 1 | 1 | 0 | 1 | 1 | 1 | 7 |
| 38 | 2021 | Chen, G. | 1 | 1 | 1 | 1 | 2 | 1 | 1 | 1 | 9 |
| 39 | 2021 | Loupakis, F. | 1 | 1 | 1 | 1 | 2 | 1 | 1 | 0 | 8 |
| 40 | 2021 | Qiu, B. | 1 | 1 | 1 | 1 | 2 | 1 | 1 | 0 | 8 |
| 41 | 2021 | Tie, J. | 1 | 1 | 1 | 1 | 2 | 1 | 1 | 1 | 9 |
| 42 | 2021 | Zhou, Y. | 1 | 1 | 1 | 1 | 0 | 1 | 1 | 1 | 7 |
| 43 | 2021 | Liu, T. | 1 | 1 | 1 | 1 | 2 | 1 | 1 | 0 | 8 |
| 44 | 2021 | Wang, D. S. | 1 | 1 | 1 | 1 | 2 | 1 | 1 | 0 | 8 |
| 45 | 2021 | Benhaim, L. | 1 | 1 | 1 | 1 | 2 | 1 | 1 | 0 | 8 |
| 46 | 2021 | Ococks, E | 1 | 1 | 1 | 1 | 2 | 1 | 1 | 1 | 9 |
| 47 | 2022 | Newhook, T. E | 1 | 1 | 1 | 1 | 0 | 1 | 1 | 1 | 7 |
| 48 | 2022 | Carrasco, R | 1 | 1 | 1 | 1 | 2 | 1 | 1 | 0 | 8 |
| 49 | 2022 | Henriksen, T. V. | 1 | 1 | 1 | 1 | 2 | 1 | 1 | 1 | 9 |
| 50 | 2022 | Li, N. | 1 | 1 | 1 | 1 | 0 | 1 | 1 | 1 | 7 |
| 51 | 2022 | Li, Y. | 1 | 1 | 1 | 1 | 2 | 1 | 1 | 1 | 9 |
| 52 | 2022 | Nishioka, Y. | 1 | 1 | 1 | 1 | 2 | 1 | 1 | 0 | 8 |
| 53 | 2022 | Reinert, T. | 1 | 1 | 1 | 1 | 2 | 1 | 1 | 1 | 9 |
| 54 | 2022 | Szabados, B. | 1 | 1 | 1 | 1 | 0 | 1 | 1 | 1 | 7 |
| 55 | 2022 | Wang, S. | 1 | 1 | 1 | 1 | 2 | 1 | 1 | 1 | 9 |
| 56 | 2022 | Xia, L. | 1 | 1 | 1 | 1 | 2 | 1 | 1 | 1 | 9 |
| 57 | 2022 | Zhao, L. | 1 | 1 | 1 | 1 | 2 | 1 | 1 | 1 | 9 |
| 58 | 2022 | Hata, Tatsuo | 1 | 1 | 1 | 1 | 2 | 1 | 1 | 0 | 8 |
| 59 | 2022 | Chao, A | 1 | 1 | 1 | 1 | 2 | 1 | 1 | 1 | 9 |
| 60 | 2022 | Zhang, J. T | 1 | 1 | 1 | 1 | 2 | 1 | 1 | 1 | 9 |
| 61 | 2022 | Waldeck, S. | 1 | 1 | 1 | 1 | 2 | 1 | 1 | 0 | 8 |
| 62 | 2023 | Chen, K | 1 | 1 | 1 | 1 | 2 | 1 | 1 | 0 | 8 |
| 63 | 2023 | Fu, R. | 1 | 1 | 1 | 1 | 2 | 1 | 1 | 1 | 9 |
| 64 | 2023 | Kotani, D. | 1 | 1 | 1 | 1 | 2 | 1 | 1 | 1 | 9 |
| 65 | 2023 | Mo, S. | 1 | 1 | 1 | 1 | 2 | 1 | 1 | 1 | 9 |
| 66 | 2023 | Morimoto, Y | 1 | 1 | 1 | 1 | 0 | 1 | 1 | 1 | 7 |
| 67 | 2023 | Hofste, Lisa S. M. | 1 | 1 | 1 | 1 | 0 | 1 | 1 | 0 | 6 |
| 68 | 2023 | Liu, W | 1 | 1 | 1 | 1 | 2 | 1 | 1 | 0 | 8 |
| 69 | 2023 | Jiang, H | 1 | 1 | 1 | 1 | 0 | 1 | 1 | 0 | 6 |
| 70 | *2023 | Chen, K | 1 | 1 | 1 | 1 | 2 | 1 | 1 | 0 | 8 |
| 71 | 2022 | Kitahata, Y | 1 | 1 | 1 | 1 | 1 | 1 | 1 | 1 | 8 |
| 72 | 2021 | Yamaguchi, T. | 1 | 1 | 1 | 1 | 2 | 1 | 1 | 1 | 9 |
| 73 | 2022 | Tie, J | 1 | 1 | 1 | 1 | 0 | 1 | 1 | 1 | 7 |
| 74 | 2020 | Leal, A. | 1 | 1 | 1 | 1 | 0 | 1 | 1 | 1 | 7 |
| 75 | 2021 | Taieb, J. | 1 | 1 | 1 | 1 | 2 | 1 | 1 | 1 | 9 |
| 76 | 2021 | Powles, T. | 1 | 1 | 1 | 1 | 2 | 1 | 1 | 1 | 9 |
| 77 | 2023 | Powles, T. | 1 | 1 | 1 | 1 | 2 | 1 | 1 | 1 | 9 |
| 78 | 2022 | Schneider, B. P. | 1 | 1 | 1 | 1 | 2 | 1 | 1 | 0 | 8 |
| 79 | 2023 | Eroglu, Zeynep | 1 | 1 | 1 | 1 | 0 | 1 | 1 | 1 | 7 |
| 80 | 2021 | Bolhuis, K. | 1 | 1 | 1 | 1 | 2 | 1 | 1 | 1 | 9 |

NA: Not Available.
