# Supplementary material for: Circulating tumour DNA-Based molecular residual disease detection in resectable cancers: a systematic review and meta-analysis
Source: eBioMedicine. 2024 Apr 13;103:105109. doi: 10.1016/j.ebiom.2024.105109 (PMC11021841; doi:10.1016/j.ebiom.2024.105109)
Supplement: Table S2 [file mmc2.docx]

Table S2 The distribution of countries of studies

|  |  |  |  |
| --- | --- | --- | --- |
|  | **Country** |  |  |
|  | China | 28(36%) |  |
|  | England | 11(14%) |  |
|  | Australia | 8(10%) |  |
|  | America | 8(10%) |  |
|  | Japan | 7(9%) |  |
|  | Denmark | 3(4%) |  |
|  | Spain | 2(3%) |  |
|  | Russia | 2(3%) |  |
|  | Canada | 1(1%) |  |
|  | Denmark and Spain | 1(1%) |  |
|  | France | 1(1%) |  |
|  | Germany | 1(1%) |  |
|  | India | 1(1%) |  |
|  | Italy | 1(1%) |  |
|  | Korea | 1(1%) |  |
|  | Netherland | 1(1%) |  |
|  | Netherland, Denmark and Sweden | 1(1%) |  |
|  | Singapore | 1(1%) |  |
|  | Unknown | 1(1%) |  |
|  | **Table S2.** **The distribution of countries of studies** |  |  |
