# Supplementary material for: Circulating tumour DNA-Based molecular residual disease detection in resectable cancers: a systematic review and meta-analysis
Source: eBioMedicine. 2024 Apr 13;103:105109. doi: 10.1016/j.ebiom.2024.105109 (PMC11021841; doi:10.1016/j.ebiom.2024.105109)
Supplement: Table S3 [file mmc3.docx]

Table S3 The data and main features of the article included (HR for Univariate analysis)

|  | | |  |  |  |  |  |  | Recurrence | OS | RFS |  |  | DFS |  |  | OS |  |  | PFS |  |  | EFS/DMFS | | |
| --- | --- | --- | --- | --- | --- | --- | --- | --- | --- | --- | --- | --- | --- | --- | --- | --- | --- | --- | --- | --- | --- | --- | --- | --- | --- |
| Year | Study | Cancer | Adj | Time | Sample | Negative | Positive | Sex (female/male) | N of event | N of event | HR | HR_LL | HR_UL | HR | HR_LL | HR_UL | HR | HR_LL | HR_UL | HR | HR_LL | HR_UL | HR | HR_LL | HR_UL |
| 2021 | Taieb, J | CRC | —— | 1 | 1017 | 877 | 140 | 441/576 | 1017 (298) | 1017 (184) | —— | —— | —— | 1.46 | 1.08 | 1.97 | 1.56 | 1.08 | 2.26 | —— | —— | —— | —— | —— | —— |
| 2022 | Tie, J | CRC | —— | 1 | 291 | 246 | 45 | ——/—— | 291 (23) | —— | 1.83 | 0.79 | 4.27 | —— | —— | —— | —— | —— | —— | —— | —— | —— | —— | —— | —— |
|  |  | CRC | —— | 1 | 291 | 246 | 45 | ——/—— | 291 (22) | —— | 2.45 | 1 | 5.99 | —— | —— | —— | —— | —— | —— | —— | —— | —— | —— | —— | —— |
| 2022 | Li,Y | CRC | —— | 1 | 151 | 127 | 24 | ——/—— | 151 (39) | —— | 5.306 | 2.561 | 10.992 | —— | —— | —— | —— | —— | —— | —— | —— | —— | —— | —— | —— |
|  |  | CRC | yes | 3 | 124 | 113 | 11 | ——/—— | 124 (36) | —— | 2.75 | 1.06 | 7.134 | —— | —— | —— | —— | —— | —— | —— | —— | —— | —— | —— | —— |
| 2021 | Loupakis, F | CRC | yes | 1 | 112 | 51 | 61 | 40/72 | 112 (82) | 112 (——) | —— | —— | —— | 5.8 | 3.5 | 9.7 | 16 | 3.9 | 68 | —— | —— | —— | —— | —— | —— |
|  |  | CRC | no | 1 | 47 | 17 | 30 | ——/—— | 47 (35) | —— | —— | —— | —— | 15 | 4.3 | 49 | —— | —— | —— | —— | —— | —— | —— | —— | —— |
| 2019 | Tarazona, N | CRC | —— | 1 | 69 | 55 | 14 | ——/—— | 69 (18) | —— | —— | —— | —— | 6.96 | 2.57 | 18.91 | —— | —— | —— | —— | —— | —— | —— | —— | —— |
|  |  | CRC | —— | 2 | 94 | 62 | 32 | 33/61 | 94 (18) | —— | —— | —— | —— | 8.03 | 1.79 | 35.98 | —— | —— | —— | —— | —— | —— | —— | —— | —— |
|  |  | CRC | yes | 3 | 25 | 18 | 7 | ——/—— | 25 (10) | —— | —— | —— | —— | 10.02 | 9.202 | 307.3 | —— | —— | —— | —— | —— | —— | —— | —— | —— |
| 2022 | Henriksen, T. V | CRC | —— | 1 | 140 | 120 | 20 | ——/—— | 140 (38) | —— | 7 | 3.7 | 13.5 | —— | —— | —— | —— | —— | —— | —— | —— | —— | —— | —— | —— |
|  |  | CRC | —— | 2 | 114 | 92 | 22 | ——/—— | 114 (24) | —— | 50.8 | 14.9 | 172 | —— | —— | —— | —— | —— | —— | —— | —— | —— | —— | —— | —— |
|  |  | CRC | —— | 3 | 93 | 83 | 10 | ——/—— | 93 (20) | —— | 50.76 | 15.4 | 167 | —— | —— | —— | —— | —— | —— | —— | —— | —— | —— | —— | —— |
| 2019 | Reinert, T | CRC | —— | 1 | 94 | 84 | 10 | ——/—— | 94 (17) | —— | 7.2 | 2.7 | 19 | —— | —— | —— | —— | —— | —— | —— | —— | —— | —— | —— | —— |
|  |  | CRC | yes | 3 | 58 | 51 | 7 | 13/45 | 58 (14) | —— | 17.5 | 5.4 | 56.5 | —— | —— | —— | —— | —— | —— | —— | —— | —— | —— | —— | —— |
|  |  | CRC | —— | 2 | 75 | 60 | 15 | ——/—— | 75 (16) | —— | 43.5 | 9.8 | 193.5 | —— | —— | —— | —— | —— | —— | —— | —— | —— | —— | —— | —— |
| 2021 | Bryant,C | CRC | —— | 1 | 45 | 20 | 25 | ——/—— | 45 (27) | —— | 7.7 | 2.6 | 22.5 | —— | —— | —— | —— | —— | —— | —— | —— | —— | —— | —— | —— |
| 2022 | Han, S. W. | CRC | —— | 1 | 71 | 58 | 13 | ——/—— | 71 (23) | —— | 8.23 | 3.3 | 21 | —— | —— | —— | —— | —— | —— | —— | —— | —— | —— | —— | —— |
| 2021 | Anandappa, Gayathri | CRC | —— | 1 | 64 | 53 | 11 | ——/—— | 64 (14) | —— | 9 | 2.6 | 32 | —— | —— | —— | —— | —— | —— | —— | —— | —— | —— | —— | —— |
| 2023 | Kotani, D | CRC | —— | 1 | 1039 | 852 | 187 | 489/550 | 1039 (196) | —— | —— | —— | —— | 10 | 7.7 | 14 | —— | —— | —— | —— | —— | —— | —— | —— | —— |
| 2023 | Hofste, Lisa S. M. | CRC | —— | 1 | 19 | 16 | 3 | ——/—— | 19 (5) | —— | —— | —— | —— | —— | —— | —— | —— | —— | —— | 10.9 | 1.1 | 106.7 | —— | —— | —— |
| 2021 | Chen, G | CRC | —— | 1 | 240 | 220 | 20 | 106/134 | 240 (23) | —— | 10.98 | 5.31 | 22.72 | —— | —— | —— | —— | —— | —— | —— | —— | —— | —— | —— | —— |
|  |  | CRC | yes | 3 | 137 | 125 | 12 | ——/—— | 137 (——) | —— | 12.76 | 5.39 | 30.19 | —— | —— | —— | —— | —— | —— | —— | —— | —— | —— | —— | —— |
|  |  | CRC | —— | 2 | 125 | 100 | 25 | ——/—— | 125 (——) | —— | 32.02 | 10.79 | 95.08 | —— | —— | —— | —— | —— | —— | —— | —— | —— | —— | —— | —— |
| 2019 | Tie, J | CRC | all | 1 | 159 | 140 | 19 | 52/107 | 159 (23) | —— | 13 | 5.5 | 31 | —— | —— | —— | —— | —— | —— | —— | —— | —— | —— | —— | —— |
|  |  | CRC | yes | 1 | 102 | 91 | 11 | ——/—— | 102 (——) | —— | 10 | 3.4 | 29 | —— | —— | —— | —— | —— | —— | —— | —— | —— | —— | —— | —— |
|  |  | CRC | no | 1 | 57 | 49 | 8 | ——/—— | 57 (——) | —— | 22 | 4.2 | 110 | —— | —— | —— | —— | —— | —— | —— | —— | —— | —— | —— | —— |
| 2016 | Tie, J | CRC | no | 1 | 178 | 164 | 14 | ——/—— | 178 (27) | —— | 18 | 7.9 | 40 | —— | —— | —— | —— | —— | —— | —— | —— | —— | —— | —— | —— |
|  |  | CRC | all | 1 | 230 | 210 | 20 | 99/131 | 230 (——) | —— | 13 | 6.6 | 27 | —— | —— | —— | —— | —— | —— | —— | —— | —— | —— | —— | —— |
|  |  | CRC | yes | 1 | 44 | 41 | 3 | ——/—— | 44 (——) | —— | 11 | 1.8 | 68 | —— | —— | —— | —— | —— | —— | —— | —— | —— | —— | —— | —— |
| 2023 | Watanabe, Jun | CRC | —— | 1 | 2083 | 1797 | 286 | ——/—— | 2083 (——) | —— | —— | —— | —— | 12 | 9.1 | 15 | —— | —— | —— | —— | —— | —— | —— | —— | —— |
| 2022 | Zhou, Jian | CRC | —— | 1 | 40 | 34 | 6 | ——/—— | 40 (——) | —— | —— | —— | —— | 16.57 | 3.01 | 91.36 | —— | —— | —— | —— | —— | —— | —— | —— | —— |
| 2023 | Mo, S. | CRC | —— | 1 | 255 | 196 | 59 | ——/—— | 255 (50) | —— | 17.5 | 8.9 | 34.4 | —— | —— | —— | —— | —— | —— | —— | —— | —— | —— | —— | —— |
|  |  | CRC | —— | 2 | 226 | 179 | 47 | ——/—— | 226 (——) | —— | 13.5 | 5.4 | 33.8 | —— | —— | —— | —— | —— | —— | —— | —— | —— | —— | —— | —— |
|  |  | CRC | yes | 3 | 149 | 131 | 18 | ——/—— | 149 (22) | —— | 13.8 | 5.9 | 32.1 | —— | —— | —— | —— | —— | —— | —— | —— | —— | —— | —— | —— |
| 2022 | Li, N | NSCLC | —— | 1 | 116 | 104 | 12 | ——/—— | 116 (——) | 116 (——) | 3.04 | 1.22 | 7.58 | —— | —— | —— | 4.04 | 0.74 | 22.09 | —— | —— | —— | —— | —— | —— |
|  |  | NSCLC | —— | 2 | 119 | 82 | 37 | 49/70 | 119 (28) | 119 (6) | 3.46 | 1.59 | 7.55 | —— | —— | —— | 9.99 | 1.17 | 85.78 | —— | —— | —— | —— | —— | —— |
| 2020 | Peng, M | NSCLC | —— | 2 | 71 | 41 | 30 | 20/51 | 71 (31) | 71 (25) | 3.108 | 1.474 | 6.553 | —— | —— | —— | 3.223 | 1.348 | 7.707 | —— | —— | —— | —— | —— | —— |
| 2020 | Kuang, P. P. | NSCLC | —— | 1 | 35 | 27 | 8 | ——/—— | 35 (9) | —— | 3.69 | 1.1112 | 12.254 | —— | —— | —— | —— | —— | —— | —— | —— | —— | —— | —— | —— |
|  |  | NSCLC | yes | 3 | 36 | 28 | 8 | ——/—— | 36 (9) | —— | 8.76 | 1.63 | 47.01 | —— | —— | —— | —— | —— | —— | —— | —— | —— | —— | —— | —— |
| 2022 | Wang, S | NSCLC | —— | 1 | 116 | 100 | 16 | ——/—— | 116 (——) | —— | 3.9 | 1.85 | 8.2 | —— | —— | —— | —— | —— | —— | —— | —— | —— | —— | —— | —— |
|  |  | NSCLC | —— | 2 | 117 | 77 | 40 | ——/—— | 117 (34) | —— | 7.59 | 3.53 | 16.32 | —— | —— | —— | —— | —— | —— | —— | —— | —— | —— | —— | —— |
|  |  | NSCLC | —— | 1 | 114 | 98 | 16 | ——/—— | 114 (——) | —— | 4.32 | 2.06 | 9.08 | —— | —— | —— | —— | —— | —— | —— | —— | —— | —— | —— | —— |
|  |  | NSCLC | —— | 1 | 89 | 78 | 11 | ——/—— | 89 (——) | —— | 6.19 | 2.44 | 15.69 | —— | —— | —— | —— | —— | —— | —— | —— | —— | —— | —— | —— |
| 2021 | Qiu, B | NSCLC | —— | 1 | 85 | 67 | 18 | ——/—— | 85 (33) | —— | 3.95 | 1.96 | 7.96 | —— | —— | —— | —— | —— | —— | —— | —— | —— | —— | —— | —— |
|  |  | NSCLC | yes | 3 | 64 | 56 | 8 | ——/—— | 64 (——) | —— | 3.22 | 1.26 | 8.18 | —— | —— | —— | —— | —— | —— | —— | —— | —— | —— | —— | —— |
|  |  | NSCLC | —— | 2 | 89 | 54 | 35 | ——/—— | 89 (34) | —— | 8.55 | 3.7 | 19.74 | —— | —— | —— | —— | —— | —— | —— | —— | —— | —— | —— | —— |
| 2023 | Chen, K | NSCLC | —— | 1 | 148 | 125 | 23 | ——/—— | 148 (——) | —— | —— | —— | —— | 4.13 | 1.9 | 9 | —— | —— | —— | —— | —— | —— | —— | —— | —— |
|  |  | NSCLC | —— | 2 | 63 | 55 | 8 | ——/—— | 63 (——) | —— | —— | —— | —— | 15.14 | 4.45 | 51.45 | —— | —— | —— | —— | —— | —— | —— | —— | —— |
| 2023 | Fu, R | NSCLC | —— | 1 | 146 | 110 | 36 | ——/—— | 146 (38) | 146 (——) | —— | —— | —— | 5.07 | 2.33 | 11.01 | 4.43 | 0.76 | 25.66 | —— | —— | —— | —— | —— | —— |
|  |  | NSCLC | —— | 2 | 177 | 122 | 55 | 81/96 | 177 (41) | 117 (——) | —— | —— | —— | 7.14 | 3.6 | 14.15 | 13.77 | 2.75 | 68.95 | —— | —— | —— | —— | —— | —— |
| 2020 | Yang, W. | NSCLC | —— | 2 | 82 | 67 | 15 | 49/33 | 82 (5) | —— | —— | —— | —— | 8.5 | 1.3 | 56.3 | —— | —— | —— | —— | —— | —— | —— | —— | —— |
| 2021 | Tan, A. | NSCLC | —— | 2 | 57 | 49 | 8 | 23/34 | 57 (——) | —— | 22 | 3.6 | 133.8 | —— | —— | —— | —— | —— | —— | —— | —— | —— | —— | —— | —— |
| 2019 | Chen, K | NSCLC | —— | 1 | 25 | 18 | 7 | ——/—— | 25 (9) | 25 (——) | 7.552 | 2.092 | 27.266 | —— | —— | —— | 14.22 | 1.577 | 128.15 | —— | —— | —— | —— | —— | —— |
| *2023 | Chen, K | NSCLC | —— | 1 | 156 | 137 | 19 | ——/—— | 156 (——) | 156 (——) | —— | —— | —— | 16.4 | 7.84 | 34.31 | 9.5 | 3.04 | 29.67 | —— | —— | —— | —— | —— | —— |
|  |  | NSCLC | —— | 2 | 110 | 87 | 23 | 56/54 | 110 (19) | 110 (——) | —— | —— | —— | 37.42 | 10.76 | 130.16 | 21.98 | 2.57 | 188.25 | —— | —— | —— | —— | —— | —— |
| 2022 | Waldeck, S | NSCLC | —— | 1 | 16 | 12 | 4 | ——/—— | 16 (8) | 16 (——) | —— | —— | —— | —— | —— | —— | 33.33 | 3.04 | 365.32 | 10.64 | 1.65 | 68.76 | —— | —— | —— |
| 2022 | Xia, L | NSCLC | —— | 1 | 329 | 303 | 26 | ——/—— | 329 (70) | —— | 11.1 | 6.5 | 19 | —— | —— | —— | —— | —— | —— | —— | —— | —— | —— | —— | —— |
| 2022 | Zhang, J. T. | NSCLC | —— | 1 | 245 | 224 | 21 | ——/—— | 245 (47) | —— | —— | —— | —— | 12.5 | 3.08 | 50.6 | —— | —— | —— | —— | —— | —— | —— | —— | —— |
|  |  | NSCLC | —— | 2 | 236 | 190 | 46 | ——/—— | 236 (47) | —— | —— | —— | —— | 50 | 25 | 100 | —— | —— | —— | —— | —— | —— | —— | —— | —— |
| 2023 | Yuan, Shu-Qiang | GC | —— | 1 | 100 | 75 | 25 | 34/68 | 100 (33) | 100 (——) | 2.74 | 1.37 | 5.48 | —— | —— | —— | 2.53 | 1.17 | 5.45 | —— | —— | —— | —— | —— | —— |
|  |  | GC | —— | 3 | 41 | 31 | 10 | 15/26 | 41 (24) | 41 (——) | 14.99 | 3.08 | 72.96 | —— | —— | —— | 11.88 | 2.38 | 59.24 | —— | —— | —— | —— | —— | —— |
| 2020 | Leal, A. | GC | yes | 1 | 50 | 11 | 9 | ——/—— | 50 (6) | 50 (——) | —— | —— | —— | 21.8 | 3.9 | 123.1 | 21.8 | 3.9 | 123.1 | —— | —— | —— | —— | —— | —— |
| 2020 | Yang, J. | GC | —— | 1 | 38 | 31 | 7 | ——/—— | 38 (17) | 38 (——) | —— | —— | —— | 7.542 | 2.682 | 21.213 | 6.224 | 1.864 | 20.78 | —— | —— | —— | —— | —— | —— |
|  |  | GC | —— | 2 | 44 | 27 | 17 | ——/—— | 44 (36) | 44 (13) | —— | —— | —— | 17.873 | 5.011 | 63.755 | 7.95 | 2.169 | 29.139 | —— | —— | —— | —— | —— | —— |
|  |  | GC | —— | 3 | 23 | 18 | 5 | ——/—— | 23 (——) | 23 (——) | —— | —— | —— | 9.866 | 2.278 | 42.741 | 22.23 | 2.448 | 201.86 | —— | —— | —— | —— | —— | —— |
| 2023 | Xue, Pei | GC | —— | 1 | 13 | 12 | 1 | ——/—— | 13 (3) | —— | 8 | 1.3875 | 46.1264 | —— | —— | —— | —— | —— | —— | —— | —— | —— | —— | —— | —— |
| 2022 | Hata, Tatsuo | PAAD | —— | 1 | 66 | 50 | 16 | 21/45 | 66 (——) | 66 (——) | —— | —— | —— | 2.11 | 1.039 | 4.284 | 2.726 | 1.112 | 6.682 | —— | —— | —— | —— | —— | —— |
| 2020 | Popova, A. | PAAD | —— | 1 | 37 | 23 | 14 | ——/—— | 37 (10) | —— | —— | —— | —— | —— | —— | —— | —— | —— | —— | 2.9 | 1.01 | 8.5 | —— | —— | —— |
| 2020 | Jiang, J. | PAAD | —— | 1 | 27 | 18 | 9 | 10/17 | 27 (14) | —— | —— | —— | —— | 3.55 | 0.9 | 13.89 | —— | —— | —— | —— | —— | —— | —— | —— | —— |
| 2019 | Lee, B | PAAD | —— | 1 | 35 | 22 | 13 | 14/21 | 35 (23) | 35 (——) | 5.4 | 1.9 | 15.2 | —— | —— | —— | 5.5 | 1 | 17.4 | —— | —— | —— | —— | —— | —— |
| 2023 | Wang, Xiuchao | PAAD | —— | 1 | 17 | 15 | 2 | ——/—— | 17 (4) | —— | —— | —— | —— | 16.87 | 2.513 | 113.2389 | —— | —— | —— | —— | —— | —— | —— | —— | —— |
| 2022 | Kitahata, Y | PAAD | —— | 1 | 27 | 13 | 14 | 11/15 | —— | 27 (——) | —— | —— | —— | —— | —— | —— | 5.019 | 1.228 | 20.515 | —— | —— | —— | —— | —— | —— |
| 2021 | Wang, D. S | CRLM | —— | 1 | 82 | 48 | 34 | 29/53 | 82 (47) | —— | 2.67 | 1.49 | 4.8 | —— | —— | —— | —— | —— | —— | —— | —— | —— | —— | —— | —— |
|  |  | CRLM | —— | 3 | 49 | 27 | 22 | 19/30 | 49 (12) | —— | 2.46 | 1.15 | 5.28 | —— | —— | —— | —— | —— | —— | —— | —— | —— | —— | —— | —— |
| 2023 | Liu, W | CRLM | —— | 1 | 134 | 92 | 42 | 36/98 | 134 (84) | 134 (——) | —— | —— | —— | 2.96 | 1.91 | 4.6 | 11.5 | 3.28 | 40.6 | —— | —— | —— | —— | —— | —— |
| 2022 | Nishioka, Y. | CRLM | —— | 1 | 105 | 73 | 32 | 42/63 | 105 (66) | —— | 3.1 | 1.92 | 5.01 | —— | —— | —— | —— | —— | —— | —— | —— | —— | —— | —— | —— |
| 2017 | Michael J | CRLM | —— | 1 | 54 | 30 | 24 | ——/—— | 54 (——) | —— | 3.1 | 1.7 | 9.1 | —— | —— | —— | —— | —— | —— | —— | —— | —— | —— | —— | —— |
| 2022 | Newhook, T. E | CRLM | —— | 1 | 48 | 30 | 18 | ——/—— | 48 (34) | —— | 3.23 | 1.6066 | 6.4939 | —— | —— | —— | —— | —— | —— | —— | —— | —— | —— | —— | —— |
| 2021 | Bolhuis, K. | CRLM | yes | 1 | 23 | 17 | 6 | 8/15 | 23 (17) | —— | 3.3 | 1.1 | 9.6 | —— | —— | —— | —— | —— | —— | —— | —— | —— | —— | —— | —— |
| 2023 | Jiang, H | CRLM | yes | 1 | 67 | 52 | 15 | 29/38 | 67 (41) | —— | 3.596 | 1.479 | 8.744 | —— | —— | —— | —— | —— | —— | —— | —— | —— | —— | —— | —— |
| 2017 | Schøler, L. V | CRLM | —— | 1 | 21 | 15 | 6 | 6/15 | 21 (10) | 21 (——) | 4.9 | 1.5 | 15.7 | —— | —— | —— | 6.7 | 1.6 | 28.7 | —— | —— | —— | —— | —— | —— |
| 2021 | Tie, J | CRLM | —— | 1 | 49 | 37 | 12 | 14/35 | 49 (21) | 49 (——) | 6.31 | 2.59 | 15.37 | —— | —— | —— | 4.2 | 1.5 | 11.8 | —— | —— | —— | —— | —— | —— |
|  |  | CRLM | —— | 3 | 45 | 34 | 11 | ——/—— | 45 (——) | 45 (——) | 14.9 | 4.94 | 44.7 | —— | —— | —— | 5.54 | 1.83 | 16.8 | —— | —— | —— | —— | —— | —— |
| 2022 | Reinert, T | CRLM | —— | 1 | 40 | 27 | 13 | ——/—— | 40 (28) | —— | 7.6 | 3 | 19.7 | —— | —— | —— | —— | —— | —— | —— | —— | —— | —— | —— | —— |
|  |  | CRLM | —— | 2 | 67 | 33 | 34 | ——/—— | 67 (47) | —— | 4.3 | 2.2 | 8.1 | —— | —— | —— | —— | —— | —— | —— | —— | —— | —— | —— | —— |
| 2022 | Sharma, P | BC | —— | 1 | 47 | 31 | 16 | ——/—— | 47 (14) | 47 (13) | —— | —— | —— | —— | —— | —— | 3.05 | 1.02 | 9.13 | —— | —— | —— | 3.02 | 1.01 | 9.01 |
| 2019 | Coombes, R. C. | BC | —— | 1 | 49 | 40 | 9 | ——/—— | 49 (18) | —— | 11.8 | 4.3 | 32.5 | —— | —— | —— | —— | —— | —— | —— | —— | —— | —— | —— | —— |
|  |  | BC | —— | 2 | 49 | 33 | 16 | ——/—— | 49 (18) | —— | 35.8 | 8 | 161.3 | —— | —— | —— | —— | —— | —— | —— | —— | —— | —— | —— | —— |
| 2017 | Chen, Y. H. | BC | —— | 1 | 33 | 29 | 4 | ——/—— | 33 (13) | —— | —— | —— | —— | 12.6 | 3.06 | 52.2 | —— | —— | —— | —— | —— | —— | —— | —— | —— |
| 2021 | Zhou, Y | BC | yes | 1 | 32 | 27 | 5 | ——/—— | 32 (7) | —— | —— | —— | —— | 23.53 | 1.904 | 290.9 | —— | —— | —— | —— | —— | —— | —— | —— | —— |
| 2015 | Garcia-Murillas, Isaac | BC | —— | 1 | 37 | 30 | 7 | 37/0 | 37 (12) | —— | —— | —— | —— | 25.1 | 4.08 | 130.5 | —— | —— | —— | —— | —— | —— | —— | —— | —— |
|  |  | BC | —— | 2 | 43 | 30 | 13 | 43/0 | 43 (15) | —— | —— | —— | —— | 12 | 3.36 | 43.07 | —— | —— | —— | —— | —— | —— | —— | —— | —— |
| 2020 | Openshaw, M. R. | ESCA | —— | 1 | 22 | 16 | 6 | ——/—— | 22 (13) | —— | 3.7 | 1.1518 | 11.8859 | —— | —— | —— | —— | —— | —— | —— | —— | —— | —— | —— | —— |
|  |  | ESCA | —— | 2 | 22 | 13 | 9 | ——/—— | 22 (13) | —— | 5.9 | 1.6635 | 20.9263 | —— | —— | —— | —— | —— | —— | —— | —— | —— | —— | —— | —— |
| 2023 | Takei, Shogo | ESCA | —— | 1 | 28 | 25 | 3 | ——/—— | 28 (——) | —— | 14 | 3 | 62 | —— | —— | —— | —— | —— | —— | —— | —— | —— | —— | —— | —— |
| 2023 | Morimoto, Y. | ESCA | —— | 1 | 16 | 10 | 6 | 3/13 | 16 (7) | —— | 16.9 | 1.92 | 149.4 | —— | —— | —— | —— | —— | —— | —— | —— | —— | —— | —— | —— |
| 2023 | Gerlinger, Marco | ESCA | —— | 1 | 24 | 18 | 6 | ——/—— | 24 (——) | —— | —— | —— | —— | —— | —— | —— | —— | —— | —— | 27 | 3 | 241 | —— | —— | —— |
| 2021 | Liu, T. | ESCA | —— | 1 | 23 | 19 | 4 | ——/—— | 23 (5) | 23 (5) | —— | —— | —— | 27.5 | 2.8 | 273.1 | 27.6 | 2.9 | 259.1 | —— | —— | —— | —— | —— | —— |
| 2021 | Ococks, E. | ESCA | —— | 2 | 63 | 53 | 10 | ——/—— | 63 (26) | 63 (——) | —— | —— | —— | 5.35 | 2.1 | 13.63 | 5.55 | 2.42 | 12.71 | —— | —— | —— | —— | —— | —— |
| 2023 | Ananda, Sumitra | OV | —— | 1 | 81 | 18 | 63 | ——/—— | 81 (——) | —— | 3.28 | 1.39 | 7.72 | —— | —— | —— | —— | —— | —— | —— | —— | —— | —— | —— | —— |
| 2022 | Chao, A. | OV | —— | 1 | 29 | 18 | 11 | ——/—— | 29 (8) | 29 (——) | —— | —— | —— | —— | —— | —— | 4.18 | 0.97 | 18.06 | 5.34 | 1.87 | 15.27 | —— | —— | —— |
| 2021 | Chapman, Jocelyn S. | OV | —— | 1 | 20 | 13 | 7 | ——/—— | 20 (7) | —— | 12.75 | 1.7 | 94 | —— | —— | —— | —— | —— | —— | —— | —— | —— | —— | —— | —— |
| 2021 | Powles, T | BLCA | no | 1 | 281 | 183 | 98 | 62/221 | 281 (——) | —— | —— | —— | —— | 6.3 | 4.45 | 8.92 |  |  |  | —— | —— | —— | —— | —— | —— |
| 2023 | Powles, T | BLCA | no | 1 | 281 | 183 | 98 | 62/221 | —— | 281 (——) | —— | —— | —— | —— | —— | —— | 6.3 | 4.3 | 9.3 | —— | —— | —— | —— | —— | —— |
| 2022 | Szabados, B. | BLCA | —— | 1 | 36 | 31 | 5 | ——/—— | 36 (6) | —— | 78.22 | 8.64 | 707.78 | —— | —— | —— | —— | —— | —— | —— | —— | —— | —— | —— | —— |
| 2019 | Christensen, Emil | BLCA | —— | 2 | 64 | 47 | 17 | ——/—— | 64 (13) | —— | 131.3 | 16.6 | 16,993.60 | —— | —— | —— | —— | —— | —— | —— | —— | —— | —— | —— | —— |
| 2019 | Tan, L. | melanoma | —— | 1 | 52 | 39 | 13 | ——/—— | 52 (29) | 52 (——) | 10 | 4.3 | 24 | —— | —— | —— | 1.4 | 0.23 | 9 | —— | —— | —— | —— | —— | —— |
| 2022 | Genta, Sofia | melanoma | —— | 1 | 45 | 41 | 4 | 12/33 | 45 (——) | 45 (——) | —— | —— | —— | —— | —— | —— | 8.9 | 1.45 | 54.77 | 2.71 | 0.6 | 12.31 | —— | —— | —— |
| 2023 | Eroglu, Zeynep | melanoma | —— | 1 | 29 | 24 | 5 | 9/20 | 29 (——) | —— | —— | —— | —— | —— | —— | —— | —— | —— | —— | —— | —— | —— | 10.77 | 1.77 | 65.57 |
| 2022 | Zhao, L | HCC | —— | 2 | 59 | 38 | 21 | ——/—— | 59 (27) | —— | 11.77 | 4.96 | 27.96 | —— | —— | —— | —— | —— | —— | —— | —— | —— | —— | —— | —— |

1=landmark detection; 2=longitudinal detection; 3=post-adjuvant therapy; Adj= Adjuvant therapy.
