# Supplementary material for: Circulating tumour DNA-Based molecular residual disease detection in resectable cancers: a systematic review and meta-analysis
Source: eBioMedicine. 2024 Apr 13;103:105109. doi: 10.1016/j.ebiom.2024.105109 (PMC11021841; doi:10.1016/j.ebiom.2024.105109)
Supplement: Table S4 [file mmc4.docx]

Table S4 The data and main features of the article are included (HR for Multivariable analysis)

|  | | |  |  |  |  |  |  | Recurrence | OS | RFS |  |  | DFS |  |  | OS |  |  | PFS/TTR | | |
| --- | --- | --- | --- | --- | --- | --- | --- | --- | --- | --- | --- | --- | --- | --- | --- | --- | --- | --- | --- | --- | --- | --- |
| Year | Study | Cancer | Adj | Time | Sample | Negative | Positive | Sex (female/male) | N of event | N of event | HR | HR_LL | HR_UL | HR | HR_LL | HR_UL | HR | HR_LL | HR_UL | HR | HR_LL | HR_UL |
| 2021 | Taieb, J | CRC | —— | 1 | 1017 | 877 | 140 | 441/576 | 1017 (298) | 1017 (184) | —— | —— | —— | 1.55 | 1.13 | 2.12 | 1.65 | 1.12 | 2.43 | —— | —— | —— |
| 2021 | Benhaim, L. | CRC | —— | 1 | 171 | 153 | 18 | 73/98 | 171 (29) | —— | —— | —— | —— | —— | —— | —— | —— | —— | —— | 3.22 | 1.32 | 7.89 |
| 2022 | Li,Y | CRC | —— | 1 | 151 | 127 | 24 | ——/—— | 151 (39) | —— | 5.538 | 2.487 | 12.331 | —— | —— | —— | —— | —— | —— | —— | —— | —— |
|  |  | CRC | yes | 3 | 124 | 113 | 11 | ——/—— | 124 (36) | —— | 3.272 | 1.017 | 9.992 | —— | —— | —— | —— | —— | —— | —— | —— | —— |
| 2021 | Loupakis, F | CRC | yes | 1 | 112 | 51 | 61 | 40/72 | 112 (82) | —— | —— | —— | —— | 5.78 | 3.34 | 10 | —— | —— | —— | —— | —— | —— |
| 2019 | Tarazona, N | CRC | —— | 1 | 69 | 55 | 14 | ——/—— | 69 (18) | —— | —— | —— | —— | 11.64 | 3.67 | 36.88 | —— | —— | —— | —— | —— | —— |
|  |  | CRC | —— | 2 | 94 | 62 | 32 | 33/61 | 94 (18) | —— | —— | —— | —— | 11.12 | 2.53 | 55.65 | —— | —— | —— | —— | —— | —— |
| 2022 | Henriksen, T. V | CRC | —— | 1 | 140 | 120 | 20 | 65/95 | 140 (38) | —— | 30.97 | 10.63 | 90.2 | —— | —— | —— | —— | —— | —— | —— | —— | —— |
|  |  | CRC | —— | 2 | 114 | 92 | 22 | ——/—— | 114 (24) | —— | 40.7 | 11.6 | 143 | —— | —— | —— | —— | —— | —— | —— | —— | —— |
|  |  | CRC | —— | 3 | 93 | 83 | 10 | ——/—— | 93 (20) | —— | 94.25 | 15.74 | 564.3 | —— | —— | —— | —— | —— | —— | —— | —— | —— |
| 2022 | McNamara, S | CRC | —— | 1 | 84 | 76 | 8 | ——/—— | 84 (9) | —— | —— | —— | —— | —— | —— | —— | —— | —— | —— | 7.098 | 2.0244 | 24.8871 |
| 2019 | Reinert, T | CRC | —— | 1 | 94 | 84 | 10 | ——/—— | 94 (17) | —— | 4.5 | 1.6 | 12.8 | —— | —— | —— | —— | —— | —— | —— | —— | —— |
|  |  | CRC | yes | 3 | 58 | 51 | 7 | 13/45 | 58 (14) | —— | 11.8 | 3.4 | 40.8 | —— | —— | —— | —— | —— | —— | —— | —— | —— |
|  |  | CRC | —— | 2 | 75 | 60 | 15 | ——/—— | 75 (16) | —— | 39.9 | 7.5 | 211 | —— | —— | —— | —— | —— | —— | —— | —— | —— |
| 2021 | Anandappa, Gayathri | CRC | —— | 1 | 64 | 53 | 11 | ——/—— | 64 (14) | —— | 28.8 | 3.5 | 234.1 | —— | —— | —— | —— | —— | —— | —— | —— | —— |
| 2023 | Kotani, D | CRC | —— | 1 | 1039 | 852 | 187 | 489/550 | 1039 (196) | —— | 10.82 | 7.07 | 16.6 | —— | —— | —— | —— | —— | —— | —— | —— | —— |
| 2021 | Chen, G | CRC | —— | 1 | 240 | 220 | 20 | 106/134 | 240 (23) | —— | 8.02 | 3.59 | 17.92 | —— | —— | —— | —— | —— | —— | —— | —— | —— |
|  |  | CRC | yes | 3 | 137 | 125 | 12 | ——/—— | 137 (——) | —— | 20.79 | 6.66 | 64.87 | —— | —— | —— | —— | —— | —— | —— | —— | —— |
|  |  | CRC | —— | 2 | 125 | 100 | 25 | ——/—— | 125 (——) | —— | 23 | 6.3 | 69.97 | —— | —— | —— | —— | —— | —— | —— | —— | —— |
| 2019 | Tie, J | CRC | all | 1 | 159 | 140 | 19 | 52/107 | 159 (23) | —— | 6 | 2.2 | 15 | —— | —— | —— | —— | —— | —— | —— | —— | —— |
| 2016 | Tie, J | CRC | no | 1 | 178 | 164 | 14 | ——/—— | 178 (27) | —— | 28 | 11 | 68 | —— | —— | —— | —— | —— | —— | —— | —— | —— |
|  |  | CRC | all | 1 | 230 | 210 | 20 | 99/131 | 230 (——) | —— | 14 | 6.8 | 28 | —— | —— | —— | —— | —— | —— | —— | —— | —— |
| 2023 | Watanabe, Jun | CRC | —— | 1 | 2083 | 1797 | 286 | ——/—— | 2083 (——) | —— | —— | —— | —— | 11.68 | 8.61 | 15.85 | —— | —— | —— | —— | —— | —— |
| 2023 | Mo, S. | CRC | —— | 1 | 255 | 196 | 59 | ——/—— | 255 (50) | —— | 17 | 8.2 | 34 | —— | —— | —— | —— | —— | —— | —— | —— | —— |
| 2022 | Wang, S | NSCLC | —— | 1 | 116 | 100 | 16 | ——/—— | 116 (——) | —— | 5.49 | 1.8642 | 16.1675 | —— | —— | —— | —— | —— | —— | —— | —— | —— |
|  |  | NSCLC | —— | 2 | 117 | 77 | 40 | ——/—— | 117 (34) | —— | 8.33 | 3.59 | 19.3 | —— | —— | —— | —— | —— | —— | —— | —— | —— |
|  |  | NSCLC | —— | 1 | 114 | 98 | 16 | ——/—— | 114 (——) | —— | 4.17 | 1.8 | 9.7 | —— | —— | —— | —— | —— | —— | —— | —— | —— |
|  |  | NSCLC | —— | 1 | 89 | 78 | 11 | ——/—— | 89 (——) | —— | 4.59 | 1.681 | 12.5 | —— | —— | —— | —— | —— | —— | —— | —— | —— |
| 2021 | Qiu, B | NSCLC | —— | 1 | 85 | 67 | 18 | ——/—— | 85 (33) | —— | 3.5 | 1.7 | 7.4 | —— | —— | —— | —— | —— | —— | —— | —— | —— |
|  |  | NSCLC | yes | 3 | 64 | 56 | 8 | ——/—— | 64 (——) | —— | 4.4 | 1.6 | 12 | —— | —— | —— | —— | —— | —— | —— | —— | —— |
|  |  | NSCLC | —— | 2 | 89 | 54 | 35 | ——/—— | 89 (34) | —— | 6.7 | 2.8 | 16 | —— | —— | —— | —— | —— | —— | —— | —— | —— |
| *2023 | Chen, K | NSCLC | —— | 1 | 156 | 137 | 19 | ——/—— | 156 (——) | —— | —— | —— | —— | 8.86 | 3.72 | 21.1 | —— | —— | —— | —— | —— | —— |
|  |  | NSCLC | —— | 2 | 110 | 87 | 23 | ——/—— | 110 (19) | —— | —— | —— | —— | 47.2 | 11.274 | 197.6 | —— | —— | —— | —— | —— | —— |
| 2022 | Xia, L | NSCLC | —— | 1 | 329 | 303 | 26 | ——/—— | 329 (70) | —— | 8.6 | 4.8 | 15.3 | —— | —— | —— | —— | —— | —— | —— | —— | —— |
| 2022 | Zhang, J. T. | NSCLC | —— | 2 | 236 | 190 | 46 | ——/—— | 236 (47) | —— | —— | —— | —— | 34.037 | 13.8681 | 83.5385 | —— | —— | —— | —— | —— | —— |
| 2023 | Yuan, Shu-Qiang | GC | —— | 1 | 100 | 75 | 25 | 34/68 | 100 (33) | 100 (——) | 2.48 | 1.24 | 4.97 | —— | —— | —— | 2.05 | 0.93 | 4.55 | —— | —— | —— |
|  |  |  |  | 3 | 41 | 31 | 10 | 15/26 | 41 (24) | 41 (——) | 10.31 | 2.04 | 52.03 | —— | —— | —— | 8.11 | 1.55 | 42.27 | —— | —— | —— |
| 2020 | Fedyanin, M. | GC | —— | 1 | 42 | 32 | 10 | ——/—— | 42 (12) | —— | —— | —— | —— | 6.6 | 1.5 | 30 | —— | —— | —— | —— | —— | —— |
| 2020 | Yang, J. | GC | —— | 1 | 38 | 31 | 7 | ——/—— | 38 (17) | 38 (——) | —— | —— | —— | 6.135 | 1.74 | 21.637 | 3.446 | 0.855 | 13.893 | —— | —— | —— |
|  |  | GC | —— | 2 | 44 | 27 | 17 | ——/—— | 44 (36) | 44 (13) | —— | —— | —— | 14.927 | 3.397 | 65.59 | 4.073 | 0.959 | 17.299 | —— | —— | —— |
|  |  | GC | —— | 3 | 23 | 18 | 5 | ——/—— | 23 (——) | 23 (——) | —— | —— | —— | 4.733 | 0.719 | 31.158 | 12.458 | 0.596 | 260.327 | —— | —— | —— |
| 2022 | Hata, Tatsuo | PAAD | —— | 1 | 66 | 50 | 16 | 21/45 | 66 (——) | 66 (——) | —— | —— | —— | 2.667 | 1.259 | 5.693 | 2.125 | 0.83 | 5.445 | —— | —— | —— |
| 2020 | Jiang, J. | PAAD | —— | 1 | 27 | 18 | 9 | 10/17 | 27 (14) | —— | —— | —— | —— | 3.6 | 1.15 | 11.28 | —— | —— | —— | —— | —— | —— |
| 2019 | Lee, B | PAAD | —— | 1 | 35 | 22 | 13 | 14/21 | 35 (23) | 35 (——) | 6.3 | 2.4 | 16.2 | —— | —— | —— | 7.5 | 2.1 | 27.7 | —— | —— | —— |
| 2022 | Yamaguchi, T | PAAD | —— | 1 | 97 | 70 | 27 | 54/43 | 97 (——) | 97 (——) | 1.61 | 0.9 | 2.77 | —— | —— | —— | 1.36 | 0.75 | 2.39 | —— | —— | —— |
| 2021 | Wang, D. S | CRLM | —— | 3 | 49 | 27 | 22 | 19/30 | 49 (12) | —— | 2.398 | 1.1161 | 5.1523 | —— | —— | —— | —— | —— | —— | —— | —— | —— |
| 2023 | Liu, W | CRLM | —— | 1 | 134 | 92 | 42 | 36/98 | 134 (84) | —— | —— | —— | —— | 1.89 | 1.14 | 3.11 | —— | —— | —— | —— | —— | —— |
| 2022 | Nishioka, Y. | CRLM | —— | 1 | 105 | 73 | 32 | 42/63 | 105 (66) | —— | 2.04 | 1.18 | 3.52 | —— | —— | —— | —— | —— | —— | —— | —— | —— |
| 2021 | Bolhuis, K. | CRLM | yes | 1 | 23 | 17 | 6 | 8/15 | 23 (17) | —— | 4.1 | 1.19 | 14.47 | —— | —— | —— | —— | —— | —— | —— | —— | —— |
| 2021 | Tie, J | CRLM | —— | 1 | 49 | 37 | 12 | 14/35 | 49 (21) | —— | 3.13 | 1 | 9.82 | —— | —— | —— | —— | —— | —— | —— | —— | —— |
| 2022 | Reinert, T | CRLM | —— | 1 | 40 | 27 | 13 | ——/—— | 40 (28) | —— | 10.8 | 3.6 | 32.7 | —— | —— | —— | —— | —— | —— | —— | —— | —— |
|  |  | CRLM | —— | 2 | 67 | 33 | 34 | ——/—— | 67 (47) | —— | 4.9 | 2.5 | 10 | —— | —— | —— | —— | —— | —— | —— | —— | —— |
| 2022 | Schneider, B. P. | BC | —— | 1 | 146 | 53 | 93 | ——/—— | 146 (——) | 146 (——) | —— | —— | —— | 1.93 | 1.05 | 3.52 | 2.64 | 1.18 | 5.91 | —— | —— | —— |
| 2017 | Chen, Y. H. | BC | —— | 1 | 33 | 29 | 4 | ——/—— | 33 (13) | —— | —— | —— | —— | 8.6 | 1.6 | 45.7 | —— | —— | —— | —— | —— | —— |
| 2015 | Garcia-Murillas, Isaac | BC | —— | 1 | 37 | 30 | 7 | 37/0 | 37 (12) | —— | —— | —— | —— | 21.1 | 2.5 | 177.47 | —— | —— | —— | —— | —— | —— |
|  |  | BC | —— | 2 | 43 | 30 | 13 | 43/0 | 43 (15) | —— | —— | —— | —— | 9.6 | 2.4 | 38.9 | —— | —— | —— | —— | —— | —— |
| 2021 | Liu, T. | ESCA | —— | 1 | 23 | 19 | 4 | ——/—— | 23 (5) | 23 (5) | —— | —— | —— | 184.6 | 3.6 | 9576.9 | 25.8 | 2.7 | 242.6 | —— | —— | —— |
| 2021 | Ococks, E. | ESCA | —— | 2 | 63 | 53 | 10 | ——/—— | 63 (26) | —— | —— | —— | —— | 4.77 | 1.93 | 11.8 | —— | —— | —— | —— | —— | —— |
| 2022 | Chao, A. | OV | —— | 1 | 29 | 18 | 11 | ——/—— | 29 (8) | 29 (——) | —— | —— | —— | —— | —— | —— | 6.56 | 1.07 | 40.17 | 8.41 | 2.49 | 28.39 |
| 2022 | Carrasco, R | BLCA | —— | 1 | 34 | 22 | 12 | ——/—— | 34 (16) | —— | —— | —— | —— | —— | —— | —— | —— | —— | —— | 4.199 | 1.0827 | 16.2849 |
| 2021 | Powles, T | BLCA | no | 1 | 281 | 183 | 98 | 62/221 | 281 (——) | —— | —— | —— | —— | 6.19 | 4.29 | 8.91 | —— | —— | —— | —— | —— | —— |
| 2019 | Christensen, Emil | BLCA | —— | 2 | 64 | 47 | 17 | ——/—— | 64 (13) | —— | 129.6 | 12.6 | 17871.1 | —— | —— | —— | —— | —— | —— | —— | —— | —— |
| 2019 | Tan, L. | melanoma | —— | 1 | 52 | 39 | 13 | ——/—— | 52 (29) | —— | 11 | 3.7 | 31.5 | —— | —— | —— | —— | —— | —— | —— | —— | —— |
| 2022 | Zhao, L | HCC | —— | 2 | 59 | 38 | 21 | ——/—— | 59 (27) | —— | 8.5 | 2.91 | 24.86 | —— | —— | —— | —— | —— | —— | —— | —— | —— |

1=landmark detection; 2=longitudinal detection; 3=post-adjuvant therapy; Adj= Adjuvant therapy.
