# Supplementary material for: Circulating tumour DNA-Based molecular residual disease detection in resectable cancers: a systematic review and meta-analysis
Source: eBioMedicine. 2024 Apr 13;103:105109. doi: 10.1016/j.ebiom.2024.105109 (PMC11021841; doi:10.1016/j.ebiom.2024.105109)
Supplement: Table S5 [file mmc5.docx]

Table S5 Included in the article feature list

| Author | Year | ctDNA detection time | ctDNA detection technology | Country | Mid-follow-up | | Magazine | Report type | Age（year） | Sex (female/male) | Tumor type | Tumor stage | Therapy |
| --- | --- | --- | --- | --- | --- | --- | --- | --- | --- | --- | --- | --- | --- |
| Taieb, J,et al | 2021 | postoperation-42d | ddPCR | France | 79.2m | | clinical cancer research | Random | 63.9 (9.4) | 441/576 | CRC | Ⅲ | ACT |
| Tie, J,et al | 2022 | postoperation-4w/7w | mPCR-NGS（Safe-SeqS） | Australia | 37 m | | The new england journal of medicine | Random | 64（28-94） | —— | CRC | II | ACT |
| Benhaim, L,et al | 2021 | postoperation-5d | ddPCR | England | 36~60m | | Eur J Cancer | Prospective | 66.7 (30.1-86.1) | 73/98 | CRC | II/III | ACT |
| Li, Y,et al | 2022 | postoperation-30d | hybridization capture-based NGS | China | 33.5m | | Eur J Cancer | Prospective | 61（25-86） | —— | CRC | III | Chemotherapy/ targeted therapy |
|  |  | post-ACT-3~6m |  |  |  | |  |  |  | —— |  |  |  |
| Loupakis, F,et al | 2021 | postoperation-27d | mPCR-NGS | Italy | 10.7m (range: 0.9-53.8m） | | BIOMARKERS | Prospective | 60.1 | 40/72 | CRC | Ⅳ | ACT |
|  |  | not adjuvant after surgery |  |  |  | |  |  |  | —— |  |  |  |
| Tarazona, N,et al | 2019 | postoperation-6-8w | hybridization capture-based NGS | Spain | 7.1m | | ORIGINAL ARTICLE | Prospective | 71 (41-93) | —— | CRC | I-III | ACT |
|  |  | longitudinal-4m |  |  |  | |  |  |  | 33/61 |  |  |  |
| Henriksen, T. V,et al | 2022 | postoperation-2w | mPCR-NGS | Denmark and Spain | 35m | | CLINICAL CANCER RESEARCH | Prospective | <70-93 (58.1%)；>=70-67 (41.9%) | —— | CRC | III |  |
|  |  | longitudinal |  |  |  | |  |  |  | —— |  |  |  |
|  |  | post-adjuvant |  |  |  | |  |  |  | —— |  |  | ACT |
| McNamara, Sylvie,et al | 2022 | postoperation-7d(1~34d) | hybridization capture-based NGS | —— | —— | | Journal of Clinical Oncology | Prospective | —— | —— | CRC | II/III | adjuvant therapy |
| Reinert, T,et al | 2019 | postoperation-30d | mPCR-NGS | Denmark | 12.5m | | JAMA oncology | Prospective | 67.9 | —— | CRC | I-III | ACT |
|  |  | post-adjuvant |  |  |  | |  |  |  | 13/45 |  |  |  |
|  |  | longitudinal-3m |  |  |  | |  |  |  | —— |  |  |  |
| Bryant Chee,et al | 2021 | postoperation-3w | Guardant Reveal, Guardant Health | America | 12.5m (rang: 1-48m) | | J Clin Oncol | Prospective | —— | —— | CRC | —— | adjuvant therapy |
| Han, S. W,et al | 2022 | postoperation-3w | hybridization capture-based NGS | Korea | —— | | Annals of Oncology | Prospective | —— | —— | CRC | II/III | adjuvant therapy |
| Anandappa, G,et al | 2021 | postoperation | mPCR-NGS(Signatera) | England | 15.48m (range: 0.16 - 42.1m) | | J Clin Oncol | Prospective | —— | —— | CRC | III | adjuvant therapy |
| Kotani, D,et al | 2023 | postoperation-4w | mPCR-NGS | Japan | 16.74m | | Nat Med | Prospective | 68（25-88） | 489/550 | CRC | II/III | ACT |
| Hofste, L,et al | 2023 | postoperation1-2w | hybridization capture-based NGS | Australia | 13m (range: 1-50m) | | European Journal of Surgical Oncology | Prospective | 66 (48-84) | —— | CRC | —— | ACT |
| Chen, G,et al | 2021 | postoperation-3-7d | hybridization capture-based NGS | China | 27.4m | | journal of hematology&oncology | Prospective | 60（19-84） | 106/134 | CRC | II/III | ACT |
|  |  | post-adjuvant |  |  |  | |  |  |  | —— |  |  |  |
|  |  | longitudinal-6m-3m |  |  |  | |  |  |  | —— |  |  |  |
| Tie, J,et al | 2019 | postoperation-4-10w | mPCR-NGS(Safe-SeqS) | Australia | 24m | | GI cancer | Prospective | 62（28-86） | 52/107 | CRC | III | ACT |
|  |  | no adjuvant after surgery |  |  |  | |  |  |  | —— |  |  |  |
|  |  | adjuvant after surgery |  |  |  | |  |  |  | —— |  |  |  |
| Tie, J,et al | 2016 | postoperation-4-10w | mPCR-NGS（Safe-SeqS） | Australia | 27m (range: 2 -52m) | | Sci Transl Med | Prospective | 65 (59–73) | 99/131 | CRC | II | —— |
|  |  | no adjuvant after surgery |  |  |  | |  |  |  | —— |  |  |  |
|  |  | adjuvant after surgery |  |  |  | |  |  |  | —— |  |  |  |
| Watanabe, Jun,et al | 2023 | postoperation-1m | mPCR-NGS(Signatera) | Japan | 16.3m | | JCO Global Oncology | Prospective | —— | —— | CRC | II-IV | adjuvant therapy |
| Zhou, Jian,et al | 2022 | postoperation-8d | hybridization capture-based NGS (PROPHET) | China | —— | | Cancer Research | Prospective | —— | —— | CRC | I-III | adjuvant therapy |
| Mo, S,et al | 2023 | postoperation-1m | Circulating tumour DNA methylation | China | 21m (range: 8-27m) | | JAMA Oncol | Prospective | 60.1(10.3) | —— | CRC | I-III | ACT |
|  |  | longitudinal/3m |  |  |  | |  |  |  | —— |  |  |  |
|  |  | post-adjuvant |  |  |  | |  |  |  | —— |  |  |  |
| Li, N,et al | 2022 | postoperation-1m | hybridization capture-based NGS | China | 30.7m (range: 28.8-32.6 m) | | Cancer | Prospective | 62（35-78） | —— | NSCLC | I-IIIA | ACT |
|  |  | longitudinal/3-6m |  |  |  | |  |  |  | 49/70 |  |  |  |
| Peng, M,et al | 2020 | postoperation | cSMART assay | China | 46m | | Front Oncol | Prospective | 60.3 (40-78) | 20/51 | NSCLC | I-IV | ACT |
| Kuang, P. P,et al | 2020 | postoperation-2w | hybridization capture-based NGS | China | 15.8m (range: 3.7 ~ 36.7m) | | Front Oncol | Prospective | 57.4 | —— | NSCLC | IB-III | ACT |
|  |  | post-adjuvant |  |  |  | |  |  |  | —— |  |  |  |
| Wang, S,et al | 2022 | postoperation-7d | hybridization capture-based NGS | China | 30m | | journal of Hematology&Oncology | Prospective | 63（39-79） | —— | NSCLC | I-III | Chemotherapy/ targetedtherapy/radiotherapy |
|  |  | longitudinal/3m |  |  |  | |  |  |  | —— |  |  |  |
| Qiu, B,et al | 2021 | postoperation-30d | hybridization capture-based NGS | China | last time: 18m | | nature communication | Prospective | 64 (38–82) | —— | NSCLC | I-IV | Chemotherapy/ targetedtherapy/radiotherapy |
|  |  | post-adjuvant-4m |  |  |  | |  |  |  | —— |  |  |  |
|  |  | longitudinal-3m |  |  |  | |  |  |  | —— |  |  |  |
| Chen, K,et al | 2023 | postoperation3d-1m | Circulating tumour DNA methylation | China | 25m | | BMC Med | Prospective | 62.5 (39–78 | —— | NSCLC | I-IV | Chemotherapy/ targetedtherapy/radiotherapy/Immunotherapy |
|  |  | longitudinal |  |  |  | |  | Prospective |  | —— |  |  |  |
| Fu, R,et al | 2023 | postoperation-1m（+-7d） | hybridization capture-based NGS | China | 16.0m | | Mol Oncol | Prospective | 60（30-82） | —— | NSCLC | I-III | ACT |
|  |  | longitudinal-3~6m |  |  |  | |  |  |  | 81/96 |  |  |  |
| Yang, W,et al | 2020 | longitudinal-7d-3m/1y-6m/2~3y | hybridization capture-based NGS | China | 22.83m (range: 7.97-45.13m） | | Lung Cancer | Prospective | 55.8 | 49/33 | NSCLC | I | adjuvant therapy |
| Tan, A,et al | 2021 | longitudinal | mPCR-NGS(SignateraTM) | Singapore | 33.0m (range: 9.8-72.1m) | | Journal of Thoracic Oncology | Prospective | 60(43-83) | 23/34 | NSCLC | I-III | adjuvant therapy |
| Chen, K,et al | 2019 | postoperation-3d | cSMART | China | 17.7m (range: 16-22.7m) | | Clinical Cancer Research | Prospective | 63 | —— | NSCLC | I–III | Chemotherapy/ targetedtherapy/radiotherapy |
| Chen, K,et al | *2023 | postoperation-(1m) | hybridization capture-based NGS (PROPHET) | China | 35.7m (range: 32.9-37.9m) | | Cancer Cell | Prospective | 62.7 | —— | NSCLC | I–III | ACT |
|  |  | longitudinal |  |  |  | |  |  |  | 56/54 |  |  |  |
| Waldeck, S,et al | 2022 | postoperation-1-2w | hybridization capture-based NGS | Germany | 26.2m | | Mol Oncol | Prospective | 70（48-85） | —— | NSCLC | I–III | ACT |
| Xia, L,et al | 2022 | postoperation3d-1m | hybridization capture-based NGS | China | 35.6m (range: 11.4-44.7m) | | CLINICAL CANCER RESEARCH | Prospective | 59(28-80) | —— | NSCLC | I-III | adjuvant therapy |
| Zhang, J. T,et al | 2022 | postoperation-1m(±7d) | hybridization capture-based NGS | China | 19.7m | | Cancer Discov | Prospective | 62(27- 84) | —— | NSCLC | I-III | adjuvant therapy |
|  |  | longitudinal/3 ~ 6m |  |  |  | |  |  |  | —— |  |  |  |
| Yuan, Shu-Qiang,et al | 2023 | postoperation-4d (1~7d) | hybridization capture-based NGS | China | 52.2m (range: 50.5-54.3m) | | cancer communications | Prospective | <65 years 70 (70.0) ≥65 years 30 (30.0) | 34/68 | GC | II/III | ACT |
|  |  | post-adjuvant |  |  |  | |  |  |  | 15/26 |  |  |  |
| Leal, A,et al | 2020 | postoperation-6.5w | hybridization capture-based NGS | Netherland,Denmark， Sweden | 42m | | Nat Commun | Random | 63.5(30-77) | —— | GC | I-IV | Neoadjuvant chemotherapy |
| Fedyanin, M,et al | 2020 | postoperation-7d(5-15d) | ddPCR | Russia | —— | | Annals of Oncology | Prospective | —— | —— | GC | I-IV | ACT |
| Yang, J,et al | 2020 | postoperation-1m | hybridization capture-based NGS | China | 7.2m | | Cell Death Dis | Prospective | 54（28-78） | —— | GC | I-III | adjuvant therapy |
|  |  | longitudinal-3m/1y-6m |  |  |  | |  |  |  | —— |  |  |  |
|  |  | post-adjuvant |  |  |  | |  |  |  | —— |  |  |  |
| Xue, Pei,et al | 2023 | postoperation2 - 4w | hybridization capture-based NGS | China | 26.1m (range: 19.4-28.4m) | | Cancer Research | Prospective | 65(35-87) | —— | GC | I-III | adjuvant therapy |
| Hata, T,et al | 2022 | postoperation-2w | ddPCR | Japan | 26.1m (range: 19.3-42.6 m) | | Journal of Hepato-Biliary-Pancreatic Sciences | Prospective | 68(39–89) | 21/45 | PAAD | —— | ACT |
| Popova, A,et al | 2020 | postoperation-7d(5-9d) | dPCR | Russia | —— | | Annals of Oncology |  | —— | —— | PAAD | —— | adjuvant therapy |
| Jiang, J,et al | 2020 | postoperation-7d | hybridization capture-based NGS | China | 18.6m (range: 12.4-28.9m) | | Front Oncol | Prospective | 62 (43–82) | 10/17 | PAAD | I/Ⅱ/Ⅳ | adjuvant therapy |
| Lee, B,et al | 2019 | postoperation4-8w | mPCR-NGS(SignateraTM) | Australia | 38.4m | | ORIGINAL ARTICLE | Prospective | 66（47-79） | 14/21 | PAAD | —— | ACT |
| Wang, Xiuchao,et al | 2023 | postoperation-7d | hybridization capture-based NGS (brPROPHET) | China | 6.3m (range: 5.3-13.2m) | | Journal of Clinical Oncology | Prospective | —— | —— | PAAD | I-III | adjuvant therapy |
| Kitahata, Y | 2022 | postoperation-4–8w | ddPCR | Japan | 14.5m (range: 9.47–38.4m) | | Ann Surg Oncol | Prospective | 69 (57–80) | 7(53.8) | PAAD | —— | ACT |
| Yamaguchi, T | 2021 | postoperation-3d | ddPCR | Japan | 29m (range: 2–132 m) | | Ann Surg Oncol | Prospective | 69 (43–88). | 54/43 | PAAD | —— | ACT |
| Wang, D. S,et al | 2021 | postoperation-38d | hybridization capture-based NGS | China | 17.4m (range: 6.6-28.7m） | | Theranostics | Prospective | 56 (20 - 75) | 29/53 | CRLM | Ⅳ | —— |
|  |  | post-adjuvant-8d |  |  |  | |  |  | 56 (20 - 73) | 19/30 |  |  | ACT |
| Liu, W,et al | 2023 | postoperation-31d | hybridization capture-based NGS | China | 17.3m (range: 3.3–37.3m) | | Ann Surg Oncol | Prospective | 58 (28–79) | 36/98 | CRLM | —— | ACT |
| Nishioka, Y,et al | 2022 | postoperation-pre-adj | Guardant Reveal | America | 28.3m (range:6.0 - 99.8m） | | J Am Coll Surg | Prospective | 54 (46–62) | 42/63 | CRLM | —— | ACT |
| Michael J,et al | 2017 | postoperation-17d | Guardant Reveal, Guardant Health | America | 33m | | J Clin Oncol | Prospective | —— | —— | CRLM | —— | ACT |
| Newhook, T. E,et al | 2022 | postoperation-18d( 13-68d) | Guardant Reveal, Guardant Health | America | 55 m | | Ann Surg | Prospective | 56.7 (48.7-64.0) | —— | CRLM | —— | ACT |
| Bolhuis, K,et al | 2021 | postoperation-3m | ddPCR | Netherland | 19.6m | | EBioMedicine | Random | 63 (54-76) | 8/15 | CRLM | —— | ACT |
| Jiang, H,et al | 2023 | postoperation-28d | hybridization capture-based NGS | China | 9.67m | | Front Oncol | Prospective | ≥ 65y，20，<65y，47 | 29/38 | CRLM | —— | targeted therapy |
| Schøler, L. V,et al | 2017 | postoperation-3m | ddPCR | Denmark | last time: 36m | | Clinical Cancer Research | Prospective | —— | 6/15 | CRLM | I–III | Chemotherapy/ targetedtherapy |
| Tie, J,et al | 2021 | postoperation4-10w | mPCR-NGS(SignateraTM) | Australia | 50.5m (range, 5 to 82 m) | | plos medicine | Prospective | 62.8（30.8-84.7） | 14/35 | CRLM | Ⅳ | ACT |
|  |  | post-adjuvant |  |  |  | |  |  |  | —— |  |  |  |
| Reinert, T,et al | 2022 | postoperation-30d | ddPCR | England | 19.5m | | Iternational journal of cancer | Prospective | 67.7 (45.1-89.4) | —— | CRLM | —— | ACT |
|  |  | longitudinal/3m |  |  |  | |  |  |  | —— |  |  |  |
| Schneider, B. P,et al | 2022 | postoperation-14d | hybridization capture-based NGS | America | 34.2m | | Journal of clinical oncology | Random | 50 | —— | BC | —— | adjuvant therapy |
| Sharma, P,et al | 2022 | postoperation | hybridization capture-based NGS | America | —— | | Cancer Research | Prospective | —— | —— | BC | —— | Chemotherapy/radiotherapy |
| Coombes, R. C,et al | 2019 | postoperation | mPCR-NGS | England | last time: 100m | | Clin Cancer Res | Prospective | 57 (38–81) | —— | BC | I-III | ACT |
|  |  | longitudinal |  |  |  | |  |  |  | —— |  |  |  |
| Chen, Y. H,et al | 2017 | postoperation | mPCR-NGS | India | 4.6-8.9m | | NPJ Breast Cancer | Prospective | 47（21-75） | —— | BC | —— | ACT |
| Zhou, Y,et al | 2021 | postoperation-3~7d | hybridization capture-based NGS | China | 23.2m (range: 16-26.3m） | | Breast Cancer Research and Treatment | Prospective | 47 (27–67) | —— | BC | IIb-Ⅳ | Chemotherapy/radiotherapy |
| Garcia-Murillas, Isaac,et al | 2015 | postoperation2 - 4w | dPCR | England | 12m | | CANCER | Prospective | —— | 37/0 | BC | —— | adjuvant therapy |
|  |  | longitudinal/6m |  |  |  | |  |  |  | 43/0 |  |  |  |
| Openshaw, M. R,et al | 2020 | postoperation | ddPCR | England | 31.1m | | Br J Cancer | Prospective | 68(47-90) | —— | ESCA | —— | ACT |
|  |  | longitudinal |  |  |  | |  |  |  | —— |  |  |  |
| Takei, Shogo,et al | 2023 | postoperation-4w | mPCR-NGS（Signatera） | Japan | —— | | Journal of Clinical Oncology | Prospective | —— | —— | ESCA | —— | Neoadjuvant chemotherapy |
| Morimoto, Y,et al | 2023 | postoperation1/3m | mPCR-NGS | Japan | last time: 60m | | Ann Surg Oncol | Prospective | 68 (47–79) | 3/13 | ESCA | I-IV | Neoadjuvant chemotherapy |
| Gerlinger, Marco,et al | 2023 | postoperation-pre-ACT | mPCR-NGS（Signatera） | England | 17.0m | | Cancer Research | Prospective | —— | —— | ESCA | —— | ACT |
| Liu, T,et al | 2021 | postoperation-1w | hybridization capture-based NGS | China | 34.8m (range:1.5 ~ 50.67m) | | Front Oncol | Prospective | 65(46 ~ 79) | —— | ESCA | II/III | adjuvant therapy |
| Ococks, E,et al | 2021 | longitudinal | hybridization capture-based NGS | England | 32.9m | | Ann Oncol | Prospective | 68 | —— | ESCA | —— | ACT |
| Ananda, Sumitra,et al | 2023 | postoperation | mPCR-NGS(SaferSeqS) | Australia | —— | | Journal of Clinical Oncology | Prospective | —— | —— | OV | I-IV | ACT |
| Chao, A,et al | 2022 | postoperation7-10d | mPCR-NGS | China | 33.15m (range: 0.79-46.13m) | | Biomed J | Prospective | —— | —— | OV | I-IV | ACT |
| Chapman, J, S,et al | 2021 | postoperation | mPCR-NGS | America | 15m (range:0.6-26m) | | Cancer Research | Prospective | —— | —— | OV | I-IV | adjuvant therapy |
| Carrasco, R,et al | 2022 | postoperation-4m | hybridization capture-based NGS | Spain | 36m | | Int J Mol Sci | Prospective | 71(51-85) | —— | BLCA | —— | ACT |
| Powles, T,et al | 2021 | postoperation-10w | mPCR-NGS(Signatera) | England | 21.9m (range:16-45m） | | Nature | Random | —— | 62/221 | BLCA | —— | —— |
| Powles, T,et al | 2023 | postoperation-10w | mPCR-NGS(Signatera) | England | 46.8m(range:36.1-53.6m） | European Urology | | Random | —— | 62/221 | BLCA | —— | —— |
| Szabados, B,et al | 2022 | postoperation | mPCR-NGS | England | 25m (range:25 - 26m) | | European urology | Prospective | 73 (54–85) | —— | BLCA | —— | Neoadjuvant chemotherapy |
| Christensen, Emil,et al | 2019 | longitudinal | hybridization capture-based NGS | Denmark | 21m | | original report | Prospective | 65.2（43-79） | —— | BLCA | —— | Neoadjuvant chemotherapy |
| Tan, L,et al | 2019 | postoperation-2w | ddPCR | Australia | 18m | | Annals of Oncology | Prospective | 57(22-93) | —— | melanoma | III | immunotherapy |
| Genta, Sofia,et al | 2022 | postoperation | mPCR-NGS | Canada | —— | | ASCO | Prospective | 66(27 ~ 87) | 12/33 | melanoma | II-IV | adjuvant therapy |
| Eroglu, Zeynep | 2023 | postoperation-pre-ACT | mPCR-NGS | America | 19.6m (range: 0.4–24.2m) | | Cancer | Prospective | 72 (21–90) | 9/20 | melanoma | Ⅲ-Ⅳ | ICI |
| Zhao, L,et al | 2022 | longitudinal | hybridization capture-based NGS | China | 26.1m | | Clinical and Translational Medicine | Prospective | 54（34-77） | —— | HCC | —— | ACT |

W=week; m=month; d=day; ACT: adjuvant chemotherapy;
