# Supplementary material for: Circulating tumour DNA-Based molecular residual disease detection in resectable cancers: a systematic review and meta-analysis
Source: eBioMedicine. 2024 Apr 13;103:105109. doi: 10.1016/j.ebiom.2024.105109 (PMC11021841; doi:10.1016/j.ebiom.2024.105109)
Supplement: Table S6 [file mmc6.docx]

Table S6 The data and main features of the article are included (sensitivity and specificity)

| Year | Study | Cancer | Time | Tech | Ratio | TP | FP | FN | TN |
| --- | --- | --- | --- | --- | --- | --- | --- | --- | --- |
| 2021 | Loupakis, F | CRC | 1 | A | 0~1 | 59 | 2 | 23 | 28 |
| 2021 | Bryant | CRC | 1 | D | 0~1 | 23 | 2 | 4 | 16 |
| 2021 | Anandappa, G | CRC | 1 | B | 1~5 | 5 | 6 | 9 | 44 |
| 2019 | Tarazona, N. | CRC | 1 | C | 1~5 | 8 | 6 | 10 | 45 |
| 2023 | Kotani, D | CRC | 1 | A | 1~5 | 115 | 72 | 81 | 771 |
| 2023 | Mo, S. | CRC | 1 | O | 1~5 | 39 | 20 | 11 | 185 |
| 2021 | Benhaim, L. | CRC | 1 | B | 5~9 | 8 | 10 | 21 | 132 |
| 2022 | Li,Y | CRC | 1 | C | 5~9 | 13 | 11 | 26 | 101 |
| 2022 | Tie, J | CRC | 1 | A | 5~9 | 8 | 37 | 15 | 231 |
| 2019 | Reinert, T | CRC | 1 | A | 5~9 | 7 | 3 | 10 | 74 |
| 2022 | Henriksen, T. V | CRC | 1 | A | 5~9 | 16 | 4 | 22 | 98 |
| 2019 | Tie, J | CRC | 1 | A | 5~9 | 11 | 8 | 12 | 128 |
| 2023 | Hofste, L | CRC | 1 | C | 5~9 | 3 | 0 | 2 | 14 |
| 2016 | Tie, J | CRC | 1 | A | 9~13 | 11 | 3 | 16 | 148 |
| 2019 | Tarazona, N. | CRC | 2 | C | 1~5 | 14 | 18 | 4 | 58 |
| 2021 | Chen, G | CRC | 2 | C | 1~5 | 19 | 6 | 4 | 96 |
| 2019 | Reinert, T | CRC | 2 | A | 1~5 | 14 | 1 | 2 | 58 |
| 2022 | Henriksen, T. V | CRC | 2 | A | 1~5 | 21 | 1 | 3 | 89 |
| 2021 | Qiu, B | NSCLC | 1 | C | 1~5 | 14 | 4 | 19 | 48 |
| 2020 | Kuang, P. P. | NSCLC | 1 | C | 1~5 | 4 | 4 | 5 | 22 |
| 2022 | Waldeck, S | NSCLC | 1 | C | 1~5 | 4 | 0 | 4 | 8 |
| 2023 | Fu, R | NSCLC | 1 | C | 1~5 | 22 | 14 | 16 | 94 |
| 2019 | Chen, K | NSCLC | 1 | E | 1~5 | 6 | 1 | 3 | 15 |
| 2022 | Xia, L | NSCLC | 1 | C | 9~13 | 21 | 5 | 49 | 254 |
| 2022 | Zhang, J. T. | NSCLC | 1 | C | 9~13 | 17 | 4 | 30 | 194 |
| 2020 | Peng, M | NSCLC | 2 | E | 1~5 | 19 | 11 | 12 | 29 |
| 2020 | Yang, W. | NSCLC | 2 | C | 1~5 | 3 | 12 | 2 | 65 |
| 2022 | Li, N | NSCLC | 2 | C | 1~5 | 17 | 20 | 11 | 71 |
| 2023 | Fu, R | NSCLC | 2 | C | 1~5 | 30 | 25 | 11 | 111 |
| 2022 | Wang, S | NSCLC | 2 | C | 1~5 | 25 | 15 | 9 | 61 |
| 2021 | Qiu, B | NSCLC | 2 | C | 1~5 | 27 | 8 | 7 | 47 |
| *2023 | Chen, K | NSCLC | 2 | C | 1~5 | 16 | 7 | 3 | 84 |
| 2022 | Zhang, J. T. | NSCLC | 2 | C | 1~5 | 41 | 5 | 6 | 184 |
| 2019 | Lee, B | PAAD | 1 | A | 1~5 | 13 | 0 | 10 | 12 |
| 2020 | Jiang, J | PAAD | 1 | C | 1~5 | 8 | 1 | 6 | 12 |
| 2020 | Popova, A. | PAAD | 1 | B | 1~5 | 7 | 7 | 3 | 20 |
| 2023 | Wang, Xiuchao | PAAD | 1 | A | 5~9 | 2 | 0 | 2 | 13 |
| 2015 | Garcia-M | BC | 1 | B | 1~5 | 6 | 1 | 6 | 24 |
| 2017 | Chen, Y. H. | BC | 1 | A | 5~9 | 4 | 0 | 9 | 20 |
| 2021 | Zhou, Y | BC | 1 | C | 5~9 | 5 | 0 | 2 | 25 |
| 2015 | Garcia-M | BC | 2 | B | 1~5 | 12 | 1 | 3 | 27 |
| 2019 | Coombes, R. C. | BC | 2 | A | 1~5 | 16 | 0 | 2 | 31 |
| 2023 | Jiang, H | CRLM | 1 | O | 1~5 | 12 | 3 | 29 | 23 |
| 2021 | Bolhuis, K. | CRLM | 1 | B | 1~5 | 6 | 0 | 11 | 6 |
| 2023 | Liu, W | CRLM | 1 | C | 1~5 | 37 | 5 | 47 | 45 |
| 2022 | Nishioka, Y. | CRLM | 1 | D | 1~5 | 30 | 2 | 36 | 37 |
| 2022 | Reinert, T | CRLM | 1 | B | 1~5 | 13 | 0 | 15 | 12 |
| 2021 | Tie, J | CRLM | 1 | A | 1~5 | 10 | 2 | 11 | 26 |
| 2021 | Wang, D. S | CRLM | 1 | C | 1~5 | 27 | 7 | 20 | 28 |
| 2017 | Schøler, L. V | CRLM | 1 | B | 1~5 | 6 | 0 | 4 | 11 |
| 2022 | Reinert, T | CRLM | 2 | B | 1~5 | 34 | 0 | 13 | 20 |
| 2023 | Xue, Pei | GC | 1 | C | 1~5 | 1 | 0 | 2 | 10 |
| 2020 | Yang, J. | GC | 1 | C | 1~5 | 7 | 0 | 10 | 21 |
| 2023 | Yuan, shu qiang | GC | 1 | C | 1~5 | 14 | 11 | 19 | 56 |
| 2020 | Fedyanin, M. | GC | 1 | B | 1~5 | 6 | 4 | 6 | 26 |
| 2020 | Leal, A. | GC | 1 | C | 1~5 | 6 | 3 | 0 | 11 |
| 2020 | Yang, J. | GC | 2 | C | 1~5 | 16 | 1 | 3 | 24 |
| 2019 | Tan, L. | melanoma | 1 | B | 0~1 | 13 | 0 | 16 | 23 |
| 2022 | Zhao, L | HCC | 2 | C | 1~5 | 19 | 2 | 8 | 30 |
| 2022 | Carrasco, R | BLCA | 1 | B | 1~5 | 9 | 3 | 7 | 15 |
| 2023 | Powel,T | BLCA | 1 | A | 1~5 | 82 | 16 | 61 | 122 |
| 2022 | Szabados, B. | BLCA | 1 | A | 5~9 | 5 | 0 | 1 | 30 |
| 2019 | Christensen, E | BLCA | 2 | A | 1~5 | 13 | 4 | 0 | 47 |
| 2020 | Openshaw, M. R. | ESCA | 1 | B | 1~5 | 5 | 1 | 8 | 8 |
| 2021 | Liu, T. | ESCA | 1 | C | 1~5 | 3 | 1 | 2 | 17 |
| 2023 | Morimoto, Y | ESCA | 1 | A | 1~5 | 6 | 0 | 1 | 9 |
| 2021 | Ococks, E. | ESCA | 2 | C | 1~5 | 9 | 1 | 17 | 36 |
| 2020 | Openshaw, M. R. | ESCA | 2 | B | 1~5 | 8 | 1 | 5 | 8 |
| 2022 | Chao, A. | OV | 1 | A | 1~5 | 6 | 5 | 2 | 16 |
| 2021 | Chapman, J | OV | 1 | A | 1~5 | 7 | 0 | 0 | 13 |

1=landmark detection; 2=longitudinal detection; 3=post-adjuvant therapy; The ratio hierarchy is (0~1), [1,5), [5,9), [9,13); Ratio=the number of patients with ctDNA- / the number of patients with ctDNA+ ; A=mPCR-NGS, B=ddPCR, C=hybridization capture-based NGS, D=Guardant Reveal, O=circulating tumour DNA methylation, E=cSMART. TP: True Positive; FP: False Positive; TN: True Negative; FN: False Negative
