# Supplementary material for: Circulating tumour DNA-Based molecular residual disease detection in resectable cancers: a systematic review and meta-analysis
Source: eBioMedicine. 2024 Apr 13;103:105109. doi: 10.1016/j.ebiom.2024.105109 (PMC11021841; doi:10.1016/j.ebiom.2024.105109)
Supplement: Table S7 [file mmc7.docx]

| Table S7 The data of definition of ctDNA, inclusion criteria and exclusion criteria of including studies. | | | | | | |  |  |  | |
| --- | --- | --- | --- | --- | --- | --- | --- | --- | --- | --- |
| Year | Study | Cancer | Time | Adjusted：recurrence | Adjusted：OS | Definition of ctDNA | Inclusion criteria | Exclusion criteria | Intervention | |
| 2021 | Taieb, J | CRC | 1 | ctDNA status, clinical risk group, sex, perineural and lymphovascular invasion (VELIPI), and treatment arm | ctDNA, clinical risk group (low versus high), age, sex, ECOG PS (0 versus 1–2), tumor perforation, VELIPI, and treatment arm | A limit of blank (LOB) was determined for each assay used in the study (LOB ¼ 1 for NPY, LOB ¼ 6 for WIF1). When the number of positive droplets was above the LOB, samples were considered for further analysis (see below). All other samples were considered negative. The average fractions of methylated genes was calculated as well as standard deviation of the data and used to determine a “threshold of positivity” equivalent to “averageþ2SD” (0.3% and 0.21% for NPY and WIF1, respectively). For each sample with a number of positive droplets higher than LOB (see above), the percentage of MetctDNA among total detectable ccfDNA was calculated [i.e., %MetctDNA ¼ (MetctDNA/ccfDNA) 100] to overcome sample quality differences possibly due to suboptimal pre-analytical conditions. The total detectable ccfDNA was estimated from the unmethylated Albumin gene sequence included in the triplex assay. The obtained percentage of MetctDNA was compared with the “threshold of positivity” determined as described above. All results above this threshold were considered positive and the remaining ones as negative. | Eligible patients were age ≥18 years, with stage III histologically confirmed colon cancer, curative intent surgery no more than 8 weeks before randomization, an Eastern Cooperative Oncology Group performance status (ECOG PS) of 0 or 1, and postoperative carcinoembryonic antigen (CEA) level <10 ng/mL | no IC for TR sample received a er adjuvant  chemotherapy start technical issue (broken samples, samples never received, no DNA extracted undetermined ctDNA | adjuvant therapy: modified (m)FOLFOX6 or CAPOX | |
| 2022 | Tie, J | CRC |  | —— | —— | For plasma DNA samples partitioned into 12 or 24 wells, ctDNA was classified as detectable (ctDNA-positive) or undetectable (ctDNA-negative). This classification was based on exact permutation tests as described previously1-3 that compared the difference between the average mutant allele frequency (MAF) across the wells containing the sample of interest with that of the wells containing the control  sample for each mutation. One-sided p-values were calculated using the permTS function of the R perm package (version 3.5.1). A sample was classified as ctDNA positive if the p-value was <0.1. For plasma samples divided into 95 wells, the mutant allele frequencies of all observed mutations were used to model the amplicon-specific distribution of assay noise. The p-values corresponding to the mutations of interest (i.e., those detected in the primary tumor tissue) were combined using Fisher’s method to calculate a final p-value for the patient. A patient was classified as ctDNA positive using the same p-value threshold (<0.1) described above. Further technical details of these assays  will be reported elsewhere. | patients with resected histologically confirmed stage II (T3 or T4, N0, M0)18 colon or rectal adenocarcinoma with negative resection margins. To be eligible for enrollment, patients needed to have an Eastern Cooperative Oncology Group (ECOG) performance-status score of 0 to 2 (scores range from 0 to 5, with higher numbers reflecting greater disability) and had to be medically able to receive adjuvant oxaliplatinbased or single-agent fluoropyrimidine chemotherapy; Patients were enrolled within 3 weeks after surgery, and an adequate specimen from the resected tumor needed to be provided for mutation analysis by 4 weeks after surgery | Patients with evidence of macroscopic metastatic disease on computed tomography (CT) of the chest, abdomen, and pelvis performed within 8 weeks before enrollment were excluded. Other exclusion criteria were a history of another primary cancer within the previous 3 years, the presence of synchronous primary colorectal cancer, or treatment with neoadjuvant chemoradiotherapy | adjuvant chemotherapy | |
| 2021 | Benhaim, L. | CRC | 1 | age, sex, stage, sidedness, and ACT | —— | When the number of positive droplets was above the LOB the samples were considered positive and the ctDNA concentration was calculated. Samples presenting low positive droplet content (> LOB but < LOD) or ambiguous results were re-tested using increased concentration of extracted DNA when possible. When necessary a new aliquot of plasma sample was re-extracted. In order to ensure ≥2% sensitivity, negative samples with number of albumin genomes < (LOB of the specific assay+1)*50 were considered non-informative (NI).  In addition, DNA extracted from the buffy coat fraction was analyzed for potential background signal (lysis of blood-cells). The presence of methylation in WIF1 and NPY genes by Met-ddPCR was tested in 10 patients’ buffy-coat. The average fraction of methylated genes was used to determine a “threshold of positivity” equivalent to “average+1SD”. The average percentage of NPY and WIF1 were 0.06% (std-dev= 0.11%) and 0.23% (std-dev= 0.2%) respectively. | patients aged Superior to 18 and inferior to 85 Colon or rectal cancer stage II and III should be surgically treated informed consent signed | patients suffered from synchronous metastasis disease in initial cancer diagnosis patients with 2 synchronous colorectal cancers receiving chemotherapy or radiotherapy, before operation |  | |
| 2022 | Li,Y | CRC | 1 | CMSs classification, clinical risk, age, sex, tumor location, histological type, histological grade, lymphovascular invasion, nerve invasion, CMS subgroup, clinical risk, | —— | The AVENIO software employs a hotspot caller to call single nucleotide variants (SNVs) or small insertions/deletions (INDELs) of interest and an adaptive caller that models the error distribution of each of the twelve substitution types in the sample to build sample-specific and substitution-specific depth thresholds. Patients were classified as ctDNA-positive or ctDNA-negative in pre- and post-chemo plasma based on the presence of previously identified SNVs or INDELs in tumor tissue | 1) Aged ≥18 years; 2) Patients should have colon cancer, whose tumor located at least 12cm from anal verge; 3) Patients with colon cancers undergo radical resection at Fudan University Shanghai Cancer Center (FUSCC); 4) Confirmed adenocarcinoma with AJCC stage III colon cancer by postoperative pathological assessment; 5) Patients should be assessed to be able to receive standard adjuvant chemotherapy for at least 3 months; 6) Patient written informed consent; 7) Plasma samples with ≥4ml available. | 1) Patients with rectal cancers, whose tumors locate within 12cm from anal verge; 2) Patients who have received neoadjuvant chemotherapy or radiotherapy; 3) Patients who need adjuvant radiotherapy; 4) Patients who are intolerable to have adjuvant chemotherapy for at least 3 months; 5) Patients without surgical tissue samples. |  | |
|  |  | CRC | 3 | CMSs classification, clinical risk, age, sex, tumor location, histological type, histological grade, lymphovascular invasion, nerve invasion, CMS subgroup, clinical risk, | —— |  |  |  |  | |
| 2021 | Loupakis, F | CRC | 1 | age, sex, site of metastasis, margins, postoperative treatment, and preoperative treatment | —— | Plasma samples with ≥ 2 SNVs detected above a predefined confidence threshold were deemed ctDNA-positive, and ctDNA concentration was reported as mean tumor molecules per mL of plasma. | —— | —— |  | |
| 2019 | Tarazona, N | CRC | 1 | tumor site, T and N stage, vascular and perineural invasion | —— | —— | (i) histologically proven resectable colon adenocarcinoma and (ii) no radiological evidence of metastatic disease. | Patients with a previous malignancy were excluded |  | |
|  |  | CRC | 2 | tumor site, nodal involvement | —— |  |  |  |  | |
| 2022 | Henriksen, T. V | CRC | 1 | age, MMR, resection, pT,tumor differentiation, CEA | —— | A previously validated cutoff of ≥2 variants detected was used as criteria for ctDNA positivity | Patients were eligible if scheduled for curative intent treatment, and no metastatic disease was evident on CT of chest, abdomen, and pelvis before surgery | —— |  | |
|  |  | CRC | 2 | CEA | —— | —— | —— | —— |  | |
|  |  | CRC | 3 | age | —— | —— | —— | —— |  | |
| 2022 | McNamara, S | CRC | 1 | neoadjuvant therapy | —— | If a tissue baseline variant was detected in cfDNA with a significant adjusted p-value, the patient was defined ctDNA+ | | —— |  | |
| 2019 | Reinert, T | CRC | 1 | stage, lymphovascular invasion, gender | —— | As previously, a plasma sample with at least 2 variants with a confidence score above a predefined algorithm threshold was defined as ctDNA positive | patients with stages I to III CRC from May 1, 2014, to January 31, 2017, at the surgical departments of Aarhus University Hospital, Randers Hospital, and Herning Hospital in Denmark. | —— |  | |
|  |  | CRC | 3 | sex |  |  |  |  |  | |
|  |  |  | 2 | lymphovascular invasion, sex |  |  |  |  |  | |
| 2021 | Bryant,C | CRC | 1 | —— | —— | —— | Pts with mCRC intending to undergo a curative intent procedure were prospectively recruited at two US sites | —— |  | |
| 2022 | Han, S. W. | CRC |  | —— | —— | —— | Patients with stage II-III CRC treated with standard care surgery and/or adjuvant therapy with archived surgical tissue and preoperative and postoperative 3- week plasma samples were selected from a multi-center, prospective CRC cohort. For ctDNA MRD assay, AlphaLiquid Detect (IMBdx, Seoul, Korea) was used | —— |  | |
| 2021 | Anandappa, Gayathri | CRC | 1 | age, sex, laterality, stage, number of lymph node resected, MSI |  | —— | The study recruited patients diagnosed with stage II-III CRC (n=122), including a subset of rectal patients who underwent tri-modality treatment (TMT) | —— |  | |
| 2023 | Kotani, D | CRC | 1 | age, sex, Performance status, Pathological T stage, Pathological N stage, MSS, BRAF, RAS | —— | Plasma samples with at least 2 out of 16 tumor-specific variants detected above a predefined threshold were defined as ctDNA positive | (1) Histopathologically diagnosed with adenocarcinoma. (2) The primary location of the tumor is the colon (cecum, colon, and rectosigmoid) or rectum (excluding appendix, and anal canal cancer). (3) The clinical stage is equivalent to stage II, III in the UICC 8th edition, and a radical resection has been planned, or the lesion is a colon and rectal cancer of Stage IV or relapse (M1) for which R0 resection has been scheduled. (4) The age at the time of acquisition of informed consent is 20 years or older. (5) Eastern Cooperative Oncology Group (ECOG) Performance Status (PS) is 0 or 1. (6) The subject has given a written informed consent for participation in this study. | (1) Having active double cancer. (2) Pregnant or breastfeeding women. (3) Having serious complication. (4) Positive for HBs antigen or positive for HCV antibody. (5) HIV antibody positive (a patient may enroll even if HIV antibody has not been tested). (6) The study doctor judged that it is inappropriate for you to be a subject for this study |  | |
| 2023 | Hofste, Lisa S. M. | CRC |  | —— | —— | Based on this evaluation, the variant-specific background was calculated for every variant by multiplying the mean variant-specific background noise by fifteen and only variants with at least four unique mutant reads and a VAF higher than the variant-specific background were designated as true plasma variants. Plasma samples with at least one tumor specific variant detected were called positive for ctDNA. The number of mutant molecules per ml plasma was calculated with the mean mutant VAF, volume of plasma used for isolation and the total number of unique deduplicated cfDNA molecules. | patients with rectal cancer who underwent neoadjuvant therapy in the Radboud university medical center, Institute for Radiation Oncology Arnhem, or Institute Verbeeten between November 2017 and July 2021 were consecutively enrolled. Both patients with early-stage rectal cancer (cT1-3N0) and locally advanced rectal cancer (cT1-3Nþ and cT4Nx) were included | —— |  | |
| 2021 | Chen, G | CRC | 1 | pathological stage, lymphovascular invasion, nerve invasion, preoperative ctDNA status | —— | All qualified variants identified in the primary tumor of each patient were regarded as patient-specific somatic variants for further ctDNA tracking. A plasma sample was declared as ctDNA-positive if the number of true variants detected | patients with stage II/III CRC from  September 2017 to March 2020 at Fudan University  Shanghai Cancer Center, the Second Affiliated Hospital  of Zhejiang University School of Medicine and Sun Yatsen University Cancer Center. | —— |  | |
|  |  | CRC | 3 | pathological stage, lymphovascular invasion, CEA status, preoperative ctDNA status | |  |  |  |  | |
|  |  | CRC | 2 | pathological stage, lymphovascular invasion, CEA status, preoperative ctDNA status | |  |  |  |  | |
| 2019 | Tie, J | CRC | 1 | Sex, ypT, ypN, pCR, Adjuvant chemotherapy, Postoperative CEA | —— | ctDNA was classified as detectable (ctDNA positive) or undetectable (ctDNA negative) based on a permutation test that compared the mutation frequency in the sample of interest with the mutation frequencies in controls. First, the MAF, defined as the ratio between the number of supermutants and the number of UIDs for the mutation of interest, was calculated for each well with >200UIDs. The difference in the distributions of the MAFs between the sample of interest and the controls was then statistically evaluated via an exact permutation test, using the permTS function of the R perm package (R software V.3.3.1). The one-sided test was used to avoid attributing significance to a ctDNA-negative sample that had fewer supermutants than the associated control. A P value of 0.1 was then chosen as the significance threshold to classify a sample of interest as ctDNA positive (P<0.1) or ctDNA negative | 1. Patients with histologically confirmed adenocarcinoma of the rectum 2. Patients with radiologically defined locally advanced disease on pelvic MRI (mT3, mT4 or mN+) or endorectal ultrasound (uT3, uT4 or uN1) where MRI is contraindicated) 3. No evidence of metastatic disease on CT chest/abdomen/pelvis 4. Patients planned to receive long-course chemoradiation followed by curative surgery and adjuvant 5-fluorouracil or capecitabine chemotherapy 5. Patients willing to provide written informed consent 6. Minimum age 18 Years | 1. History of another primary cancer within the last 5 years, with the exception of nonmelanomatous skin cancer and carcinoma in situ of the cervix. 2. Medical or psychiatric condition or occupational responsibilities that may preclude compliance with the protocol 3. Patients with major organ impairment 4. Patients that are not accessible for follow-up |  | |
| 2016 | Tie, J | CRC | 1 | T stage | —— | ctDNA was classified as detectable (ctDNA-positive) or undetectable (ctDNA-negative) on the basis of a permutation test that compared the mutation frequency in the sample of interest with the mutation frequencies in controls. First, the MAF, defined as the ratio between the number of supermutants and the number of UIDs for the mutation of interest, was calculated for each well with >200 UIDs. The difference in the distributions of the MAFs between the sample of interest and the controls was then statistically evaluated with the permutation test, using the permTS function of the R package perm (R software version 3.2.3). The one-sided test was used to avoid attributing significance to a ctDNA-negative sample that has fewer supermutants than the associated control. A 0.1 P value was then chosen as the threshold to classify a sample of interest as ctDNA-positive (P < 0.1) or ctDNA-negative. Given the lack of a gold standard, a specificity of at least 0.90 was  considered desirable, and a P value equal to 0.1 yielded 0.90 specificity when performing leave-one-out cross-validation on the controls. | Patients with histologically confirmed primary colorectal cancer For the stage II colon cancer (cohort A) -Patients with curatively resected stage II primary colon cancer -Patients must be registered for study within 10 weeks of surgery For the metastatic (stage IV) colorectal cancer (cohort B) -Patients with measurable disease by RECIST criteria version 1.1 -Patients fit to receive standard first line combination chemotherapy, with cytotoxic agents and any biological therapy given at standard doses. ECOG performance status 0 - 2 Patients willing to provide written informed consent Minimum age 18 Years | 1. History of another primary cancer within the last 5 years, with the exception of non-melanomatous skin cancer and carcinoma in situ of the cervix. 2. Medical or psychiatric condition or occupational responsibilities that may preclude compliance with the protocol. 3. Patients with major organ impairment. 4. Patients that are not accessible for follow-up. |  | |
|  |  | CRC | 1 | T stage | —— | —— | —— | —— |  | |
| 2023 | Watanabe, Jun | CRC | 1 | NA | —— | —— | (1) Histopathologically diagnosed with adenocarcinoma. (2) The primary location of the tumor is the colon (cecum, colon, and rectosigmoid) or rectum (excluding appendix, and anal canal cancer). (3) The clinical stage is equivalent to stage II, III in the UICC 8th edition, and a radical resection has been planned, or the lesion is a colon and rectal cancer of Stage IV or relapse (M1) for which R0 resection has been scheduled. (4) The age at the time of acquisition of informed consent is 20 years or older. (5) Eastern Cooperative Oncology Group (ECOG) Performance Status (PS) is 0 or 1. (6) The subject has given a written informed consent for participation in this study. | (1) Having active double cancer. (2) Pregnant or breastfeeding women. (3) Having serious complication. (4) Positive for HBs antigen or positive for HCV antibody. (5) HIV antibody positive (a patient may enroll even if HIV antibody has not been tested). (6) The study doctor judged that it is inappropriate for you to be a subject for this study. |  | |
| 2022 | Zhou, Jian | CRC |  | —— | —— | —— | patients diagnosed with stage I-III CRC from May 2019 to Jun 2020 at the First Affiliated Hospital of Soochow University | |  | |
| 2023 | Mo, S. | CRC | 1 | Histologic type, Tumor differentiation, T stage, Nodal involvement, Invasion, CEA | —— | The ctDNA positive sample was defined as having at least 1 positive marker; otherwise, it was defined as ctDNA negative. Patients were defined as positive if the ColonAiQ tests remained unchanged and positive for all time points or as negative if the ColonAiQ tests were negative for all time points. A marker was considered positive if its Ct in the test was lower than its cut-off Ct (Cttest < Ctcutoff). A sample was considered positive if any of the six markers were positive. | Male or female ≥ 18 years of age on the day of signing informed consent. Patients must have histologically confirmed colorectal cancer. Patients need to receive surgical resection. Patients must have a performance status of ≤1 on the ECOG Performance Scale. Life expectancy of more than 5 years. Written informed consent must be obtained from patient or patient's legal representative and ability for patient to comply with the requirements of the study. | Patients received adjuvant treatment prior to the surgical resection. Patients received blood transfusion two weeks before or during the surgical resection. Patients with unresected advanced colorectal adenoma. Patients who are positive for Human Immunodeficiency Virus (HIV), Hepatitis B or Hepatitis C. Patients who are pregnant. Patients who are alcoholic or drug abusers. Patients with a history or current evidence of any condition or abnormality that might confound the results of the study, interfere with the patient's participation for the full duration of the study, or is not in the best interest of the patient to participate, in the opinion of the Investigator. |  | |
| 2022 | Li, N | NSCLC |  | —— | —— | At least 1 true shared mutation identified in both the tumor sample and a plasma sample from the same patient was defined as positive for ctDNA. | Postoperative histopathological diagnosis of TNM stage IIA to IIIA NSCLC with R0 resection; No previous chemotherapy, radiotherapy, surgery or biological therapy for lung cancer； Eastern Cooperative Oncology Group (ECOG) behavior status score 0 to 1 | Patients with other cancers other than NSCLC within five years prior to this study; Who cannot get enough tumor histological specimens (non-cytological) for analysis; Human immunodeficiency virus (HIV) infection; NSCLC mixed with patients with small cell lung cancer; Pregnant or lactating women; There is a clear history of neurological or mental disorders, including epilepsy or dementia; Conditions that investigators think is not suitable for inclusion |  | |
| 2020 | Peng, M | NSCLC |  | —— | —— | The ctDNA status was classified as detectable (ctDNA-positive) or undetectable (ctDNA-negative) according to mutation ratio. A mutation ratio equal to 0 is defined as ctDNA-negative and greater than 0 is defined as ctDNA-positive. | Eligible patients were age >18 years old with solitary lung nodules who agreed to the curative-intent treatment in this study. | —— |  | |
| 2020 | Kuang, P. P. | NSCLC |  | —— | —— | For an individual patient, we define a ctDNA positive event as at least one shared mutation identified simultaneously in the plasma and tumor specimens. Otherwise, it will be defined as ctDNA negative. | Postoperative histopathological diagnosis of TNM stage IIA to IIIA NSCLC with R0 resection; No previous chemotherapy, radiotherapy, surgery or biological therapy for lung cancer； Eastern Cooperative Oncology Group (ECOG) behavior status score 0 to 1. | Patients with other cancers other than NSCLC within five years prior to this study; Who cannot get enough tumor histological specimens (non-cytological) for analysis; Human immunodeficiency virus (HIV) infection; NSCLC mixed with patients with small cell lung cancer; Pregnant or lactating women; There is a clear history of neurological or mental disorders, including epilepsy or dementia; Conditions that investigators think is not suitable for inclusion. |  | |
| 2022 | Wang, S | NSCLC | 1 | age, sex, smoking, stage, LNM, focality, Histology | —— | To maximize the prognostic value of ctDNA detection, all plasma samples with clonal mutations or subclonal mutations of VAF greater than 0.5% detected were defined as ctDNA-positive, otherwise ctDNA-negative. | 1. Aged 18-80 years; 2. Pathologically confirmed patients with resectable stage I-IIIA non-small cell lung cancer; 3. Patient follow-up information is complete, and plasma and peripheral blood samples, as well as paraffin and/or fresh tissue samples of surgical tissue can be obtained; 4. Patients with multiple primary or metastatic lesions (lymph nodes or solitary metastases, etc.) were preferentially enrolled in the group; 5. Subjects read and fully understand the patient's instructions and sign informed consent. | 1.For patients who are unable to obtain tissue and blood samples, blood samples should include at least two time points: pre-operative (d0) and post-operative (d7); 2.Current alcoholics or drug abusers; 3.pregnant women, women planning to become pregnant, or women who are breast feeding should not participate in the study; 4.Active infections of clinical significance or any form of acute disease; 5.The patient had a history of other tumors or had received antineoplastic treatment before admission. 6.It is estimated that patients' compliance to participate in this clinical study is insufficient. 7.Received other drug clinical trials in the past 4 weeks. |  | |
|  |  | NSCLC | 2 | age, sex, smoking, stage, LNM, focality, Histology | —— |  |  |  |  | |
|  |  |  | 1 | age, sex, smoking, stage, LNM, focality, Histology | —— |  |  |  |  | |
|  |  |  | 1 | age, sex, smoking, stage, LNM, focality, Histology | —— |  |  |  |  | |
| 2021 | Qiu, B | NSCLC | 1 | Histology, T staging, TP53 | —— | ctDNA positivity was defined by accessing the presence of one or more mutations identified in the matched tumor sample in ctDNA. | 1. Aged >=18 years old, male or female; 2. Patients with stage II/III lung cancer confirmed by histology and/or cytology; 3. acceptable for radical operation; 4. ECOG<=1; 5. Expected lifetime over 12 months; 6. Get informed consent from the patient. | 1. Patients who had neoadjuvant therapy before surgery; 2. Patients with other malignant tumors; 3. Patients who had Blood transfusion during or 2 weeks before surgery; 4. Patients with Any severe or uncontrolled systemic disease that significantly affects the patient's risk/benefit balance, including uncontrolled hypertension, hepatitis B, hepatitis C, AIDS, rheumatic immune diseases, etc.; 5. Baseline samples (preoperative and postoperative plasma samples, tumor tissue samples) are incomplete for a variety of reasons; 6. patients who missed 3 consecutive plasmas during the whole process of detection, and the ctDNA detected at next monitoring point was positive or confirmed recurrence by imaging; 7. Have a history of alcohol abuse or drug abuse; 8. Pregnant or lactating female patient; 9. Researchers believe that it is not suitable for the group. |  | |
|  |  | NSCLC | 3 | Histology, pTMN stage, T staging, N staging | —— |  |  |  |  | |
|  |  | NSCLC | 2 | Histology, pTMN stage, T staging, TP53 | —— |  |  |  |  | |
| 2023 | Chen, K | NSCLC |  | —— | —— | ctDNA-positive (maxAF>0). The abundance of ctDNA mutations was reflected as the maximum allele frequency (maxAF) and defined as the highest fraction of the mutant allele detected in each sample. | Aged 18 to 80 years Lung cancer was suspected preoperatively. Received curative surgical therapy No malignant tumor history within the past 5 years No being received any treatment prior to resection Patients must have given written informed consent | (1) pure ground-glass opacity (pGGO), (2) history of neoadjuvant therapy, including chemotherapy, radiotherapy, targeted therapy, or immunotherapy prior to surgery, (3) history of a malignant tumor within 5 years, (4) multiple primary lung cancer, which cannot be completely resected, (5) other types of lung cancer rather than NSCLC confirmed by pathological diagnosis, (6) pathological stage IIIB-N3 or IV, (7) unqualified or failure to obtain blood or tissue samples, (8) refused or withdrawn consent, and (9) circumstances inappropriate for this study, such as blood transfusion, gestation, lactation, etc. |  | |
| 2023 | Fu, R | NSCLC |  | —— | —— | Positive ctDNA was defined as at least one variant detected | Eligible patients were aged > 18 years, and with pathological stage I–III lung cancer, and no malignant tumor history within the past 5 years | (a) multiple primary lung cancer, which is characterized by more than one cancerous lesion independent of each other; (b) R1, R2 resection |  | |
| 2020 | Yang, W. | NSCLC |  | —— | —— | ctDNA at each time point was defined as positive if any of the mutation with an abundance of > 0.1 % was detected. | Postoperative histopathological diagnosis of TNM stage IA / IB NSCLC with R0 resection; No adjuvant chemotherapy, radiotherapy, targeted drug therapy or biotherapy after surgery; Men or women of age ≥18 years and <75 years old; ECOG behavior status score 0 to 1; | Patients with other cancers other than NSCLC within five years prior to this study; who cannot get enough tumor histological specimens (non-cytological) for analysis; human immunodeficiency virus (HIV) infection; NSCLC mixed with patients with small cell lung cancer; pregnant or lactating women; There is a clear history of neurological or mental disorders, including epilepsy or dementia; Conditions that investigators think is not suitable for inclusion |  | |
| 2021 | Tan, A. | NSCLC |  | —— | —— | —— | patients diagnosed with stage I-III NSCLC at the National Cancer Centre Singapore between May 2013 and June 2019 | —— |  | |
| 2019 | Chen, K | NSCLC |  | —— | —— | When multiple mutations were assessed, ctDNA was defined as positive if any of the mutations was positive. | patients with suspected lung cancers and intent to undergo curative surgery. All patients had chest CT scans, abdominal and adrenal gland ultrasonograms or CT scans, brain MR images, and bone scans before surgery. PET/CT was not mandatory in every patient. Eligible patients were aged >18 years with no distant metastasis identified by preoperative examination (cStage I–IIIA) and no malignant tumor history within the past 5 years, whose lesions located in lung were not pure ground glass opacity, and neoadjuvant therapy was not performed. | (i) multiple primary lung cancer; (ii) R1, R2 resection during operation; (iii) histology is not non–small cell lung cancer (NSCLC); (iv) negative driver mutation detected in plasma before surgery (time A); (v) germline mutation detected in plasma before surgery (time A); and (vi) unqualified blood samples |  | |
| *2023 | Chen, K | NSCLC | 1 | sex, age, TMN stage, smoking, subtype, tumor site, ADT | —— | For each iteration, the significance site was defined as a cutoff of p value < 0.05, and the sample was deemed to be positive if two or more sites were significant and the sample level p value < 0.005 | Aged 18 to 80 years Lung cancer was suspected preoperatively. Received curative surgical therapy No malignant tumor history within the past 5 years No being received any treatment prior to resection Patients must have given written informed consent | (1) pure ground-glass opacity (pGGO), (2) history of neoadjuvant therapy, including chemotherapy, radiotherapy, targeted therapy, or immunotherapy prior to surgery, (3) history of a malignant tumor within 5 years, (4) multiple primary lung cancer, which cannot be completely resected, (5) other types of lung cancer rather than NSCLC confirmed by pathological diagnosis, (6) pathological stage IIIB-N3 or IV, (7) unqualified or failure to obtain blood or tissue samples, (8) refused or withdrawn consent, and (9) circumstances inappropriate for this study, such as blood transfusion, gestation, lactation, etc. |  | |
|  |  |  | 2 | sex, age, TMN stage, smoking, subtype, tumor site, ADT | —— |  |  |  |  | |
| 2022 | Waldeck, S | NSCLC |  | —— | —— | plasmas samples were considered ctDNA-positive if the following rules applied: (a) detection of at least one predefined SNV from tumor genotyping; (b) a mean AF of at least 0.001% for the plasma sample (see ‘Statistics’ for definition of ‘mean AF’). The AF of total ctDNA was calculated as the mean AFs of all tumor-derived mutations detected by monitoring in blood plasma | Patients with resectable early-stage or locally advanced NSCLC (stage I–III) qualified for inclusion into this trial (DRKS00009521). From October 2014 to June 2018, a total of 33 patients aged ≥ 18 years were enrolled at the University Medical Center Freiburg, Germany. | —— |  | |
| 2022 | Xia, L | NSCLC | 1 | Tumor size, Histology subtype, Pathologic TNM stage, Adjuvant therapies | —— | A mutation in the plasma sample was defined as positive if it had a P value < 0.01. A sample-level combined P value was further calculated, and a threshold of P < 0.01 on the combined P value was applied to call a plasma sample positive | Patients between 18 and 80 years of age and with pathologic stage I–III NSCLC (AJCC 8th) were eligible for inclusion | Patients with multiple primary lung cancers, or pathologic stage IV disease, or non-NSCLC histology, or with a history of malignancy in the past 5 years were excluded from the study. |  | |
| 2022 | Zhang, J. T. | NSCLC |  | —— | —— | A plasma sample with at least one variant detected was  defined as ctDNA-positive: Driver variants ≥4 high-quality reads Passenger variants ≥8 high-quality reads | stage I to III NSCLC (tumor diameter ≥2 cm) treated with definitive  surgery from March 2019 to January 2021 at the Guangdong Provincial People’s Hospital and Guangdong Lung Cancer Institute, with or  without neoadjuvant or adjuvant therapy | —— |  | |
|  |  | NSCLC |  | —— | —— |  |  |  |  | |
| 2023 | Yuan, Shu-Qiang | GC | 1 | Pathological stage, Lymphovascular invasion, Nerve invasion | Pathological stage, Preoperative cancer antigena | a plasma sample was prospectively defined as ctDNA-positive only if one or more variants in the plasma were detected in at least 2% of the primary tumors. The ctDNA levels of each ctDNA-positive plasma was calculated by the number of tumor-specific mutations per ng of cfDNA and per mL of plasma. | Male or female ≥ 18 years of age at first visit. Patients must have histologically confirmed early or intermediate-stage gastric cancer. Patients need to have surgical treatment. Patients must be able to provide sufficient fresh tissue/biopsies or minimum 5-10 FFPE sections for NGS analysis. Patients must be able to follow the study visit schedule and willing to provide peripheral blood samples at the indicated time point. Written informed consent must be obtained from patient or patient's legal representative and ability for patient to comply with the requirements of the study. | Patients who cannot provide peripheral blood samples prior to the surgical treatment will be excluded. Patients with severe infection will be excluded. Patients with other serious disease besides early or intermediate-stage gastric cancer will be excluded. Pregnant women will be excluded. Patients who are alcoholic or drug abusers will be excluded. Patients with a condition or abnormality that in the opinion of the Investigator would compromise the safety of the patient or the quality of the data will be excluded. |  | |
|  |  | GC | 3 | Smoking history | Smoking history |  |  |  |  | |
| 2020 | Leal, A. | GC |  | —— | —— | —— | Ib-IVa (no distant metastases) gastric cancer (histologically proven); tumor bulk in the stomach WHO < 2 Age ≥18 yrs Operable gastric cancer No prior abdominal radiotherapy or chemotherapy Tumor negative laparoscopy when CT suggests peritoneal carcinomatosis Start treatment within 10 working days after registration Written informed consent | T1N0 disease (endoscopic ultrasound) Distant metastases Inoperable patients; due to technical surgery-related factors or general condition Previous malignancy, except adequately treated non-melanoma skin cancer or in-situ cancer of the cervix uteri. Solitary functioning kidney that will be within the radiation field Major surgery within 4 weeks prior to study treatment start, or lack of complete recovery from the effects of major surgery Uncontrolled (bacterial) infections Significant cardiac disorders Continuous use of immunosuppressive agents Concurrent use of the antiviral agent sorivudine or chemically related analogues Hearing loss > CTC grade 1 Neurotoxicity > CTC grade 1 | perioperative chemotherapy (chemotherapy group) vs. preoperative chemotherapy with postoperative chemoradiotherapy (chemoradiotherapy group) | |
| 2020 | Fedyanin, M. | GC | 1 | T, N, and adjuvant chemotherapy | —— | The plasma sample was considered "positive” if the content of ctDNA was more than 0.5 copies of mutant DNA in ml plasma. | patients with diagnosis of GC who received treatment from 2017 to 2019 |  |  | |
| 2020 | Yang, J. | GC | 1 | T staging, Site | T staging, Site | We defined ctDNA detection as the detection of one or more mutations. | Eligible patients underwent gastrectomy with curative intent, followed by adjuvant chemotherapy (SOX) when indicated by standard of care clinical guidelines | |  | |
|  |  | GC | 2 | T staging, Site | T staging, Site |  |  |  |  | |
|  |  | GC | 3 | T staging, Site | T staging, Site |  |  |  |  | |
| 2023 | Xue, Pei | GC |  | —— | —— | —— | patients with stage I-III GC who underwent R0 resection at Ruijin Hospital | —— |  | |
| 2022 | Hata, Tatsuo | PAAD | 1 | Blood loss, Histological PV/SMV invasion, Histological lymph node metastasis | Histological lymph node metastasis, Histological residual tumor, Postoperative CA19-9 | detected KRAS as positive | cytohistologically proven PDAC | unresectable PDAC and those with unavailable samples |  | |
| 2020 | Popova, A. | PAAD |  | —— | —— | Absence or decrease of the mutant allele fractions by 2% were considered a negative result. | patients with diagnosis of PC who received treatment at the Russian cancer research center n.a. N.N. Blokhin from 2017 to 2019 | —— |  | |
| 2020 | Jiang, J. | PAAD | 1 | Differentiation, Lymph node metastasis, Nerve invasion, Vascular invasion | —— | —— | Between July 2016 and September 2018, a total of 27 patients diagnosed with PDAC were enrolled at Zhejiang Provincial People’s Hospital | —— |  | |
| 2019 | Lee, B | PAAD | 1 | Tumor stage, Lymph node status, Resection margin status, Lymphvascular invasion status, Perineural invasion status, ctDNA status | Tumor stage, Lymph node status, Resection margin status, Lymphvascular invasion, Perineural invasion status, ctDNA status | A P-value of 0.1 was chosen as the threshold to classify a sample of interest as ctDNA-positive (P < 0.1) or ctDNA-negative (P > 0.1). | 1. Patients with resectable pancreatic adenocarcinoma who are to have a resection of their tumour. 2. ECOG performance status 0 - 2. 3. Patients willing to provide written informed consent. Minimum age 18 Years | 1. History of another primary cancer within the last 5 years, with the exception of non-melanomatous skin cancer and carcinoma in situ of the cervix. 2. Medical or psychiatric condition or occupational responsibilities that may preclude compliance with the protocol. 3. Patients that are not accessible for follow-up. 4. Preoperative chemotherapy or radiotherapy. |  | |
| 2023 | Wang, Xiuchao | PAAD |  | —— | —— | —— | patients (n = 20) diagnosed with resectable stage I-III PC | —— |  | |
| 2021 | Wang, D. S | CRLM | 1 | The following factors had no significant influence on the results: age, sex, Primary tumor, Nodal involvement of primary tumor, Time between primary tumor and liver metastases, Diameter of the largest LM, Preoperative CEA level, Number of LM, CRS, Preoperative chemotherapy, Postoperative chemotherapy, Concomitant ablation, KRAS, BRAF | | A sample was defined as positive when variant allele frequency (VAF) ≥ 2% for WES Plus and ≥ 0.5% for the ctDNA 451-gene panel | 1) pathologically and radiologically diagnosed with CRLM; 2) underwent liver metastasectomy with curative intent; 3) presence of tissue samples and, or blood samples for analysis. | 1) unresectable hepatic or extrahepatic metastasis; 2) at least one blood sample not available  during and after treatment; 3) incomplete clinical information; 4) history of other cancers |  | |
|  |  | CRLM | 3 | The following factors had no significant influence on the results: age, sex, Primary tumor, Nodal involvement of primary tumor, Time between primary tumor and liver metastases, Diameter of the largest LM, Preoperative CEA level, Number of LM, CRS, Preoperative chemotherapy, Postoperative chemotherapy, Concomitant ablation, KRAS, BRAF | | | | |  | |
| 2023 | Liu, W | CRLM | 1 | ctDNA status, postoperative, CA199 value, bilobar or unilobar localization, metastasis, lesion number, preoperative chemotherapy, preoperative CA199 value, and KRAS mutation | | Based on the filtered mutations, the blood sample was classified as ctDNA-positive if it contained mutations. Otherwise, it was classified as ctDNA-negative. | The patients underwent radical resection of the primary tumor and liver metastases. The inclusion criteria specified patients who had resectable CRLM before hepatic resection as determined by a multidisciplinary team (MDT), metastasis identified as liver-limited disease, preserved liver function (indocyanine green [ICG] < 10%), and no other simultaneous malignancies. | Patients who underwent only ablation or palliative hepatic resection (R2) were excluded from the study |  | |
| 2022 | Nishioka, Y. | CRLM | 1 | Tumor number, Pathological tumor viability, Somatic RAS/TP53 status, Somatic SMAD4 status | | —— | patients who underwent curative-intent liver resection for CLM | —— |  | |
| 2017 | Michael J | CRLM |  | —— | —— | —— | CRC patients (pts) undergoing hepatic resections with curative intent | —— |  | |
| 2022 | Newhook, T. E | CRLM |  | —— | —— | plasma samples were classified as positive for ctDNA using a proprietary, predefined variant classifier [variant classifier approach; (Guardant Health, Inc, Redwood, CA)]. Before this study, ctDNA profiles from more than 4000 patients with CRC were used to train the variant classifier to differentiate tumor-derived alterations from non-tumor-derived alterations to increase the specificity of postoperative ctDNA detection using a plasma-only approach (Guardant Health, Inc, Redwood, CA). For all outcome analyses presented in this study, ctDNA status was defined using the variant classifier without dependence on paired tissue. | Patients with CLM who were candidates for curativeintent hepatectomy | Patients were excluded if they had clear extrahepatic disease except lung nodules, underwent R2 resection, or did not complete the second stage of planned 2-stage hepatectomy | | |
| 2021 | Bolhuis, K. | CRLM | 1 | age, sex, Fong CRS, radiological response, sidedness and R-status | —— | —— | Histological proof of colorectal cancer Initially unresectable metastases confined to the liver according to CT scan, obtained ≤3 weeks prior to registration. Unresectability should be confirmed by the liver expert panel. Patients with small (≤ 1 cm) extrahepatic lesions that are not clearly suspicious of metastases are eligible Known mutation status of RAS and BRAF WHO performance status 0-1 (Karnofsky performance status ≥ 70) Age ≥ 18 years No contraindications for liver surgery In case of primary tumor in situ: tumor should be resectable. In case of resected primary tumor: adequate recovery from surgery Adequate organ functions, as determined by normal bone marrow function (Hb ≥ 6.0 mmol/L, absolute neutrophil count ≥ 1.5 x 109/L, platelets ≥ 100 x 109/L), renal function (serum creatinine ≤ 1.5x ULN and creatinine clearance, Cockroft formula, ≥ 30 ml/min), liver function (serum bilirubin ≤ 2 x ULN, serum transaminases ≤ 5x ULN) Life expectancy > 12 weeks Expected adequacy of follow-up Written informed consent | Extrahepatic metastases, with the exception of small (≤ 1 cm) extrahepatic lesions that are not clearly suspicious of metastases Unresectable primary tumor Serious comorbidity or any other condition preventing the safe administration of study treatment (including both systemic treatment and surgery) Major cardiovascular events (myocardial infarction, severe/unstable angina, congestive heart failure, CVA) within 12 months before randomisation Uncontrolled hypertension, or unsatisfactory blood pressure control with ≥3 antihypertensive drugs Previous systemic treatment for metastatic disease; previous adjuvant treatment is allowed if completed ≥ 6 months prior to randomisation Previous surgery for metastatic disease Previous intolerance of study drugs in the adjuvant setting Pregnant or lactating women Second primary malignancy within the past 5 years with the exception of adequately treated in situ carcinoma of any organ or basal cell carcinoma of the skin, or second primary colorectal cancer. Any concomitant experimental treatment | Patients in whom local treatment of CRLM is achieved continue postoperatively with the preoperative systemic regimen but without the targeted agent for a total duration of pre- and postoperative treatment of six months. RAS mutated tumours treated with bevacizumab plus either doublet or triplet chemotherapy, | |
| 2023 | Jiang, H | CRLM |  | —— | —— | Plasma samples with at least one mutation detected above a predefined confidence threshold were deemed ctDNA positive. | pathologically confirmed colorectal cancer, primary lesions resected, and underwent liver metastasectomy with curative intent. | extrahepatic metastasis and a history of other cancers |  | |
| 2017 | Schøler, L. V | CRLM |  | —— | —— | —— | colorectal cancer patients undergoing surgical treatment | —— |  | |
| 2021 | Tie, J | CRLM | 1 | baseline CEA, number of liver metastases, diameter of largest liver metastasis, time interval from diagnosis of primary tumor to liver metastases, and primary tumour N stage | —— | ctDNA is quantified as mutant allele fraction (MAF), defined as the ratio between the number of “supermutants” (a mutation present in >90% of reads in a unique identifier family with the same molecular barcode) and the number of unique identifier sequences that contain the normal (wild-type) form at the nucleotide of interest | 1. Patients with histologically confirmed primary colorectal cancer 2. Patients with resected primary tumours or planned for curative primary tumour resection. 3. Patients with resectable liver metastases following workup including CT scans of chest, abdomen and pelvis (or MRI if unable to undertake CT scan) and whole body PET scan. 4. Patients fit for surgery 5. Patients fit for combination chemotherapy (5-FU and oxaliplatin) 6. Patients willing to provide written informed consent 7. Minimum age 18 Years | 1. History of another primary cancer within the last 5 years, with the exception of non-melanomatous skin cancer and carcinoma in situ of the cervix. 2. Medical or psychiatric condition or occupational responsibilities that may preclude compliance with the protocol 3. Patients that are not accessible for follow-up |  | |
|  |  | CRLM | 3 | —— | —— |  |  |  |  | |
| 2022 | Reinert, T | CRLM | 1 | pN | —— | Samples with a ctDNA VAF above the LOD and with at least three positive droplets were called positive. | (a) No extrahepatic disease, (b) Eligibility for a R0 resection leaving at least 25% of functioning liver parenchyma and (c) A performance status allowing liver surgery | No post-OP blood (n = 29)  Primary tumor not resected (n = 7)  Synchronous other cancer；due to no assay for analysis |  | |
|  |  | CRLM | 2 | pN |  |  |  |  |  | |
| 2022 | Schneider, B. P. | BC | 1 | Age, race, body mass index, clinical tumor size, pathologic, tumor size, pathologic nodal status, histologic grade, residual cancer burden classification, and prior therapy | Age, race, body mass index, clinical tumor size, pathologic, tumor size, pathologic nodal status, histologic grade, residual cancer burden classification, and prior therapy | —— | 1. Written informed consent and HIPAA authorization for release of personal health information. 2. Age ≥ 18 years at the time of consent. 3. ECOG Performance Status 0 or 1 within 14 days prior to study registration. 4. Women and men of childbearing potential must be willing to use an effective method of contraception (e.g. hormonal or barrier method of birth control; abstinence) from the time consent is signed until 4 weeks after protocol therapy discontinuation. 5. Women of childbearing potential must have a negative pregnancy test within 30 days prior to study registration. Women should be counseled regarding acceptable birth control methods to utilize from the time of screening to start of treatment. If prior to treatment after discussion with the subject it is felt by the treating physician there is a possibility the subject is pregnant a pregnancy test should be repeated. 6. Women must not be breastfeeding. 7. Must have histologically or cytologically confirmed triple negative (ER-/PR-/HER2-) invasive breast cancer, clinical stage I-III at diagnosis (AJCC 6th edition) based on initial evaluation by physical examination and/or breast imaging prior to study registration. 8. Must have completed preoperative (neoadjuvant) chemotherapy. 9. Must have completed definitive resection of primary tumor. For those that do not require radiotherapy, the most recent surgery for breast cancer must have been completed at least 14 days prior, but no more than 84 days prior, to study registration. 10. Must have significant residual invasive disease at the time of definitive surgery following preoperative chemotherapy. Significant residual disease is defined as at least one of the following: 11. Must have an FFPE tumor block with tumor cellularity of 20% or greater. 12. BREAST RADIOTHERAPY 13. No stage IV (metastatic) disease, however no specific staging studies are required in the absence of symptoms or physical exam findings that would suggest distant disease. 14. No treatment with any investigational agent within 30 days prior to study registration. 15. No history of chronic hepatitis B or untreated hepatitis C. 16. Adequate laboratory values must be obtained within 14 days prior to study registration. 17. Left ventricular ejection fraction within normal limits obtained within 30 days prior to study registration. 18. No clinically significant infections as judged by the treating physician. 19. Must consent to allow submission of adequate archived tumor tissue sample from definitive surgery for genomic assessment of tumor. 20. Must consent to collection of whole blood samples for genomic analysis. 21. No clinically significant arrhythmia or baseline ECG abnormalities in the opinion of the treating physician. 22. No active second malignancy (except non-melanomatous skin cancer or incidental prostate cancer found on cystectomy): Active second malignancy is defined as a current need for cancer therapy or a high possibility (> 30%) of recurrence during the study. Previous contralateral breast cancer is allowable unless it meets “active” criteria as stated above | —— | drug selections were decided by the MTB. Drug selection was based on patient’s NGS findings with attention to prior therapies, comorbidities, and prior toxicities; drug selection, dose, and modifications were made by the patient’s treating oncologist and could have included no therapy. | |
| 2022 | Sharma, P | BC |  | —— | —— | Samples demonstrating pathogenic/likely pathogenic variant(s) with 3-40% allelic frequencies were considered ctDNA positive | patients with RD post-NACT and available EOT plasma samples who were enrolled in an IRB-approved multisite prospective registry between 2011 and 2018 | |  | |
| 2019 | Coombes, R. C. | BC |  | —— | —— | As previously, a plasma sample with at least 2 variants with a confidence score above a predefined algorithm threshold (0.97) was defined as ctDNA positive. | Eligible patients were 18 years or older, displayed no clinical evidence of metastatic disease, and were therefore considered free of disease after surgery and adjuvant chemotherapy | patients who did not fulfill the trial entry criteria |  | |
| 2017 | Chen, Y. H. | BC | 1 | RCB and number of positive lymph nodes | —— | —— | TNBC patients who have residual disease after neoadjuvant chemotherapy. Eligibility criteria required residual disease defined as either: (i) residual tumor >2 cm in the breast; (ii) lymph node involvement; or (iii) RCB classification of II or III | —— |  | |
| 2021 | Zhou, Y | BC |  | —— | —— | —— | (1) tumors larger than 5 cm with regional lymphadenopathy (N1–N3); (2) cancers that involve the skin of the breast or the underlying muscles of the chest; (3) presence of regional lymphadenopathy (clinically fixed or matted axillary lymph nodes, or any infraclavicular, supraclavicular or internal mammary lymphadenopathy) regardless of tumor stage; or (4) inflammatory breast cancer. | —— |  | |
| 2015 | Garcia-Murillas, Isaac | BC | 1 | clinicopathological factors including molecular subtypes, clinical tumor size and pathological lymph nodal status | —— | —— | Patients (table S5) were recruited from the Royal Marsden Hospital and were treated with standard therapy | —— |  | |
|  |  | BC | 2 | clinicopathological factors including molecular subtypes, clinical tumor size, and lymph nodal status | |  |  |  |  | |
| 2020 | Openshaw, M. R. | ESCA |  | —— | —— | —— | patients were recruited including palliative patients and undergoing curative treatment. | —— |  | |
| 2023 | Takei, Shogo | ESCA |  | —— | —— | —— | 1) Esophageal cancer, esophago-gastric junction cancer, gastric cancer by clinical or histological diagnosis 2) Patients who plan to curative treatment 3) Age >= 20 years old. 4) Written informed consent obtained. | 1) Patients with double cancer (excluding early or superficial cancers which can be curatively resected by endoscopic treatment) 2) Any other patients who are regarded as inadequate for study enrollment by principal investigators. |  | |
| 2023 | Morimoto, Y. | ESCA |  | —— | —— | —— | (1) pathologically diagnosed as ESCC; (2) underwent transthoracic esophagectomy at Keio University Hospital between January 2020 and May 2022; (3) pStage 0, I, II, III, or IV ESCC due to supraclavicular LN metastasis; (4) received NAC | (1) salvage esophagectomy after definitive chemoradiotherapy; (2) R2 resection. Clinical staging was determined using EGD and CT in all patients before treatment. |  | |
| 2023 | Gerlinger, Marco | ESCA |  | —— | —— | —— | Male/female patients aged ≥18 years Histologically confirmed gastric, gastro-oesophageal junction or oesophageal adenocarcinoma (referred to as gastro-oesophageal adenocarcinoma (GOA) in this protocol). Oesophageal and gastric tumours should be TNM7 stage T1-4 and N0-N2, with no evidence of distant metastases (M0) where the MDT believes that an R0 resection can be achieved after pre-operative chemotherapy. Absence of distant metastases on CT scan and PET scan and staging laparoscopy (where indicated) prior to study entry No prior therapy for GOA Considered fit for surgery by surgical/anaesthetic team Adequate bone marrow function: Absolute neutrophil count (ANC) >1.5x10-9/L White blood count >3x10-9/L Platelets ≥100x10-9/L Haemoglobin (Hb) >9g/dL (can be post-transfusion) Adequate renal function: Creatinine Clearance of >50ml/min or measured EDTA Clearance of ≥50ml/min. If the calculated Creatinine Clearance is <60ml/min then a measured EDTA Clearance is required. If available, the EDTA Clearance should always take precedence over the Creatinine Clearance. Adequate liver function Serum bilirubin <22 umol/L ALT/AST ≤2.5x ULN Adequate coagulation profile International Normalised Ratio (INR) < 1.5 Activated Prothrombin Time (APTT) < 1.5xULN Patients on oral anticoagulation are advised to change to low molecular weight heparin prior to study entry, to be eligible ECOG performance status 0 or 1 Body Mass Index (BMI) ≤30 Patient is fit to undergo all protocol investigations and receive all protocol treatment based on the assessment in the surgical and oncology clinics. Signed and dated informed consent document indicating that the patient (or legally acceptable representative) has been informed of all the pertinent aspects of the trial prior to enrolment. Willingness and ability to comply with the protocol for the duration of the study including scheduled visits, examinations, investigations and treatment plans | Any contraindication or known hypersensitivity reaction to any of the study drugs, or components of Folinic acid, Oxaliplatin, 5FU or Docetaxel Known severe hypersensitivity reactions to monoclonal antibodies (Grade ≥ 3 NCI CTCAE v 4.0), any history of anaphylaxis, or uncontrolled asthma (i.e., 3 or more features of partially controlled asthma) If known dihydropyrimidine dehydrogenase (DPD) deficiency, patients must be deemed safe to receive appropriate dose-adjusted 5-FU according to the identified mutation. Patients who have received anti-PD-1, anti-PD-L1, anti-PD-L2, anti-CD137, or anti-CTLA-4 antibody, or any other antibody or drug specifically targeting T-cell co-stimulation or checkpoint pathways Prior malignancy active within the previous 3 years except for locally curable cancers that have been apparently cured, such as basal or squamous cell skin cancer, superficial bladder cancer, or carcinoma in situ of the prostate, cervix, or breast. Patients recommended to have radiotherapy as part of routine management for their GOA are ineligible Any immunodeficiency disorder Any active autoimmune disease that has required systemic treatment in the past 2 years (i.e, with use of disease modifying agents, corticosteroids or immunosuppressive drugs) or is expected to deteriorate when receiving avelumab, with the following exceptions:  Patients only receiving hormone replacement therapy (eg, thyroxine, insulin, or physiologic corticosteroid replacement therapy (doses ≤10mg - or equivalent - of prednisolone per day) for adrenal or pituitary insufficiency) are eligible. Patients with vitiligo or psoriasis not requiring immunosuppressive treatment are eligible Patients requiring hormone replacement with corticosteroids are eligible if the steroids are administered only for the purpose of hormonal replacement Administration of steroids through a route known to result in minimal systemic exposure (topical, intranasal intra-ocular, or inhalation) are acceptable. Steroids as pre-medication for hypersensitivity reactions e.g. CT contrast are also acceptable Prior organ transplantation, including allogeneic stem-cell transplantation History of inflammatory bowel disease with the following exception: Patients with a history of ulcerative colitis who have had a colectomy are eligible Patients with a history of interstitial lung disease or radiological evidence of pulmonary fibrosis Cerebrovascular disease (including transient ischaemic attacks (TIA) and strokes) within the previous year Cardiovascular diseases as follows: Myocardial infarction within the previous year Serious cardiac arrhythmia requiring medication (for example, ventricular tachycardia, supraventricular tachycardia or atrial fibrillation with a resting heart rate > 110bpm) Unstable angina Congestive cardiac arrhythmia (New York Heart Association Classification Class II or above) Other severe acute or chronic medical conditions or psychiatric conditions including recent (within the past year) active suicidal ideation or behaviour Current signs or symptoms of any other severe progressive or uncontrolled hepatic, haematologic, gastrointestinal, endocrine, respiratory or cardiac disease other than directly related to gastrooesophageal adenocarcinoma, which in the opinion of the investigator, might impair the subject's tolerance of trial treatment or procedures. Major surgery, major trauma or open biopsy within 28 days prior to registration (not including staging laparoscopy) Evidence of bleeding diathesis or coagulopathy Active infection requiring systemic therapy, non-healing wound, ulcer or bone fracture requiring therapy Known positive tests for human immunodeficiency virus (HIV) infection Active Hepatitis A, B or C infection. If there is a previous history of Hepatitis infection which has been treated and cleared, there must be evidence that disease is not currently active. Known peripheral neuropathy > grade 1 (absence of deep tendon reflexes as the sole neurological abnormality does not render the patient ineligible) Use of live attenuated vaccine within 28 days of initiation of study therapy, or anticipation that a live attenuated vaccine will be required during the study Pregnancy/of child bearing potential. Pregnancy must be excluded with a negative serum pregnancy test, within 7 days before initiation of therapy, if the risk of conception exists. Sexually active female patients must be surgically sterile or be postmenopausal or must agree to use highly effective contraception. Sexually active male patients must be surgically sterile or must agree to use highly effective contraception, i.e. methods with a failure rate of <1% per year (see section 5.4 for full definition and examples of highly effective contraception). Lactation- breast-feeding is contraindicated and must be discontinued for the duration of the trial and for at least 1 month afterward the last dose of Avelumab. Any patient specific factors which are likely to interfere with compliance of trial specific procedures or treatment. | |  |
| 2021 | Liu, T. | ESCA | 1 | sex, Age, Smoking, Alcohol, Tumor location, Maximum tumor diameter, Tumor stage, Histological grade, Depth of submucosal invasion, Angiolymphatic invasion | sex, Age, Smoking, Alcohol, Tumor location, Maximum tumor diameter, Tumor stage, Histological grade, Depth of submucosal invasion, Angiolymphatic invasion | For candidate cfDNA variant detection, the following criteria were applied: 1) No germline or clonal hematopoietic mutations; 2) not present in the 1000G data; 3) MAF > 0.02%; 4) variant-supporting consensus reads of ≥3. | 1) underwent surgery; 2) did not undergo any anti-tumor treatment before the first blood collection; 3) had no history of other malignancies | three patients whose FFPE samples were not available, and two patients with insufficient tissue samples were excluded from the study | | |
| 2021 | Ococks, E. | ESCA | 2 | chemotherapy response, lyphm node status | —— | Patients were classified as ctDNA positive following surgery if one or more plasma samples taken at any timepoint after surgery had at least two variants detected | patients treated with neoadjuvant chemotherapy and surgery were identified from the prospective | ？ |  | |
| 2023 | Ananda, Sumitra | OV |  | —— | —— | —— | 1. Patients that have had primary debulking surgery for curatively resected stage I-IV high grade serous, endometrioid or clear cell carcinoma, or carcinosarcoma of the ovary, fallopian tube or primary peritoneum. Stage IV patients can only be included in the study if they have had a complete resection of all macroscopic disease with no residual disease. OR Patients commencing neoadjuvant chemotherapy for stage I-III high grade serous, endometrioid or clear cell carcinoma, or carcinosarcoma of the ovary, fallopian tube or primary peritoneum. Women must be planned to undergo interim debulking surgery. 2. A representative tumour sample can be made available for molecular testing after surgery or a core biopsy pre neoadjuvant chemotherapy if available. 3. Fit and planned for adjuvant chemotherapy. Minimum age 18 Years Sex Females | 1. History of another primary cancer within the last 3 years, with the exception of non-melanomatous skin cancer, carcinoma in situ of the cervix and fully resected stage1a endometrial cancer 2. Patients with EOC of mucinous subtype and sarcoma 3. Patients with Stage IV disease who have residual disease 4. Patients <18 years |  | |
| 2022 | Chao, A. | OV | 1 | ctDNA_C1, Residual disease | ctDNA_C1, Residual disease | Among tumor variants, single-nucleotide variants (SNV) with at least 20 reads and an allele frequency greater than 0.5% were considered as true variants. Indels present in at least 20 reads and an allele frequency greater than 0.1% were considered true variants. | (1) patients with epithelial ovarian cancer, tubal or peritoneal primary cancer; (2) age 18e85 years old; (3) patients underwent primary or interval debulking surgery after neoadjuvant therapy; (4) International Federation of Gynecology and Obstetrics (FIGO) stage I-IV, and (5) signed informed consent. For advanced ovarian cancer (stage III or IV), treatment option was based on physician's discretion to remove all macroscopic disease at primary or interval surgery | (1)non-epithelial ovarian cancer (n ¼ 4); (2) borderline tumors of ovary (n ¼ 12); (3) tumor percentage <15% (n ¼ 3); (4) plasma not available before treatment and after 7e10 days after surgery (n ¼ 4), and (5) patients with pregnancy |  | |
| 2021 | Chapman, Jocelyn S. | OV |  | —— | —— | —— | I-IV EOC | —— |  | |
| 2022 | Carrasco, R | BLCA | 1 | cfDNA level and fragmentation pattern at each follow-up time point and clinicopathological variables | —— | —— | who underwent RC and extended lymphadenectomy at our center between 2018 and 2019 were prospectively included | The exclusion criterion was the presence of another active neoplasm. Follow-up data were available for all patients |  | |
| 2021 | Powles, T | BLCA | 1 | nodal status, PD-L1 status, tumour stage, prior neoadjuvant chemotherapy and number of lymph nodes | —— | The presence of two or more of the patient-specific tumour mutations in the plasma defined ctDNA positivity, maintaining high specificity (>99.8%) that has been validated across numerous studies2 | Histologically confirmed muscle-invasive UC (also termed transitional cell carcinoma) of the bladder or upper urinary tract (i.e., renal pelvis or ureters) For participants treated with prior neoadjuvant chemotherapy: tumor stage of ypT2-4a or ypN+ (ypT2-4 or ypN+ for participants with upper urinary tract UC) and M0 For participants who have not received prior neoadjuvant chemotherapy: tumor stage of pT3-4a or pN+ (pT3-4 or pN+ for participants with upper urinary tract UC) and M0 Representative formalin-fixed paraffin-embedded tumor specimens from surgical resection (i.e., radical cystectomy, nephroureterectomy, or lymph node dissection) in paraffin blocks (blocks preferred) or at least 15 unstained slides, with an associated pathology report, for central testing and determined to be evaluable for tumor programmed death-ligand 1 (PD-L1) expression prior to study enrollment Absence of residual disease and absence of metastasis, as confirmed by a negative baseline computed tomography (CT) or magnetic resonance imaging scan of the pelvis, abdomen, and chest no more than 4 weeks prior to randomization Full recovery from cystectomy or nephroureterectomy within 14 weeks following surgery Eastern Cooperative Oncology Group performance status of less than or equal to (</=) 2 Life expectancy greater than or equal to (>/=) 12 weeks Adequate hematologic and end-organ function For women who are not postmenopausal or surgically sterile: agreement to remain abstinent or use contraceptive methods that result in a failure rate of less than (<) 1 percent (%) per year during the treatment period and for at least 5 months after the last dose of atezolizumab | Any approved anti-cancer therapy within 3 weeks prior to initiation of study treatment Adjuvant chemotherapy or radiation therapy for UC following surgical resection Treatment with any other investigational agent or participation in another clinical trial with therapeutic intent within 28 days or five half-lives of the drug prior to enrollment Malignancies other than UC within 5 years prior to Cycle 1, Day 1 Pregnancy or breastfeeding Significant cardiovascular disease Severe infections within 4 weeks prior to Cycle 1, Day 1 Major surgical procedure other than for diagnosis within 28 days prior to Cycle 1, Day 1 History of severe allergic, anaphylactic, or other hypersensitivity reactions to chimeric or humanized antibodies or fusion proteins Known hypersensitivity to biopharmaceuticals produced in Chinese hamster ovary cells or any component of the atezolizumab formulation History of autoimmune disease Prior allogeneic stem cell or solid organ transplant History of idiopathic pulmonary fibrosis, organizing pneumonia, drug-induced pneumonitis, idiopathic pneumonitis, or evidence of active pneumonitis on screening chest CT scan Positive test for human immunodeficiency virus and/or active hepatitis B or hepatitis C or tuberculosis Administration of a live, attenuated vaccine within 4 weeks before Cycle 1 Day 1 Prior treatment with cluster of differentiation 137 (CD137) agonists or immune checkpoint blockade therapies, including anti-CD40, anti-cytotoxic T-lymphocyte-associated protein 4 (anti-CTLA-4), anti-programmed death-1 (anti-PD-1), and anti-PD-L1 therapeutic antibodies | —— | |
| 2023 | Powles, T | BLCA |  | —— | —— | same as Powles, T-2021 | same as Powles, T-2021 | same as Powles, T-2021 | —— | |
| 2022 | Szabados, B. | BLCA |  | —— | —— | Whole exome sequencing of tumor tissue from baseline and matched normal specimen from whole blood were performed. This allowed identification of clonal somatic single nucleotide variants (SNVs), from which 16 SNVs were selected for inclusion in a multiplex PCR-NGS ctDNA assay. The designed assays were then used to assess ctDNA levels in plasma. This method has defined and validated ctDNA positivity based on the presence of two or more variants | Willing and able to provide written informed consent Ability to comply with the protocol Age ≥ 18 years Histopathologically confirmed transitional cell carcinoma (T2-T4a) of the bladder. Patients with mixed histologies are required to have a dominant transitional cell pattern. Residual disease after TURBT (surgical opinion, cystoscopy or radiological presence). Fit and planned for cystectomy (according to local guidelines). N0 or M0 disease CT or MRI (within 4 weeks of registration) Representative formalin-fixed paraffin embedded (FFPE) bladder tumour samples with an associated pathology report that are determined to be available and sufficient for central testing. Patients who refuse neoadjuvant cisplatin based chemotherapy or in whom neoadjuvant cisplatin based therapy is not appropriate. Eastern Cooperative Oncology Group (ECOG) Performance Status of 0 or 1 Negative pregnancy test within 2 weeks of Day 1 Cycle 1 for female patients of childbearing potential. For female patients of childbearing potential to use a highly effecting form(s) of contraception (i.e. one that results in a low failure rate [<1% per year] when used consistently and correctly) and to continue its use for 90 days after the last dose of MPDL3280A. Adequate hematologic and end-organ function within 4 weeks prior to the first study treatment | Pregnant and lactating female patients. Major surgical procedure within 4 weeks prior to enrolment or anticipation of need for a major surgical procedure during the course of the study other than for diagnosis. Previously intravenous chemotherapy for bladder cancer. Patients with prior allogeneic stem cell or solid organ transplantation. Prior treatment with CD137 agonists, anti-CTLA-4, anti-programmed death-1 (PD-1), or anti-PD-L1 therapeutic antibody or pathway-targeting agents. Patients must not have had oral or IV steroids for 14 days prior to study entry. The use of inhaled corticosteroids, physiologic replacement doses of glucocorticoids (i.e., for adrenal insufficiency), and mineralocorticoids (e.g., fludrocortisone) is allowed. Received therapeutic oral or intravenous (IV) antibiotics within 14 days prior to enrolment (Patients receiving prophylactic antibiotics (e.g., for prevention of a urinary tract infection or chronic obstructive pulmonary disease) are eligible). Administration of a live, attenuated vaccine within 4 weeks prior to enrolment or anticipation that such a live, attenuated vaccine will be required during the study. Treatment with systemic immunostimulatory agents (including but not limited to interferons or interleukin [IL]-2) within 4 weeks or five half-lives of the drug, whichever is shorter, prior to enrolment. Treatment with any other investigational agent or participation in another clinical trial with therapeutic intent within 4 weeks prior to enrolment. Evidence of significant uncontrolled concomitant disease that could affect compliance with the protocol or interpretation of results, including significant liver disease (such as cirrhosis, uncontrolled major seizure disorder, or superior vena cava syndrome). Malignancies other than UBC within 5 years prior to Cycle 1, Day 1, with the exception of those with a negligible risk of metastasis or death and treated with expected curative outcome (such as adequately treated carcinoma in situ of the cervix, basal or squamous cell skin cancer, or ductal carcinoma in situ treated surgically with curative intent) or localized prostate cancer treated with curative intent and absence of prostate-specific antigen (PSA) relapse or incidental prostate cancer (Gleason score ≤ 3 + 4 and PSA < 10 ng/mL undergoing active surveillance and treatment naive). Severe infections within 4 weeks prior to enrolment in the study including but not limited to hospitalization for complications of infection, bacteraemia, or severe pneumonia. Significant cardiovascular disease, such as New York Heart Association cardiac disease (Class II or greater), myocardial infarction within 3 months prior to enrolment, unstable arrhythmias, or unstable angina. History of idiopathic pulmonary fibrosis (including pneumonitis), drug-induced pneumonitis, organizing pneumonia (i.e., bronchiolitis obliterans, cryptogenic organizing pneumonia), or evidence of active pneumonitis on screening chest CT scan (History of radiation pneumonitis in the radiation field (fibrosis) is permitted). Patients with uncontrolled Type 1 diabetes mellitus. Patients with Type 1 diabetes controlled on a stable insulin regimen are eligible. Patients with active hepatitis infection (defined as having a positive hepatitis B surface antigen [HBsAg] test at screening) or hepatitis C. Patients with past hepatitis B virus (HBV) infection or resolved HBV infection (defined as having a negative HBsAg test and a positive antibody to hepatitis B core antigen [anti-HBc] antibody test) are eligible. Patients positive for hepatitis C virus (HCV) antibody are eligible only if polymerase chain reaction (PCR) is negative for HCV RNA. Positive test for HIV Patients with active tuberculosis History of gastrointestinal disorders (medical disorders or extensive surgery) which may interfere with the absorption of the study drug. Uncontrolled hypercalcemia (> 1.5 mmol/L ionized calcium or Ca > 12 mg/dL or corrected serum calcium > the institutional ULN) or symptomatic hypercalcemia requiring continued use of bisphosphonate therapy or denosumab. Patients who are receiving bisphosphonate therapy or denosumab specifically to prevent skeletal events and who do not have a history of clinically significant hypercalcemia are eligible. Patients who are receiving denosumab prior to enrollment must be willing and eligible to receive a bisphosphonate instead while on study. History of autoimmune disease including but not limited to myasthenia gravis, myositis, autoimmune hepatitis, systemic lupus erythematosus, rheumatoid arthritis, inflammatory bowel disease, vascular thrombosis associated with antiphospholipid syndrome, Wegener's granulomatosis, Sjögren's syndrome, Guillain-Barré syndrome, multiple sclerosis, vasculitis, or glomerulonephritis. Patients with a history of autoimmune-related hypothyroidism, unless on a stable dose of thyroid-replacement hormone. History of severe allergic, anaphylactic, or other hypersensitivity reactions to chimeric or humanized antibodies or fusion proteins Known hypersensitivity or allergy to biopharmaceuticals produced in Chinese hamster ovary cells or any component of the MPDL3280A formulation |  | |
| 2019 | Christensen, Emil | BLCA | 2 | Pathological downstaging, T stage at diagnosis, N stage before CX, ctDNA after CX. | —— | A plasma sample with at least two variants with a confidence score above a predefined algorithm threshold (0.97) was defined as ctDNA positive. | Ninety-nine patients diagnosed with MIBC and who were receiving neoadjuvant chemotherapy before cystectomy were prospectively enrolled between 2013 and 2017 at Aarhus University Hospital in Denmark | —— |  | |
| 2019 | Tan, L. | melanoma | 1 | BRAF mutation, AJCC substage | —— | ctDNA was defined as detectable if there was 1 copy of mutant DNA detected in both duplicate reactions. Uniform criteria were applied to select mutations for ctDNA monitoring | Between March 2011 and December 2016, 133 patients with newly diagnosed stage III resected cutaneous melanoma were enrolled into the Melanoma Research Victoria (MRV) study | —— |  | |
| 2022 | Genta, Sofia | melanoma | | —— | —— | —— | Patients with either histological confirmation of a solid tumor or hematological malignancy, OR patients identified as high-risk for cancer (based on identified aberration in cancer predisposition gene or on hormonal and/or family history without known aberration). Patient must be ≥ 18 years old. All patients must have signed and dated an informed consent form for this LIBERATE study. If patients are being co-consented for a separate primary research study listed in Appendix I, they must fulfill the eligibility criteria for that separate primary research study. If there is a discrepancy in the eligibility criteria between protocols, the separate primary research study's criteria take precedence. | —— |  | |
| 2023 | Eroglu, Zeynep | melanoma | | —— | —— | Plasma samples with two or greater SNVs detected above a predefined algorithm's confidence threshold were considered ctDNA‐positive. ctDNA concentration (levels) was reported as mean tumor molecules (MTM) per mL of plasma. | stage III–IV melanoma patients treated between April 2020 to March 2022 | insufficient tumor tissue for assasy design |  | |
| 2022 | Zhao, L | HCC | 2 | Tumor diameter, MVI, Satellite nodules, AJCC stage, Post-operative AFP、DCP、CTC、ctDNA PPWES | —— | —— | (a) pathologic or radiographic diagnosis of HCC; (b) surgical treatment by either hepatectomy or liver transplantation | —— |  | |
| 2022 | Kitahata, Y | PAAD |  | —— | —— | ctDNA positivity was defined as a lower limit for mutant allele frequencies (MAF) at 0.02 % according to previous our report | The patients are planned to treat neoadjuvant chemotherapy according our protocol | The study excluded patients with a diagnosis of unresectable pancreatic cancer by preoperative imaging examinations as well as cases with specimen defects. |  | |
| 2022 | Yamaguchi, T | PAAD | 1 | Resectability status, Pathologic differentiation, Lymph node metastasis, Residual tumor, Adjuvant chemotherapy, Preoperative CA19-9 valuem, Postoperative CA19-9 value, Presence of preoperative ctDNA | Resectability status, Pathologic differentiation, Lymph node metastasis, Adjuvant chemotherapy, Preoperative CA19-9 valuem, Postoperative CA19-9 value, Presence of preoperative ctDNA | Accordingly, the cutoff concentrations in this study for G12D, G12V, and G12R were respectively 3 copies per 1 ml, 5 copies per 1 ml, and 3 copies per 1 ml. | patients with a diagnosis of PDAC in the pancreas head who underwent surgical resection in the Department of Surgery at the Hiroshima University Hospital | A diagnosis of pancreatic adenocarcinoma was confirmed histologically in all cases. Other patients with histologic variants such as mucinous carcinoma, adenosquamous carcinoma, and anaplastic carcinoma were excluded from this study, as were patients positive for peritoneal cytology |  | |
