# Supplementary material for: Circulating tumour DNA-Based molecular residual disease detection in resectable cancers: a systematic review and meta-analysis
Source: eBioMedicine. 2024 Apr 13;103:105109. doi: 10.1016/j.ebiom.2024.105109 (PMC11021841; doi:10.1016/j.ebiom.2024.105109)
Supplement: Table S8 [file mmc8.docx]

Table S8 Meta-regression analysis of sensitivity by bivariate model

|  |  | Estimate | Se | Z | P (z-test) | Ci.lb | Ci.ub |  |
| --- | --- | --- | --- | --- | --- | --- | --- | --- |
|  | Ratio |  |  |  |  |  |  |  |
|  |  | -0.602 | 0.139 | -4.335 | <0.001 | -0.874 | -0.330 |  |
|  | Time |  |  |  |  |  |  |  |
|  |  | 1.025 | 0.172 | 5.944 | <0.001 | 0.687 | 1.363 |  |
|  | Tech (A, B, C) |  |  |  |  |  |  |  |
|  |  | -0.189 | 0.082 | -2.303 | 0.021 | -0.350 | -0.028 |  |
|  | Score |  |  |  |  |  |  |  |
|  |  | -0.008 | 0.098 | -0.08 | 0.936 | -0.200 | 0.184 |  |
|  |  |  |  |  |  |  |  |  |

The time hierarchy is landmark (1) and longitudinal (2); The ratio hierarchy is (0~1), [1,5), [5,9), [9,13); Ratio=the number of patients with ctDNA- / the number of patients with ctDNA+；Score: the quality of studies; Tech (A, B, C): detection technology (A=mPCR-NGS, B=ddPCR, C=hybridization capture-based NGS) in landmark.
