# Supplementary material for: Circulating tumour DNA-Based molecular residual disease detection in resectable cancers: a systematic review and meta-analysis
Source: eBioMedicine. 2024 Apr 13;103:105109. doi: 10.1016/j.ebiom.2024.105109 (PMC11021841; doi:10.1016/j.ebiom.2024.105109)
Supplement: Table S9 [file mmc9.docx]

Table S9 Subgroup for the Sens, Fpr, AUSROC (AUC) and DOR of detection technology of pan-cancer by bivariate model

| Tech | Number of data | Sens | LL | UL | Fpr | LL | UL | Iv2 (Z and D) | DOR | LL | UL | AUC |
| --- | --- | --- | --- | --- | --- | --- | --- | --- | --- | --- | --- | --- |
| A | 16 | 0.54 | 0.47 | 0.61 | 0.08 | 0.05 | 0.11 | 4.60% | 14.20 | 9.35 | 20.60 | 0.80 |
| B | 11 | 0.45 | 0.38 | 0.54 | 0.12 | 0.08 | 0.18 | 0% | 6.18 | 3.65 | 9.82 | 0.69 |
| C | 18 | 0.45 | 0.40 | 0.51 | 0.10 | 0.07 | 0.14 | 0% | 7.96 | 5.42 | 11.30 | 0.65 |
| D | - | - | - | - | - | - | - | - | - | - | - | - |
| E | - | - | - | - | - | - | - | - | - | - | - | - |

A=mPCR-NGS, B=ddPCR, C=hybridization capture-based NGS, D=Guardant Reveal, O=circulating tumour DNA methylation, E=cSMART;

The Iv2 estimated by Zhou and Dendukuri approach (Z and D); 1=landmark detection; 2=longitudinal detection; Sens: sensitivity; Fpr: false-positive rate;

DOR: diagnostic odds ratio; AUSROC (AUC): area under the summary receiver operating characteristic curve.

The Iv2 estimated by Zhou and Dendukuri approach; 1=landmark detection; 2=longitudinal detection; sens: sensitivity

fpr: false-positive rate; DOR:diagnostic odds ratio
