# Supplementary material for: Circulating tumour DNA-Based molecular residual disease detection in resectable cancers: a systematic review and meta-analysis
Source: eBioMedicine. 2024 Apr 13;103:105109. doi: 10.1016/j.ebiom.2024.105109 (PMC11021841; doi:10.1016/j.ebiom.2024.105109)
Supplement: Table S10 [file mmc10.docx]

Table S10 Sensitivity and specificity summary of AUSROC values of Technology by univariate model

|  | Tech | AUSROC | Low | High | P (Chi-square) |  |
| --- | --- | --- | --- | --- | --- | --- |
|  | A | 0.82 | 0.80 | 0.85 | 0.12 |  |
|  | B | 0.76 | 0.69 | 0.85 | 0.72 |  |
|  | C | 0.77 | 0.73 | 0.80 | 0.45 |  |
|  | D | - | - | - | - |  |
|  | E | - | - | - | - |  |
|  | O | - | - | - | - |  |

A=mPCR-NGS, B=ddPCR, C=hybridization capture-based NGS; D=Guardant Reveal, O=circulating tumour DNA methylation, E=cSMART; AUSROC (AUC): area under the summary receiver operating characteristic curve.
