# Supplementary material for: Circulating tumour DNA-Based molecular residual disease detection in resectable cancers: a systematic review and meta-analysis
Source: eBioMedicine. 2024 Apr 13;103:105109. doi: 10.1016/j.ebiom.2024.105109 (PMC11021841; doi:10.1016/j.ebiom.2024.105109)
Supplement: Table S11 [file mmc11.docx]

Table S11 Meta-regression analysis of HR of recurrence and OS for the article quality score

|  |  | Estimate | Se | Z | P (z-test) | Ci.lb | Ci.ub |  |
| --- | --- | --- | --- | --- | --- | --- | --- | --- |
|  | U-Recurrence |  |  |  |  |  |  |  |
|  |  | 0.1360 | 0.0731 | 1.8603 | 0.0628 | -0.0073 | 0.2794 |  |
|  | U-OS |  |  |  |  |  |  |  |
|  |  | -0.1088 | 0.1427 | -0.7621 | 0.4460 | -0.3885 | 0.1710 |  |
|  |  |  |  |  |  |  |  |  |

U: Univariate analysis.
