# Supplementary material for: Circulating tumour DNA-Based molecular residual disease detection in resectable cancers: a systematic review and meta-analysis
Source: eBioMedicine. 2024 Apr 13;103:105109. doi: 10.1016/j.ebiom.2024.105109 (PMC11021841; doi:10.1016/j.ebiom.2024.105109)
Supplement: Supplement Search mode [file mmc12.docx]

**Supplement Methods**

We followed the Preferred Reporting Items for Systematic Reviews and Meta-Analyses statement to report this meta-analysis.

Data Sources and Searches

We systematically searched Pubmed, Embase, Cochrane, and Scopus, supplemented by ASCO, ESMO, and Google, for prospective studies or randomized controlled trials that collected blood samples prospectively. The search period was from Jan 1, 2013, to Sept 10, 2023. Keywords used include “Molecular Residual Disease (MRD),” “circulating tumour DNA,” “surgery,” “Drug Therapy, Adjuvant,” “Chemotherapy,” “Adjuvants, Immunologic,” “Radiotherapy, Adjuvant,” and “Targeted therapy” and their MeSH terms.

The inclusion criteria: (1) prospective studies or randomized controlled trials which collected blood samples prospectively; (2) patients with confirmed resectable cancers (I-IV stage); (3) The analysis group was classified as patients with ctDNA+ and ctDNA-; (4) data available on outcome indicators: disease-free survival (DFS), progression-free survival (PFS), relapse-free survival (RFS), event-free survival (EFS), time to recurrence (TTR), distant metastasis‐free survival (DMFS) or OS was reported as HR; (5) ctDNA testing in postoperative or post-adjuvant therapy after surgery; (6) ctDNA-MRD landmark detection or longitudinal detection in postoperative; (7) only papers published in English. The exclusion criteria: (1) unresectable cancer; (2) Individual cases, non-availability of data (HR of DFS, PFS, RFS, EFS, TTR, DMFS and OS or the number of patients with ctDNA+ and ctDNA-); (3) No pre-designed blood samples were collected or it is not clear if there was a pre-design blood collection; (4) ctDNA present in urine or other body fluids; (5) The time of ctDNA blood collection and analysis was non-postoperation; (6) articles with inconsistent titles and abstracts, reviews, animal tests, and systematic reviews.

The search strategies for PubMed, Embase, Cochrane and scopus were listed follow.

Search filters used in pubmed:

(((((((((((((((((minimal residual disease[MeSH Terms]) OR (residual neoplasms[Title/Abstract])) OR (residual neoplasm[Title/Abstract])) OR (minimal residual disease[Title/Abstract])) OR (minimal disease, residual[Title/Abstract])) OR (residual minimal disease[Title/Abstract])) OR (residual minimal diseasest[Title/Abstract])) OR (residual disease, minimal[Title/Abstract])) OR (minimal residual diseases[Title/Abstract])) OR (residual cancer[Title/Abstract])) OR (cancer, residual[Title/Abstract])) OR (residual cancers,[Title/Abstract])) OR (residual tumor[Title/Abstract])) OR (residual tumours[Title/Abstract])) OR (tumour, residual[Title/Abstract]) AND (2013:2023[pdat]) AND (2013:2023[pdat])) AND (("Circulating Tumor DNA"[Mesh]) OR ((((((DNA, Circulating Tumor[Title/Abstract]) OR (Tumor DNA, Circulating[Title/Abstract])) OR (Cell-Free Tumor DNA[Title/Abstract])) OR (Cell Free Tumor DNA[Title/Abstract])) OR (DNA, Cell-Free Tumor[Title/Abstract])) OR (Tumor DNA, Cell-Free[Title/Abstract])))) OR ((((((((((("Circulating Tumor DNA"[Mesh]) OR (DNA, Circulating Tumor[Title/Abstract])) OR (Tumor DNA, Circulating[Title/Abstract])) OR (Cell-Free Tumor DNA[Title/Abstract])) OR (Cell Free Tumor DNA[Title/Abstract])) OR (DNA, Cell-Free Tumor[Title/Abstract])) OR (Tumor DNA, Cell-Free[Title/Abstract])) AND ((((((("Drug Therapy"[Mesh]) OR (Drug Therapies[Title/Abstract])) OR (Therapies, Drug[Title/Abstract])) OR (Chemotherapy[Title/Abstract])) OR (Chemotherapies[Title/Abstract])) OR (Pharmacotherapy[Title/Abstract])) OR (Pharmacotherapies[Title/Abstract]))) OR (((((((("Circulating Tumor DNA"[Mesh]) OR (DNA, Circulating Tumor[Title/Abstract])) OR (Tumor DNA, Circulating[Title/Abstract])) OR (Cell-Free Tumor DNA[Title/Abstract])) OR (Cell Free Tumor DNA[Title/Abstract])) OR (DNA, Cell-Free Tumor[Title/Abstract])) OR (Tumor DNA, Cell-Free[Title/Abstract])) AND (("Immunotherapy"[Mesh]) OR (Immunotherapies[Title/Abstract])))) OR (((((((("Circulating Tumor DNA"[Mesh]) OR (DNA, Circulating Tumor[Title/Abstract])) OR (Tumor DNA, Circulating[Title/Abstract])) OR (Cell-Free Tumor DNA[Title/Abstract])) OR (Cell Free Tumor DNA[Title/Abstract])) OR (DNA, Cell-Free Tumor[Title/Abstract])) OR (Tumor DNA, Cell-Free[Title/Abstract])) AND ((((((((((((((((((("Radiotherapy"[Mesh]) OR (Radiotherapies[Title/Abstract])) OR (Radiation Therapy[Title/Abstract])) OR (Radiation Therapies[Title/Abstract])) OR (Therapies, Radiation[Title/Abstract])) OR (Therapy, Radiation[Title/Abstract])) OR (Radiation Treatment[Title/Abstract])) OR (Radiation Treatments[Title/Abstract])) OR (Treatment, Radiation[Title/Abstract])) OR (Radiotherapy, Targeted[Title/Abstract])) OR (Radiotherapies, Targeted[Title/Abstract])) OR (Targeted Radiotherapies[Title/Abstract])) OR (Targeted Radiotherapy[Title/Abstract])) OR (Targeted Radiation Therapy[Title/Abstract])) OR (Radiation Therapies, Targeted[Title/Abstract])) OR (Targeted Radiation Therapies[Title/Abstract])) OR (Therapies, Targeted Radiation[Title/Abstract])) OR (Therapy, Targeted Radiation[Title/Abstract])) OR (Radiation Therapy, Targeted[Title/Abstract])))) OR (((((((("Circulating Tumor DNA"[Mesh]) OR (DNA, Circulating Tumor[Title/Abstract])) OR (Tumor DNA, Circulating[Title/Abstract])) OR (Cell-Free Tumor DNA[Title/Abstract])) OR (Cell Free Tumor DNA[Title/Abstract])) OR (DNA, Cell-Free Tumor[Title/Abstract])) OR (Tumor DNA, Cell-Free[Title/Abstract])) AND (((((((("Molecular Targeted Therapy"[Mesh]) OR (Molecular Targeted Therapies[Title/Abstract])) OR (Targeted Therapy, Molecular[Title/Abstract])) OR (Therapy, Molecular Targeted[Title/Abstract])) OR (Targeted Molecular Therapy[Title/Abstract])) OR (Molecular Therapy, Targeted[Title/Abstract])) OR (Targeted Molecular Therapies[Title/Abstract])) OR (Therapy, Targeted Molecular[Title/Abstract]))) AND (2013:2023[pdat]))) OR ((("Circulating Tumor DNA"[Mesh]) OR ((((((DNA, Circulating Tumor[Title/Abstract]) OR (Tumor DNA, Circulating[Title/Abstract])) OR (Cell-Free Tumor DNA[Title/Abstract])) OR (Cell Free Tumor DNA[Title/Abstract])) OR (DNA, Cell-Free Tumor[Title/Abstract])) OR (Tumor DNA, Cell-Free[Title/Abstract]))) AND (("Surgical Procedures, Operative"[Mesh]) OR (((((((((((((((Operative Procedures) OR (Operative Procedure)) OR (Procedure, Operative)) OR (Procedures, Operative)) OR (Surgical Procedure, Operative)) OR (Operative Surgical Procedures)) OR (Procedure, Operative Surgical)) OR (Procedures, Operative Surgical)) OR (Surgical Procedures)) OR (Procedure, Surgical)) OR (Procedures, Surgical)) OR (Surgical Procedure)) OR (Operative Surgical Procedure)) OR (Surgery, Ghost)) OR (Ghost Surgery))) AND (2013:2023[pdat]))

Search filters used in Embase:

| #25 | (#20 OR #21 OR #22) AND [2013-2023]/py |
| --- | --- |
| #24 | (#20 OR #21 OR #22) AND [2013-2023]/py |
| #23 | #20 OR #21 OR #22 |
| #22 | #16 AND #19 |
| #21 | #16 AND #18 |
| #20 | #16 AND #17 |
| #19 | #6 OR #7 OR #8 OR #9 OR #10 OR #11 OR #12 OR #13 OR #14 |
| #18 | #3 OR #4 |
| #17 | #1 OR #2 |
| #16 | #5 OR #15 |
| #15 | 'circulating tumor dna'/exp |
| #14 | 'molecular targeted therapies':ab,ti OR 'targeted therapy, molecular':ab,ti OR 'therapy, molecular targeted':ab,ti OR 'targeted molecular therapy':ab,ti OR 'molecular therapy, targeted':ab,ti OR 'targeted molecular therapies':ab,ti OR 'therapy, targeted molecular':ab,ti |
| #13 | 'molecularly targeted therapy'/exp |
| #12 | 'adjuvant radiotherapy':ab,ti OR 'adjuvant radiotherapies':ab,ti OR 'radiotherapies, adjuvant':ab,ti |
| #11 | 'adjuvant radiotherapy'/exp |
| #10 | 'immunologic adjuvant':ab,ti OR 'immunological adjuvant':ab,ti OR 'adjuvant, immunological':ab,ti OR 'adjuvant, immunologic':ab,ti OR 'adjuvants, immunological':ab,ti OR 'immunological adjuvants':ab,ti OR 'immunoadjuvants':ab,ti OR 'immunologic adjuvants':ab,ti OR 'immunoadjuvant':ab,ti |
| #9 | 'immunological adjuvant'/exp |
| #8 | 'drug therapy, adjuvant':ab,ti OR 'adjuvant chemotherapy':ab,ti OR 'adjuvant drug therapy':ab,ti |
| #7 | 'adjuvant chemotherapy'/exp |
| #6 | 'cancer adjuvant therapy'/exp |
| #5 | 'dna, circulating tumor':ab,ti OR 'tumor dna, circulating':ab,ti OR 'cell-free tumor dna':ab,ti OR 'cell free tumor dna':ab,ti OR 'dna, cell-free tumor':ab,ti OR 'tumor dna, cell-free':ab,ti |
| #4 | 'operative procedures':ab,ti OR 'operative procedure':ab,ti OR 'procedure, operative':ab,ti OR 'procedures, operative':ab,ti OR 'surgical procedure, operative':ab,ti OR 'operative surgical procedures':ab,ti OR 'procedure, operative surgical':ab,ti OR 'procedures, operative surgical':ab,ti OR 'surgical procedures':ab,ti OR 'procedure, surgical':ab,ti OR 'procedures, surgical':ab,ti OR 'surgical procedure':ab,ti OR 'operative surgical procedure':ab,ti OR 'surgery, ghost':ab,ti OR 'ghost surgery':ab,ti |
| #3 | 'surgery'/exp |
| #2 | 'residual neoplasms':ab,ti OR 'residual neoplasm':ab,ti OR 'minimal residual disease':ab,ti OR 'minimal disease, residual':ab,ti OR 'residual minimal disease':ab,ti OR 'residual minimal diseases':ab,ti OR 'residual disease, minimal':ab,ti OR 'minimal residual diseases':ab,ti OR 'residual cancer':ab,ti OR 'cancer, residual':ab,ti OR 'residual cancers':ab,ti OR 'residual tumor':ab,ti OR 'residual tumors':ab,ti OR 'residual tumour':ab,ti OR 'residual tumours':ab,ti OR 'tumour, residual':ab,ti |
| #1 | 'minimal residual disease'/exp |

Search filters used in Scopus:

( ( "minimal residual disease" OR "residual neoplasms" OR "residual neoplasm" OR "minimal residual disease" OR "minimal disease, residual" OR "residual minimal disease" OR "residual minimal diseases" OR "residual disease, minimal" OR "minimal residual diseases" OR "residual cancer" OR "cancer, residual" OR "residual cancers" OR "residual tumor" OR "residual tumors" OR "residual tumour" OR "residual tumours" OR "tumour, residual" ) AND ( "dna, circulating tumor" OR "tumor dna, circulating" OR "cell-free tumor dna" OR "cell free tumor dna" OR "dna, cell-free tumor" OR "tumor dna, cell-free" ) ) OR ( ( "surgery" OR "operative procedures" OR &apos; "operative procedure" OR "procedure, operative" OR "procedures, operative" OR "surgical procedure, operative" OR "operative surgical procedures" OR "procedure, operative surgical" OR "procedures, operative surgical" OR "surgical procedures" OR "procedure, surgical" OR "procedures, surgical" OR "surgical procedure" OR "operative surgical procedure" OR "surgery, ghost" OR "ghost surgery" ) AND ( "dna, circulating tumor" OR "tumor dna, circulating" OR "cell-free tumor dna" OR "cell free tumor dna" OR "dna, cell-free tumor" OR "tumor dna, cell-free" ) ) OR ( ( "adjuvant chemotherapy" OR "drug therapy, adjuvant" OR "adjuvant chemotherapy" OR "adjuvant drug therapy" OR "immunological adjuvant" OR "immunologic adjuvant" OR "immunological adjuvant" OR "adjuvant, immunological" OR "adjuvant, immunologic" OR "adjuvants, immunological" OR "immunological adjuvants" OR "immunoadjuvants" OR "immunologic adjuvants" OR "immunoadjuvant" OR "adjuvant radiotherapy" OR "adjuvant radiotherapy" OR "adjuvant radiotherapies" OR "radiotherapies, adjuvant" OR "molecularly targeted therapy" OR "molecular targeted therapies" OR "targeted therapy, molecular" OR "therapy, molecular targeted" OR "targeted molecular therapy" OR "molecular therapy, targeted" OR "targeted molecular therapies" OR "therapy, targeted molecular" ) AND ( "dna, circulating tumor" OR "tumor dna, circulating" OR "cell-free tumor dna" OR "cell free tumor dna" OR "dna, cell-free tumor" OR "tumor dna, cell-free" ) ) AND PUBYEAR > 2012 AND PUBYEAR < 2024

Search filters used in Cochrane:

#1 MeSH descriptor: [Circulating Tumor DNA] explode all trees

#2 (DNA, Circulating Tumor):ti,ab,kw OR (Tumor DNA, Circulating):ti,ab,kw OR(Cell-Free Tumor DNA):ti,ab,kw OR(Cell Free Tumor DNA):ti,ab,kw OR(DNA, Cell-Free Tumor):ti,ab,kw OR(Tumor DNA, Cell-Free):ti,ab,kw

#3 #1 OR #2

#4 MeSH descriptor: [Surgical Procedures, Operative] explode all trees

#5 (Operative Procedures):ti,ab,kw OR (Operative Procedure):ti,ab,kw OR(Procedure, Operative):ti,ab,kw OR(Procedures, Operative):ti,ab,kw OR(Surgical Procedure, Operative):ti,ab,kw OR(Operative Surgical Procedures):ti,ab,kw OR(Procedure, Operative Surgical):ti,ab,kw OR(Procedures, Operative Surgical):ti,ab,kw OR(Surgical Procedures):ti,ab,kw OR(Procedure, Surgical):ti,ab,kw OR(Procedures, Surgical):ti,ab,kw OR(Surgical Procedure):ti,ab,kw OR(Operative Surgical Procedure):ti,ab,kw OR(Surgery, Ghost):ti,ab,kw OR(Ghost Surgery):ti,ab,kw

#6 #4 OR #5

#7 MeSH descriptor: [Chemotherapy, Adjuvant] explode all trees

#8 (Drug Therapy, Adjuvant):ti,ab,kw OR (Adjuvant Chemotherapy):ti,ab,kw OR(Adjuvant Drug Therapy):ti,ab,kw

#9 MeSH descriptor: [Adjuvants, Immunologic] explode all trees

#10 (Immunologic Adjuvant):ti,ab,kw OR (Immunological Adjuvant):ti,ab,kw OR(Adjuvant, Immunological):ti,ab,kw OR(Adjuvant, Immunologic):ti,ab,kw OR(Adjuvants, Immunological):ti,ab,kw OR(Immunological Adjuvants):ti,ab,kw OR(Immunoadjuvants):ti,ab,kw OR(Immunologic Adjuvants):ti,ab,kw OR(Immunoadjuvant):ti,ab,kw

#11 MeSH descriptor: [Radiotherapy, Adjuvant] explode all trees

#12 (Adjuvant Radiotherapy):ti,ab,kw OR (Adjuvant Radiotherapies):ti,ab,kw OR(Radiotherapies, Adjuvant):ti,ab,kw

#13 MeSH descriptor: [Molecular Targeted Therapy] explode all trees

#14 (Molecular Targeted Therapies):ti,ab,kw OR (Targeted Therapy, Molecular):ti,ab,kw OR(Therapy, Molecular Targeted):ti,ab,kw OR(Targeted Molecular Therapy):ti,ab,kw OR(Molecular Therapy, Targeted):ti,ab,kw OR(Targeted Molecular Therapies):ti,ab,kw OR(Therapy, Targeted Molecular):ti,ab,kw

#15 #7 OR #8 OR #9 OR #10 OR #11 OR #12 OR #13 OR #14

#16 #3 AND #6 with Cochrane Library publication date Between Jan 2013 and Dec 2023

#17 #3 AND #15 with Cochrane Library publication date Between Jan 2013 and Dec 2023

#18 (Residual Neoplasms):ti,ab,kw OR (Residual Neoplasm):ti,ab,kw OR(Minimal Residual Disease):ti,ab,kw OR(Minimal Disease, Residual):ti,ab,kw OR(Residual Minimal Disease):ti,ab,kw OR(Residual Minimal Diseases):ti,ab,kw OR(Residual Disease, Minimal):ti,ab,kw OR(Minimal Residual Diseases):ti,ab,kw OR(Residual Cancer):ti,ab,kw OR(Cancer, Residual):ti,ab,kw OR(Residual Cancers):ti,ab,kw OR(Residual Tumor):ti,ab,kw OR(Residual Tumors):ti,ab,kw OR(Residual Tumour):ti,ab,kw OR(Residual Tumours):ti,ab,kw OR(Tumour, Residual):ti,ab,kw

#19 MeSH descriptor: [Neoplasm, Residual] explode all trees

#20 #18 OR #19

#21 #3 AND #20 with Cochrane Library publication date Between Jan 2013 and Dec 2023

#22 #16 OR #17 OR #21
