# Supplementary figures and images for: Circulating tumour DNA-Based molecular residual disease detection in resectable cancers: a systematic review and meta-analysis
Source: eBioMedicine. 2024 Apr 13;103:105109. doi: 10.1016/j.ebiom.2024.105109 (PMC11021841; doi:10.1016/j.ebiom.2024.105109)

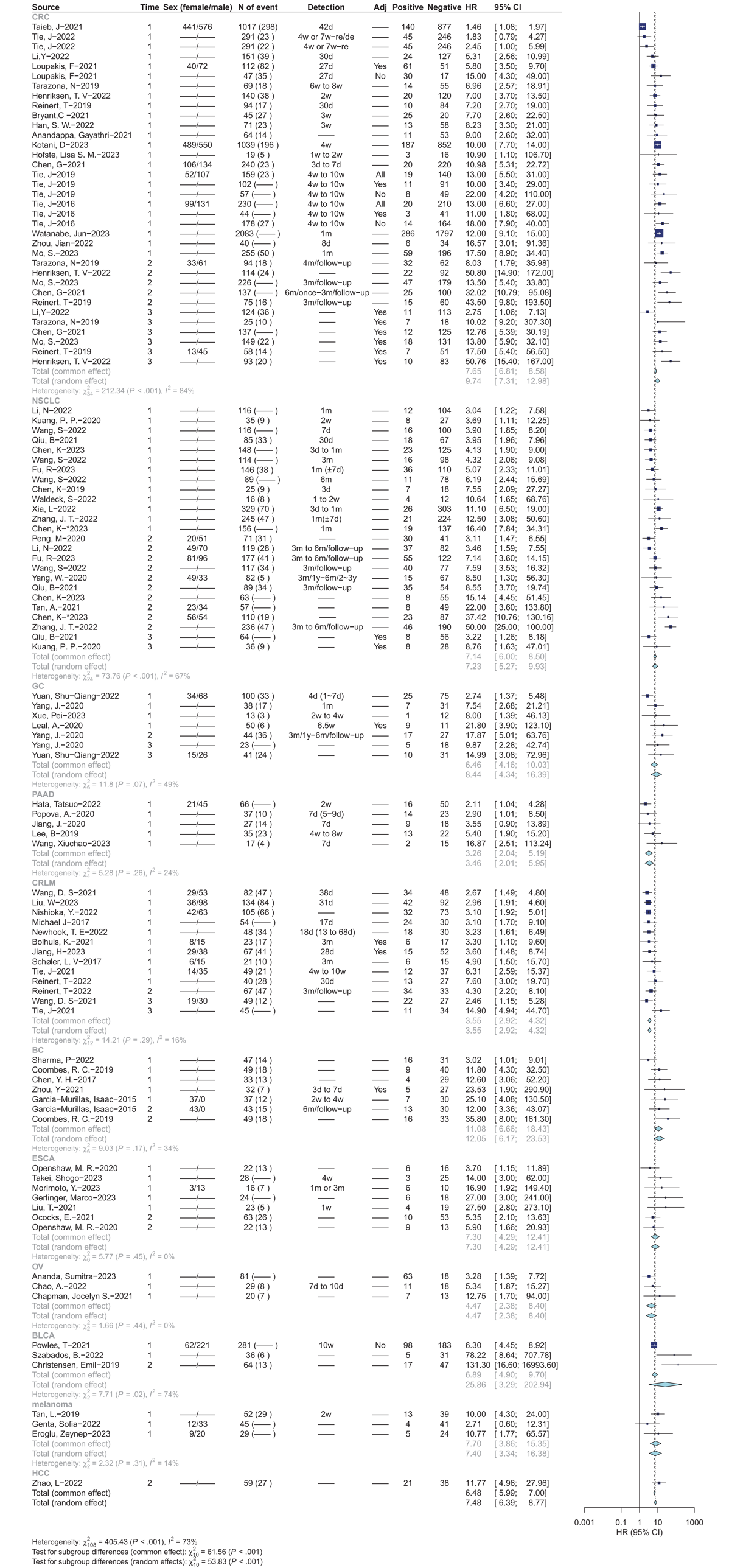

Supplement: Figure S1 [file mmc13.pdf]

a

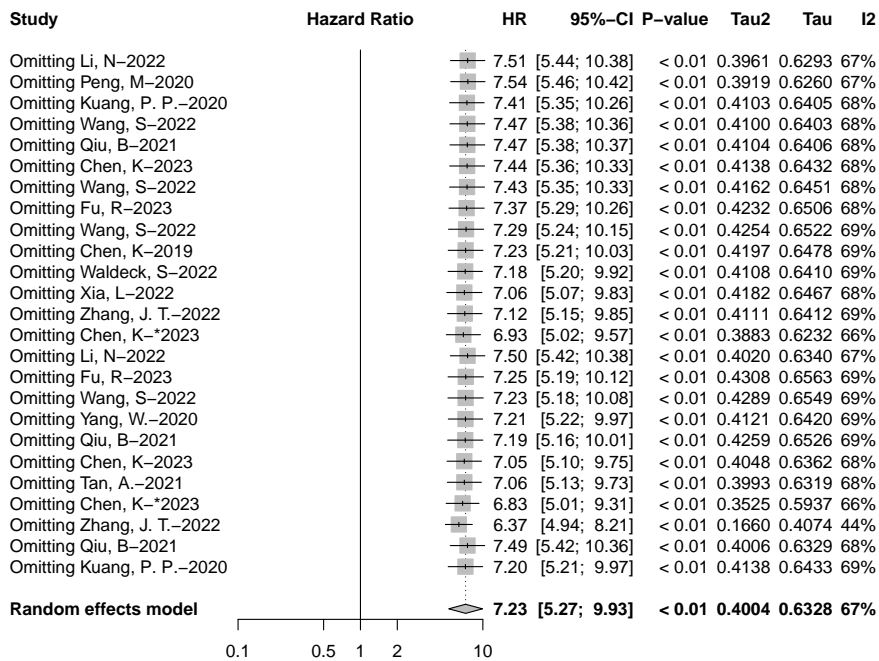

b

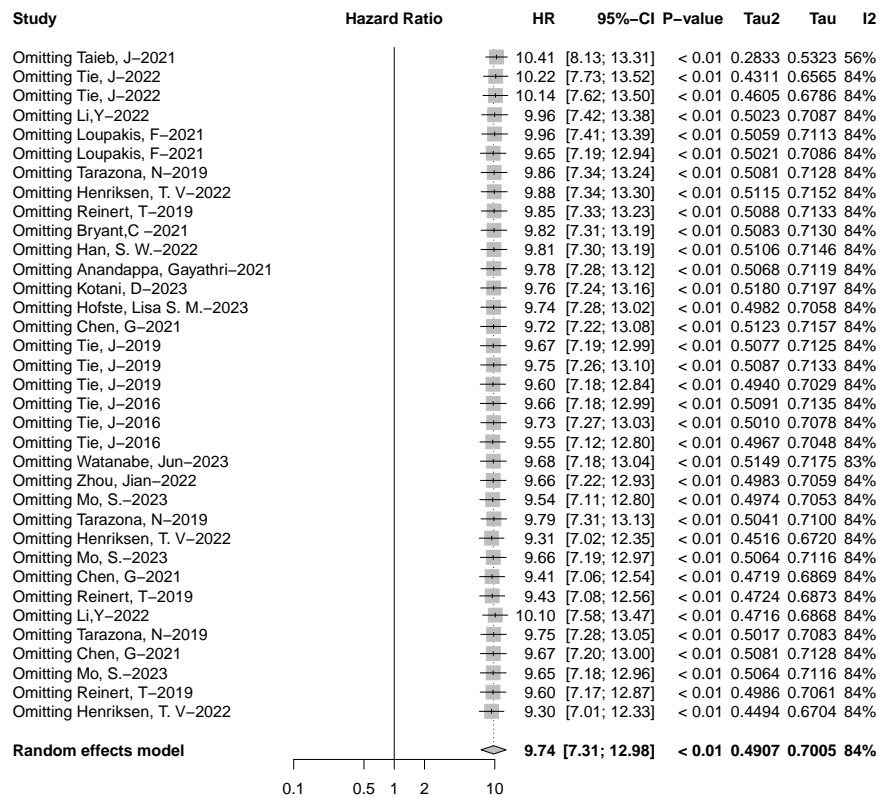

c

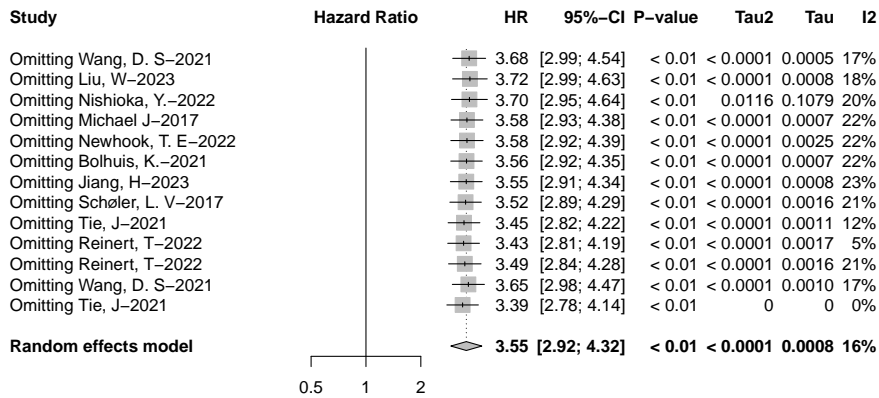

d

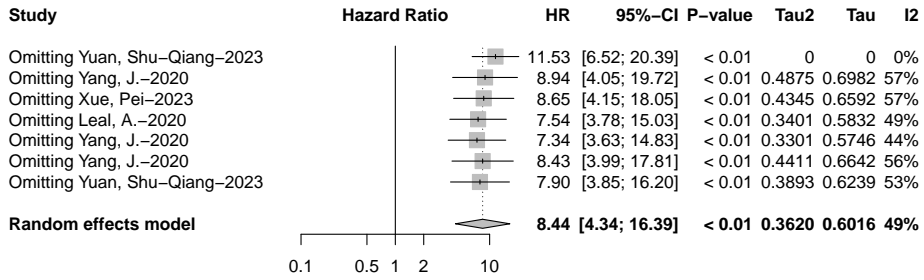

e

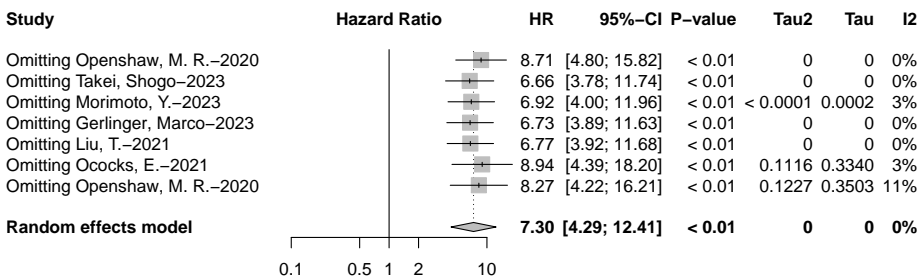

f

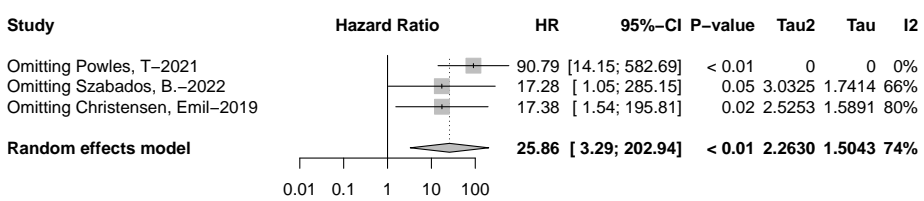

g

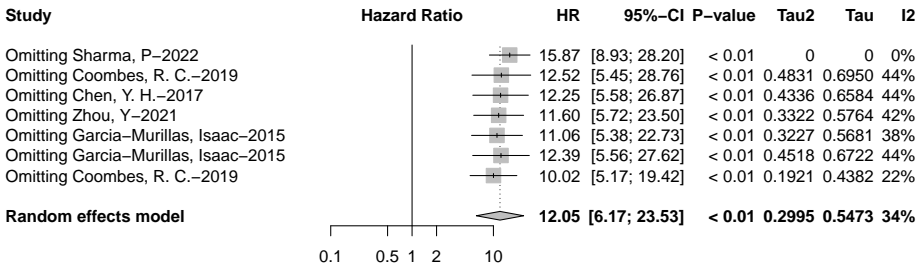

h

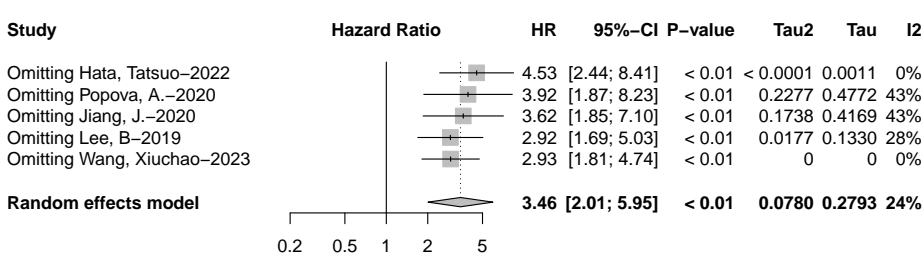

Supplement: Figure S16 [file mmc28.pdf]

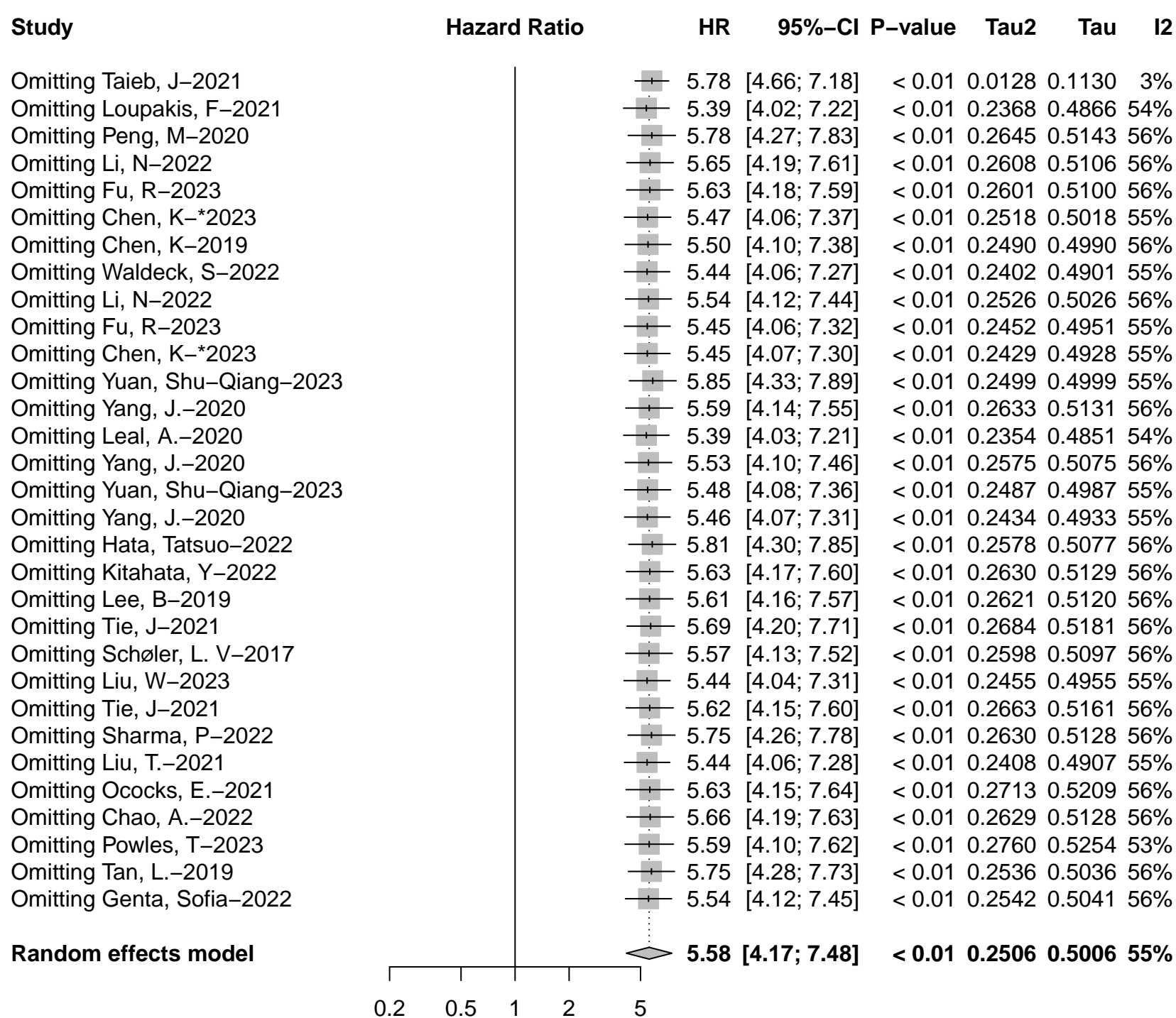

Figure S18 The results of sensitivity analysis of HR of OS (Univariate analysis) of pan-cancer

Supplement: Figure S18 [file mmc30.pdf]

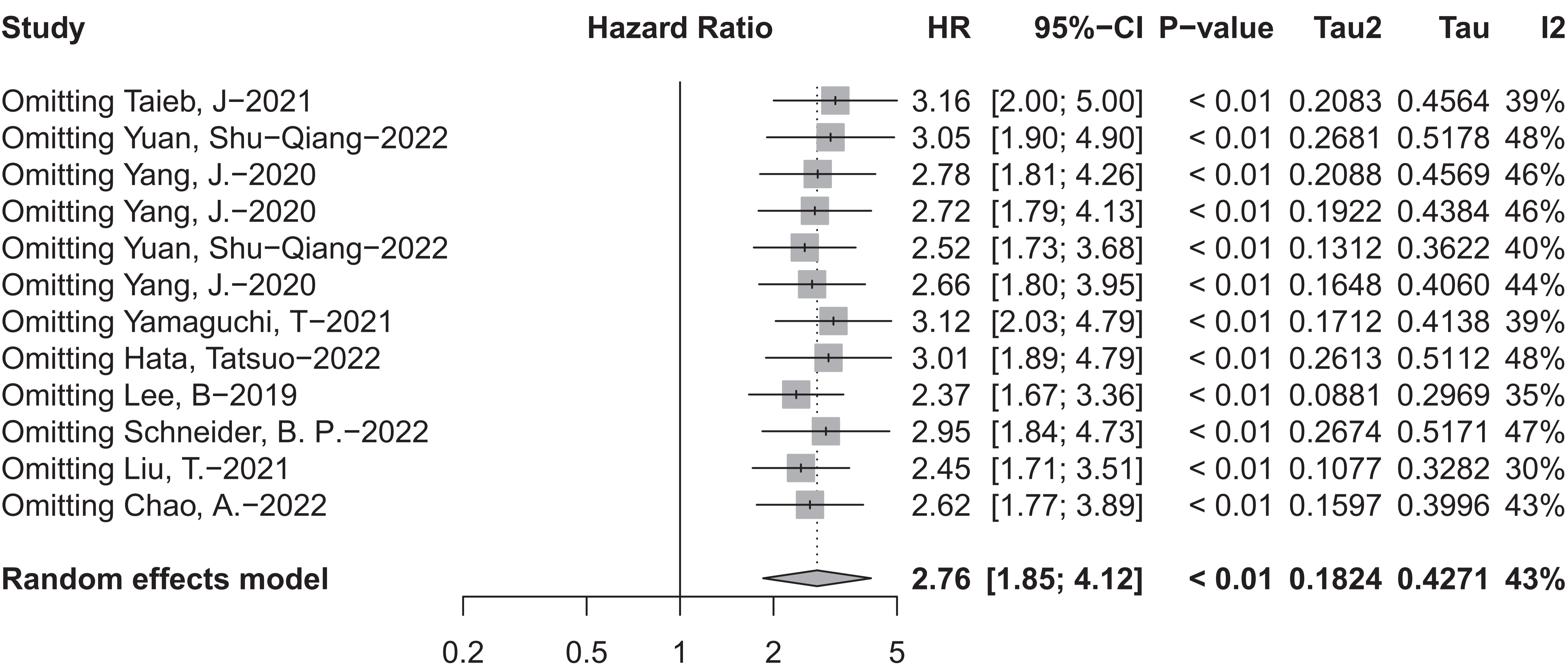

Figure S20 The results of sensitivity analysis of HR of OS (Multivariable analysis) of pan-cancer

Supplement: Figure S20 [file mmc32.pdf]
