# Supplementary material for: Circulating tumour DNA-Based molecular residual disease detection in resectable cancers: a systematic review and meta-analysis
Source: eBioMedicine. 2024 Apr 13;103:105109. doi: 10.1016/j.ebiom.2024.105109 (PMC11021841; doi:10.1016/j.ebiom.2024.105109)
Supplement: Figure S2 [file mmc14.pdf]

Source	Time	Sex (female/male)	N of event	Detection	Adj	Positive	Negative	HR	95% CI
**CRC**									
Taieb, J−2021	1	441/576	1017 (184)	42d	——	140	877	1.56	[1.08; 2.26]
Loupakis, F−2021	1	40/72	112 (——)	27d	yes	61	51	16.00	[3.90; 68.00]
Total (common effect)								1.80	[1.26; 2.58]
Total (random effect)								4.49	[0.46; 43.54]
Heterogeneity: χ^2_1 = 9.55 (P = .002), I^2 = 90%									
**NSCLC**									
Li, N−2022	1	——/——	116 (——)	1m	——	12	104	4.04	[0.74; 22.09]
Fu, R−2023	1	——/——	146 (——)	1m (+−7d)	——	36	110	4.43	[0.76; 25.66]
Chen, K−*2023	1	——/——	156 (——)	1m	——	19	137	9.50	[3.04; 29.67]
Chen, K−2019	1	——/——	25 (——)	3d	——	7	18	14.22	[1.58; 128.15]
Waldeck, S−2022	1	——/——	16 (——)	1w to 2w	——	4	12	33.33	[3.04; 365.32]
Peng, M−2020	2	20/51	71 (25)	——	——	30	41	3.22	[1.35; 7.71]
Li, N−2022	2	49/70	119 (6)	3m to 6m/follow−up	——	37	82	9.99	[1.17; 85.78]
Fu, R−2023	2	81/96	177 (——)	3m to 6m/follow−up	——	55	122	13.77	[2.75; 68.95]
Chen, K−*2023	2	56/54	110 (——)	——	——	23	87	21.98	[2.57; 188.25]
Total (common effect)								6.79	[4.11; 11.23]
Total (random effect)								7.40	[4.14; 13.23]
Heterogeneity: χ^2_8 = 7.87 (P = .45), I^2 = 0%									
**GC**									
Yuan, Shu−Qiang−2022	1	34/68	100 (——)	4d (1~7d)	——	25	75	2.53	[1.17; 5.45]
Yang, J.−2020	1	——/——	38 (——)	1m	——	7	31	6.22	[1.86; 20.78]
Leal, A.−2020	1	——/——	50 (——)	6.5w	yes	9	11	21.80	[3.90; 123.10]
Yang, J.−2020	2	——/——	44 (13)	3m/1y−6m/follow−up	——	17	27	7.95	[2.17; 29.14]
Yuan, Shu−Qiang−2022	3	15/26	41 (——)	——	——	10	31	11.88	[2.38; 59.24]
Yang, J.−2020	3	——/——	23 (——)	——	——	5	18	22.23	[2.45; 201.86]
Total (common effect)								5.55	[3.35; 9.22]
Total (random effect)								7.30	[3.45; 15.44]
Heterogeneity: χ^2_5 = 9.13 (P = .10), I^2 = 45%									
**PAAD**									
Hata, Tatsuo−2022	1	21/45	66 (——)	2w	——	16	50	2.73	[1.11; 6.68]
Kitahata, Y−2022	1	——/——	27 (——)	4w to 8w	——	13	14	5.02	[1.23; 20.52]
Lee, B−2019	1	——/——	35 (——)	4w to 8w	——	13	22	5.50	[1.00; 17.40]
Total (common effect)								3.65	[1.87; 7.12]
Total (random effect)								3.65	[1.87; 7.12]
Heterogeneity: χ^2_2 = 0.92 (P = .63), I^2 = 0%									
**CRLM**									
Tie, J−2021	1	14/35	49 (——)	4w to 10w	——	12	37	4.20	[1.50; 11.80]
Schøler, L. V−2017	1	6/15	21 (——)	3m	——	6	15	6.70	[1.60; 28.70]
Liu, W−2023	1	——/——	134 (——)	31d	——	42	92	11.50	[3.28; 40.60]
Tie, J−2021	3	——/——	45 (——)	——	——	11	34	5.54	[1.83; 16.80]
Total (common effect)								6.14	[3.40; 11.08]
Total (random effect)								6.14	[3.40; 11.08]
Heterogeneity: χ^2_3 = 1.52 (P = .68), I^2 = 0%									
**BC**									
Sharma, P−2022	1	——/——	47 (13)	——	——	16	31	3.05	[1.02; 9.13]
**ESCA**									
Liu, T.−2021	1	——/——	23 (5)	1w	——	4	19	27.60	[2.90; 259.10]
Ococks, E.−2021	2	——/——	63 (——)	——	——	10	53	5.55	[2.42; 12.71]
Total (common effect)								6.73	[3.09; 14.65]
Total (random effect)								8.69	[2.12; 35.62]
Heterogeneity: χ^2_1 = 1.72 (P = .19), I^2 = 42%									
**OV**									
Chao, A.−2022	1	——/——	29 (——)	7d to 10d	——	11	18	4.18	[0.97; 18.06]
**BLCA**									
Powles, T−2023	1	62/221	281 (——)	10w	no	98	183	6.30	[4.30; 9.30]
**melanoma**									
Tan, L.−2019	1	——/——	52 (5)	2w	——	13	39	1.40	[0.23; 9.00]
Genta, Sofia−2022	1	12/33	45 (——)	——	——	4	41	8.90	[1.45; 54.77]
Total (common effect)								3.56	[0.98; 12.94]
Total (random effect)								3.55	[0.58; 21.72]
Heterogeneity: χ^2_1 = 1.97 (P = .16), I^2 = 49%									
Total (common effect)								4.24	[3.54; 5.07]
Total (random effect)								5.58	[4.17; 7.48]

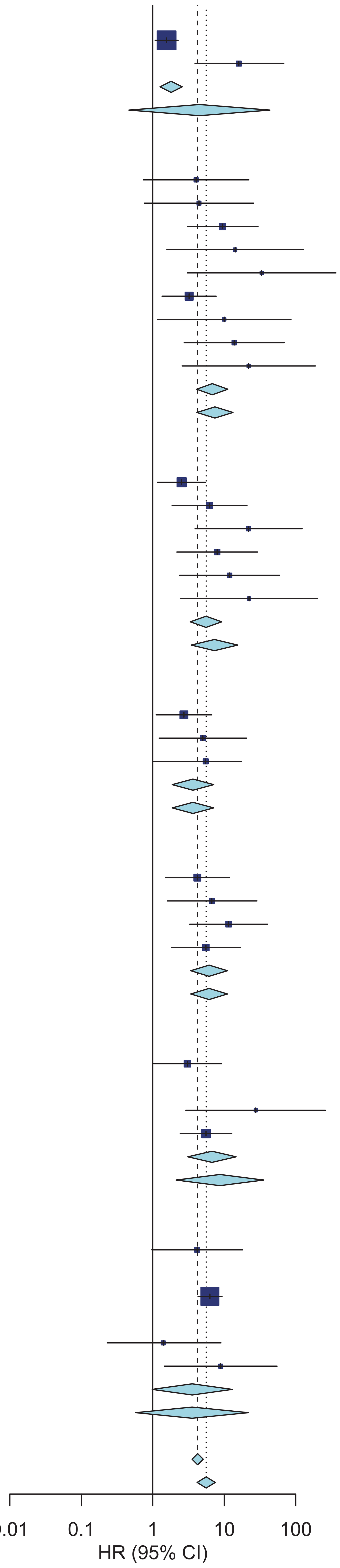

Heterogeneity: χ^2_{30} = 66.62 (P < .001), I^2 = 55%
Test for subgroup differences (common effect): χ^2_9 = 33.92 (P < .001)
Test for subgroup differences (random effects): χ^2_9 = 5.25 (P = .81)

Figure S2 Pooled HR of univariate analysis of OS of CRC, NSCLC, CRLM, PAAD, BLCA, melanoma, BC, GC, OV and ESCA; Negative=ctDNA-; Positive=ctDNA+; 1=landmark detection, 2=longitudinal detection, 3=post-adjuvant therapy; Detection=the time of ctDNA detection after surgery; Adj=adjuvant therapy; d=day; w=week; m=month; y=year; Two arms: Chen, K-*2023; Tie, J-2021; Fu, R-2023; Li, N-2022; Yuan, Shu Qiang-2022; Three arms: Yang, J.-2020. N of event: total sample (sample of recurrence). Solid line is invalid line, and 95% confidence interval crossing is not statistically significant. Vertical dashed lines are pooled HR. I^2 was estimated by Higgins' approach. x^2 was estimated by Q-test.
