# Supplementary material for: Circulating tumour DNA-Based molecular residual disease detection in resectable cancers: a systematic review and meta-analysis
Source: eBioMedicine. 2024 Apr 13;103:105109. doi: 10.1016/j.ebiom.2024.105109 (PMC11021841; doi:10.1016/j.ebiom.2024.105109)
Supplement: Figure S3 [file mmc15.pdf]

Source	Time	Sex (female/male)	N of event	Detection	Adj	Positive	Negative	HR	95% CI
**CRC**									
Taieb, J−2021	1	441/576	1017 (298)	42d	——	140	877	1.55	[1.13; 2.12]
Benhaim, L.−2021	1	73/98	171 (29)	5d	——	18	153	3.22	[1.32; 7.89]
Reinert, T−2019	1	——/——	94 (17)	30d	——	10	84	4.50	[1.60; 12.80]
Li,Y−2022	1	——/——	151 (39)	30d	——	24	127	5.54	[2.49; 12.33]
Loupakis, F−2021	1	40/72	112 (82)	27d	yes	61	51	5.78	[3.34; 10.00]
Tie, J−2019	1	52/107	159 (23)	4w to 10w	all	19	140	6.00	[2.20; 15.00]
McNamara, S−2022	1	——/——	84 (9)	7d(1~34d)	——	8	76	7.10	[2.02; 24.89]
Chen, G−2021	1	106/134	240 (23)	3d to 7d	——	20	220	8.02	[3.59; 17.92]
Kotani, D−2023	1	489/550	1039 (196)	4w	——	187	852	10.82	[7.07; 16.60]
Tarazona, N−2019	1	——/——	69 (18)	6w to 8w	——	14	55	11.64	[3.67; 36.88]
Watanabe, Jun−2023	1	——/——	2083 (——)	1m	——	286	1797	11.68	[8.61; 15.85]
Tie, J−2016	1	99/131	230 (——)	4w to 10w	all	20	210	14.00	[6.80; 28.00]
Mo, S.−2023	1	——/——	255 (50)	1m	——	59	196	17.00	[8.20; 34.00]
Tie, J−2016	1	——/——	178 (27)	4w to 10w	no	14	164	28.00	[11.00; 68.00]
Anandappa, Gayathri−2021	1	——/——	64 (14)	——	——	11	53	28.80	[3.50; 234.10]
Henriksen, T. V−2022	1	65/95	140 (38)	2w	——	20	120	30.97	[10.63; 90.20]
Tarazona, N−2019	2	33/61	94 (18)	4m	——	32	62	11.12	[2.53; 55.65]
Chen, G−2021	2	——/——	125 (——)	6m/once−3m/follow−up	——	25	100	23.00	[6.30; 69.97]
Reinert, T−2019	2	——/——	75 (16)	——	——	15	60	39.90	[7.50; 211.00]
Henriksen, T. V−2022	2	——/——	114 (24)	——	——	22	92	40.70	[11.60; 143.00]
Li,Y−2022	3	——/——	124 (36)	——	yes	11	113	3.27	[1.02; 9.99]
Reinert, T−2019	3	13/45	58 (14)	——	yes	7	51	11.80	[3.40; 40.80]
Chen, G−2021	3	——/——	137 (——)	——	yes	12	125	20.79	[6.66; 64.87]
Henriksen, T. V−2022	3	——/——	93 (20)	——	——	10	83	94.25	[15.74; 564.30]
Total (common effect)								7.19	[6.23; 8.30]
Total (random effect)								10.54	[7.25; 15.32]
Heterogeneity: $\chi^2_{23} = 165.81$ ($P < .001$), $I^2 = 86\%$									
**NSCLC**									
Qiu, B−2021	1	——/——	85 (33)	30d	——	18	67	3.50	[1.70; 7.40]
Wang, S−2022	1	——/——	114 (——)	3m	——	16	98	4.17	[1.80; 9.70]
Wang, S−2022	1	——/——	89 (——)	6m	——	11	78	4.59	[1.68; 12.50]
Wang, S−2022	1	——/——	116 (——)	7d	——	16	100	5.49	[1.86; 16.17]
Xia, L−2022	1	——/——	329 (70)	3d to 1m	——	26	303	8.60	[4.80; 15.30]
Chen, K−*2023	1	——/——	156 (——)	1m	——	19	137	8.86	[3.72; 21.10]
Qiu, B−2021	2	——/——	89 (34)	3m/follow−up	——	35	54	6.70	[2.80; 16.00]
Wang, S−2022	2	——/——	117 (34)	3m/follow−up	——	40	77	8.33	[3.59; 19.30]
Zhang, J. T.−2022	2	——/——	236 (47)	3m to 6m/follow−up	——	46	190	34.04	[13.87; 83.54]
Chen, K−*2023	2	——/——	110 (19)	——	——	23	87	47.20	[11.27; 197.60]
Qiu, B−2021	3	——/——	64 (——)	——	yes	8	56	4.40	[1.60; 12.00]
Total (common effect)								7.44	[5.74; 9.65]
Total (random effect)								7.72	[4.98; 11.97]
Heterogeneity: $\chi^2_{10} = 26.03$ ($P = .004$), $I^2 = 62\%$									
**GC**									
Yuan, Shu−Qiang−2023	1	34/68	100 (33)	4d (1~7d)	——	25	75	2.48	[1.24; 4.97]
Yang, J.−2020	1	——/——	38 (17)	1m	——	7	31	6.13	[1.74; 21.64]
Fedyanin, M.−2020	1	——/——	42 (12)	7d (5−15d)	——	10	32	6.60	[1.50; 30.00]
Yang, J.−2020	2	——/——	44 (36)	3m/1y−6m/follow−up	——	17	27	14.93	[3.40; 65.59]
Yang,J.−2020	3	——/——	23 (——)	——	——	5	18	4.73	[0.72; 31.16]
Yuan, Shu−Qiang−2023	3	15/26	41 (24)	——	——	10	31	10.31	[2.04; 52.03]
Total (common effect)								4.51	[2.78; 7.31]
Total (random effect)								5.48	[2.83; 10.62]
Heterogeneity: $\chi^2_{5} = 6.84$ ($P = .23$), $I^2 = 27\%$									
**PAAD**									
Yamaguchi, T−2021	1	54/43	97 (——)	3d	——	27	70	1.61	[0.90; 2.77]
Hata, Tatsuo−2022	1	21/45	66 (——)	2w	——	16	50	2.67	[1.26; 5.69]
Jiang, J.−2020	1	10/17	27 (14)	7d	——	9	18	3.60	[1.15; 11.28]
Lee, B−2019	1	14/21	35 (23)	4w to 8w	——	13	22	6.30	[2.40; 16.20]
Total (common effect)								2.51	[1.71; 3.68]
Total (random effect)								2.85	[1.57; 5.19]
Heterogeneity: $\chi^2_{3} = 6.37$ ($P = .09$), $I^2 = 53\%$									
**CRLM**									
Liu, W−2023	1	36/98	134 (84)	31d	——	42	92	1.89	[1.14; 3.11]
Nishioka, Y.−2022	1	42/63	105 (66)	——	——	32	73	2.04	[1.18; 3.52]
Tie, J−2021	1	14/35	49 (21)	4w to 10w	——	12	37	3.13	[1.00; 9.82]
Bolhuis, K.−2021	1	8/15	23 (17)	3m	yes	6	17	4.10	[1.19; 14.47]
Reinert, T−2022	1	——/——	40 (28)	30d	——	13	27	10.80	[3.60; 32.70]
Reinert, T−2022	2	——/——	67 (47)	3m/follow−up	——	34	33	4.90	[2.50; 10.00]
Wang, D. S−2022	3	19/30	49 (12)	——	——	22	27	2.40	[1.12; 5.15]
Total (common effect)								2.74	[2.08; 3.60]
Total (random effect)								3.10	[2.02; 4.77]
Heterogeneity: $\chi^2_{6} = 12.43$ ($P = .05$), $I^2 = 52\%$									
**BC**									
Schneider, B. P.−2022	1	——/——	146 (——)	14d	——	93	53	1.93	[1.05; 3.52]
Chen, Y. H.−2017	1	——/——	33 (13)	——	——	4	29	8.60	[1.60; 45.70]
Garcia−Murillas, Isaac−2015	1	37/0	37 (12)	2w to 4w	——	7	30	21.10	[2.50; 177.47]
Garcia−Murillas, Isaac−2015	2	43/0	43 (15)	6m/follow−up	——	13	30	9.60	[2.40; 38.90]
Total (common effect)								3.16	[1.89; 5.27]
Total (random effect)								5.91	[1.93; 18.04]
Heterogeneity: $\chi^2_{3} = 9.42$ ($P = .02$), $I^2 = 68\%$									
**ESCA**									
Liu, T.−2021	1	——/——	23 (5)	1w	——	4	19	184.60	[3.60; 9576.90]
Ococks, E.−2021	2	——/——	63 (26)	——	——	10	53	4.77	[1.93; 11.80]
Total (common effect)								5.73	[2.37; 13.84]
Total (random effect)								17.56	[0.57; 543.47]
Heterogeneity: $\chi^2_{1} = 3.14$ ($P = .08$), $I^2 = 68\%$									
**OV**									
Chao, A.−2022	1	——/——	29 (8)	7d to 10d	——	11	18	8.41	[2.49; 28.39]
**BLCA**									
Carrasco, R−2022	1	——/——	34 (16)	4m	——	12	22	4.20	[1.08; 16.28]
Powles, T−2021	1	62/221	281 (——)	10w	no	98	183	6.19	[4.29; 8.91]
Christensen, Emil−2019	2	——/——	64 (13)	——	——	17	47	129.60	[12.60; 17871.10]
Total (common effect)								6.21	[4.37; 8.82]
Total (random effect)								6.21	[4.37; 8.82]
Heterogeneity: $\chi^2_{2} = 3.01$ ($P = .22$), $I^2 = 34\%$									
**melanoma**									
Tan, L.−2019	1	——/——	52 (29)	2w	——	13	39	11.00	[3.70; 31.50]
**HCC**									
Zhao, L−2022	2	——/——	59 (27)	——	——	21	38	8.50	[2.91; 24.86]
Total (common effect)								5.62	[5.09; 6.20]
Total (random effect)								7.07	[5.66; 8.83]

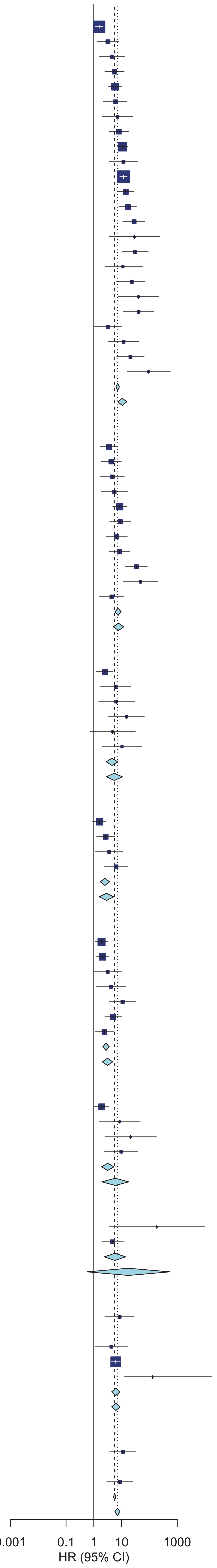

Heterogeneity: $\chi^2_{63} = 300.75$ ($P < .001$), $I^2 = 79\%$
Test for subgroup differences (common effect): $\chi^2_{10} = 67.69$ ($P < .001$)
Test for subgroup differences (random effects): $\chi^2_{10} = 27.24$ ($P = .002$)

Figure S3 Multivariable analysis: Pooled HR of recurrence of CRC, NSCLC, CRLM, PAAD, BLCA, melanoma, BC, GC, HCC, OV and ESCA; Negative=ctDNA-; Positive=ctDNA+; 1=landmark detection, 2=longitudinal detection, 3=post-adjuvant therapy; re/de=the outcome of recurrence or death; re=the outcome of recurrence; Detection=the time of ctDNA detection after surgery; Adj=adjuvant therapy; d=day; w=week; m=month; y=year; Two arms: Li,Y-2022; Tarazona, N-2019; Chen, K-*2023; Reinert, T-2022; Garcia Murillas, Isaac-2015; Tie, J-2016; Yuan, Shu-Qiang-2023; Three arms: Henriksen, T. V-2022, Chen, G-2021; Qiu, B-2021; Yang, J.-2020; Reinert, T-2019; Four arms: Wang, S-2022. N of event: total sample (sample of recurrence). Solid line is invalid line, and 95% confidence interval crossing is not statistically significant. Vertical dashed lines are pooled HR. I^2 was estimated by Higgins' approach. x^2 was estimated by Q-test.
