# Supplementary material for: Circulating tumour DNA-Based molecular residual disease detection in resectable cancers: a systematic review and meta-analysis
Source: eBioMedicine. 2024 Apr 13;103:105109. doi: 10.1016/j.ebiom.2024.105109 (PMC11021841; doi:10.1016/j.ebiom.2024.105109)
Supplement: Figure S4 [file mmc16.pdf]

Source	Time	Sex (female/male)	N of event	Detection	Adj	Positive	Negative	HR	95% CI
**CRC**									
Taieb, J−2021	1	441/576	1017 (298)	42d	——	140	877	1.65	[1.12; 2.43]
**GC**									
Yuan, Shu−Qiang−2023	1	34/68	100 (——)	4d (1~7d)	——	25	75	2.05	[0.93; 4.55]
Yang, J.−2020	1	——/——	38 (——)	1m	——	7	31	3.45	[0.85; 13.89]
Yang, J.−2020	2	——/——	44 (13)	3m/1y−6m/follow−up	——	17	27	4.07	[0.96; 17.30]
Yuan, Shu−Qiang−2023	3	15/26	41 (——)	——	——	10	31	8.11	[1.55; 42.27]
Yang, J.−2020	3	——/——	23 (——)	——	——	5	18	12.46	[0.60; 260.33]
Total (common effect)								3.13	[1.77; 5.55]
Total (random effect)								3.25	[1.76; 6.02]
Heterogeneity: $\chi^2_4 = 3.31$ (P = .51), I^2 = 0%									
**PAAD**									
Yamaguchi, T−2021	1	54/43	97 (——)	3d	——	27	70	1.36	[0.75; 2.39]
Hata, Tatsuo−2022	1	21/45	66 (——)	2w	——	16	50	2.12	[0.83; 5.45]
Lee, B−2019	1	14/21	35 (——)	4w to 8w	——	13	22	7.50	[2.10; 27.70]
Total (common effect)								1.88	[1.19; 2.98]
Total (random effect)								2.42	[0.97; 6.02]
Heterogeneity: $\chi^2_2 = 5.69$ (P = .06), I^2 = 65%									
**BC**									
Schneider, B. P.−2022	1	——/——	146 (——)	14d	——	93	53	2.64	[1.18; 5.91]
**ESCA**									
Liu, T.−2021	1	——/——	23 (5)	1w	——	4	19	25.80	[2.70; 242.60]
**OV**									
Chao, A.−2022	1	——/——	29 (8)	7d to 10d	——	11	18	6.56	[1.07; 40.17]
Total (common effect)								2.14	[1.67; 2.73]
Total (random effect)								2.76	[1.85; 4.12]

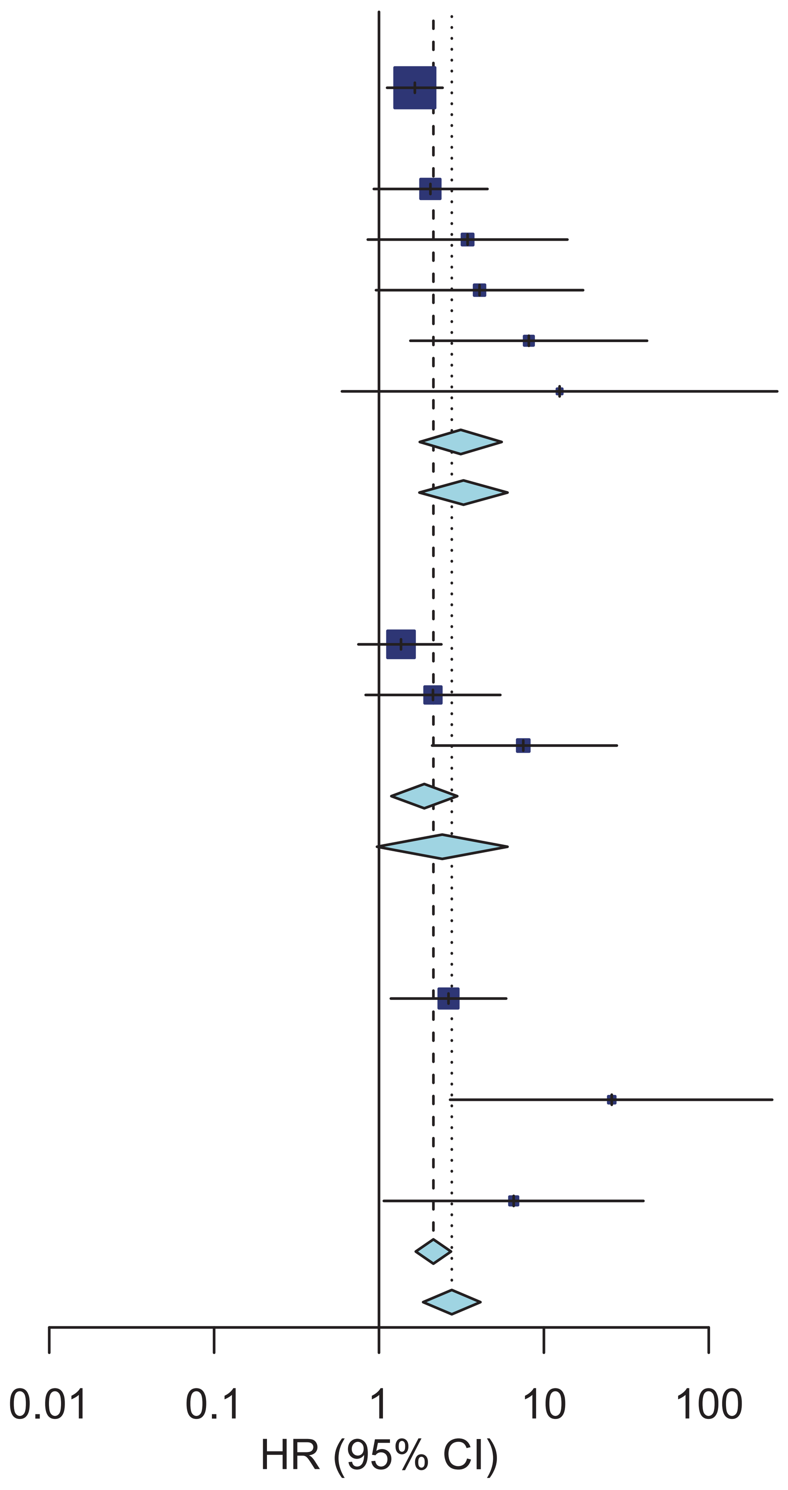

Heterogeneity: $\chi^2_{11} = 19.16$ (P = .06), I^2 = 43%
Test for subgroup differences (common effect): $\chi^2_5 = 10.17$ (P = .07)
Test for subgroup differences (random effects): $\chi^2_5 = 9.88$ (P = .08)

Figure S4 Multivariable analysis: Pooled HR of OS of CRC, GC, PAAD, BC, ESCA, OV; Negative=ctDNA-; Positive=ctDNA+; 1=landmark detection, 2=longitudinal detection, 3=post-adjuvant therapy; Detection=the time of ctDNA detection after surgery; Adj=adjuvant therapy; d=day; w=week; m=month; y=year; Two arms: Yuan, Shu-Qiang-2023; Three arms: Yang, J.-2020; N of event: total sample (sample of recurrence). Solid line is invalid line, and 95% confidence interval crossing is not statistically significant. Vertical dashed lines are pooled HR. I^2 was estimated by Higgins' approach. x^2 was estimated by Q-test.
