# Supplementary material for: Circulating tumour DNA-Based molecular residual disease detection in resectable cancers: a systematic review and meta-analysis
Source: eBioMedicine. 2024 Apr 13;103:105109. doi: 10.1016/j.ebiom.2024.105109 (PMC11021841; doi:10.1016/j.ebiom.2024.105109)
Supplement: Figure S5 [file mmc17.pdf]

Source	Time	Sex (female/male)	N of event	Detection	Adj	Positive	Negative	HR	95% CI
1									
Li, N−2022	1	——/——	116 (——)	1m	——	12	104	3.04	[1.22; 7.58]
Kuang, P. P.−2020	1	——/——	35 (9)	2w	——	8	27	3.69	[1.11; 12.25]
Wang, S−2022	1	——/——	116 (——)	7d	——	16	100	3.90	[1.85; 8.20]
Qiu, B−2021	1	——/——	85 (33)	30d	——	18	67	3.95	[1.96; 7.96]
Chen, K−2023	1	——/——	148 (——)	3d to 1m	——	23	125	4.13	[1.90; 9.00]
Wang, S−2022	1	——/——	114 (——)	3m	——	16	98	4.32	[2.06; 9.08]
Fu, R−2023	1	——/——	146 (38)	1m (+−7d)	——	36	110	5.07	[2.33; 11.01]
Wang, S−2022	1	——/——	89 (——)	6m	——	11	78	6.19	[2.44; 15.69]
Chen, K−2019	1	——/——	25 (9)	3d	——	7	18	7.55	[2.09; 27.27]
Waldeck, S−2022	1	——/——	16 (8)	1w to 2w	——	4	12	10.64	[1.65; 68.76]
Xia, L−2022	1	——/——	329 (70)	3d to 1m	——	26	303	11.10	[6.50; 19.00]
Zhang, J. T.−2022	1	——/——	245 (47)	1m(±7d)	——	21	224	12.50	[3.08; 50.60]
Chen, K−*2023	1	——/——	156 (——)	1m	——	19	137	16.40	[7.84; 34.31]
Total (common effect)								6.14	[4.87; 7.73]
Total (random effect)								5.95	[4.30; 8.23]
Heterogeneity: χ^2_{12} = 20.93 (P = .05), I^2 = 43%									
2									
Peng, M−2020	2	20/51	71 (31)	——	——	30	41	3.11	[1.47; 6.55]
Li, N−2022	2	49/70	119 (28)	3m to 6m/follow−up	——	37	82	3.46	[1.59; 7.55]
Fu, R−2023	2	81/96	177 (41)	3m to 6m/follow−up	——	55	122	7.14	[3.60; 14.15]
Wang, S−2022	2	——/——	117 (34)	3m/follow−up	——	40	77	7.59	[3.53; 16.32]
Yang, W.−2020	2	49/33	82 (5)	3m/1y−6m/2~3y	——	15	67	8.50	[1.30; 56.30]
Qiu, B−2021	2	——/——	89 (34)	3m/follow−up	——	35	54	8.55	[3.70; 19.74]
Chen, K−2023	2	——/——	63 (——)	——	——	8	55	15.14	[4.45; 51.45]
Tan, A.−2021	2	23/34	57 (——)	——	——	8	49	22.00	[3.60; 133.80]
Chen, K−*2023	2	56/54	110 (19)	——	——	23	87	37.42	[10.76; 130.16]
Zhang, J. T.−2022	2	——/——	236 (47)	3m to 6m/follow−up	——	46	190	50.00	[25.00; 100.00]
Total (common effect)								9.55	[7.21; 12.64]
Total (random effect)								10.42	[5.58; 19.45]
Heterogeneity: χ^2_9 = 44.23 (P < .001), I^2 = 80%									
3									
Qiu, B−2021	3	——/——	64 (——)	——	Yes	8	56	3.22	[1.26; 8.18]
Kuang, P. P.−2020	3	——/——	36 (9)	——	Yes	8	28	8.76	[1.63; 47.01]
Total (common effect)								4.08	[1.80; 9.24]
Total (random effect)								4.12	[1.77; 9.60]
Heterogeneity: χ^2_1 = 1.04 (P = .31), I^2 = 4%									
Total (common effect)								7.14	[6.00; 8.50]
Total (random effect)								7.23	[5.27; 9.93]

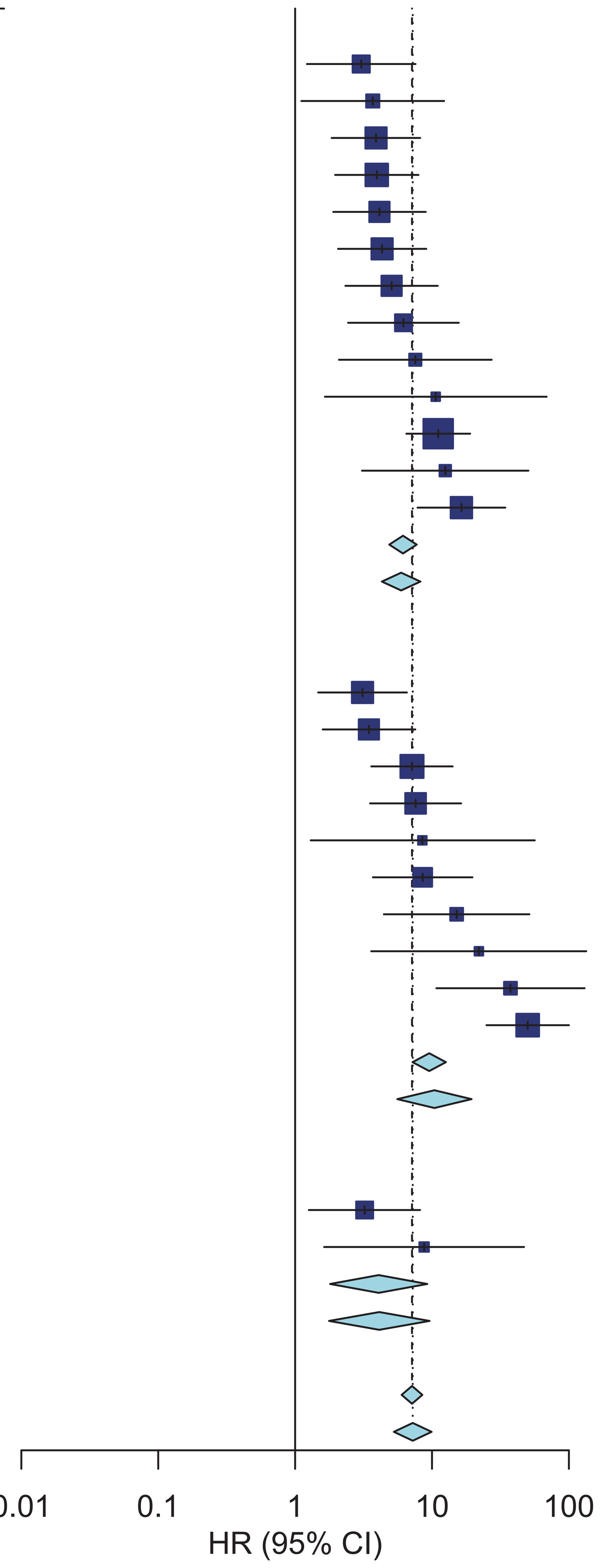

Heterogeneity: χ^2_{24} = 73.76 (P < .001), I^2 = 67%
Test for subgroup differences (common effect): χ^2_2 = 7.56 (P = .02)
Test for subgroup differences (random effects): χ^2_2 = 3.58 (P = .17)

Figure S5 Subgroup for pooled HR of univariate analysis of NSCLC recurrence monitoring; 1=landmark detection, 2=longitudinal detection, 3=post-adjuvant therapy; Negative=ctDNA-; Positive=ctDNA+; Detection=the time of ctDNA detection after surgery; Adj=adjuvant therapy; d=day; w=week; m=month; y=year; Two arms: Chen, K-2023; Chen, K-*2023; Fu, R-2023; Kuang, P. P.-2020; Li, N-2022; Zhang, J. T.-2022; Three arms: Qiu, B-2021; Four arms: Wang, S-2022. N of event: total sample (sample of recurrence). Solid line is invalid line, and 95% confidence interval crossing is not statistically significant. Vertical dashed lines are pooled HR. I^2 was estimated by Higgins' approach. x^2 was estimated by Q-test.
