# Supplementary material for: Circulating tumour DNA-Based molecular residual disease detection in resectable cancers: a systematic review and meta-analysis
Source: eBioMedicine. 2024 Apr 13;103:105109. doi: 10.1016/j.ebiom.2024.105109 (PMC11021841; doi:10.1016/j.ebiom.2024.105109)
Supplement: Figure S6 [file mmc18.pdf]

Source	Time	Sex (female/male)	N of event	Detection	Adj	Positive	Negative	HR	95% CI
1									
Taieb, J−2021	1	441/576	1017 (298)	42d	——	140	877	1.46	[1.08; 1.97]
Tie, J−2022	1	——/——	291 (23)	4w or 7w−re/de	——	45	246	1.83	[0.79; 4.27]
Tie, J−2022	1	——/——	291 (22)	4w or 7w−re	——	45	246	2.45	[1.00; 5.99]
Li,Y−2022	1	——/——	151 (39)	30d	——	24	127	5.31	[2.56; 10.99]
Loupakis, F−2021	1	40/72	112 (82)	27d	Yes	61	51	5.80	[3.50; 9.70]
Loupakis, F−2021	1	——/——	47 (35)	27d	No	30	17	15.00	[4.30; 49.00]
Tarazona, N−2019	1	——/——	69 (18)	6w to 8w	——	14	55	6.96	[2.57; 18.91]
Henriksen, T. V−2022	1	——/——	140 (38)	2w	——	20	120	7.00	[3.70; 13.50]
Reinert, T−2019	1	——/——	94 (17)	30d	——	10	84	7.20	[2.70; 19.00]
Bryant,C −2021	1	——/——	45 (27)	3w	——	25	20	7.70	[2.60; 22.50]
Han, S. W.−2022	1	——/——	71 (23)	3w	——	13	58	8.23	[3.30; 21.00]
Anandappa, Gayathri−2021	1	——/——	64 (14)	——	——	11	53	9.00	[2.60; 32.00]
Kotani, D−2023	1	489/550	1039 (196)	4w	——	187	852	10.00	[7.70; 14.00]
Hofste, Lisa S. M.−2023	1	——/——	19 (5)	1w to 2w	——	3	16	10.90	[1.10; 106.70]
Chen, G−2021	1	106/134	240 (23)	3d to 7d	——	20	220	10.98	[5.31; 22.72]
Tie, J−2019	1	52/107	159 (23)	4w to 10w	All	19	140	13.00	[5.50; 31.00]
Tie, J−2019	1	——/——	102 (——)	4w to 10w	Yes	11	91	10.00	[3.40; 29.00]
Tie, J−2019	1	——/——	57 (——)	4w to 10w	No	8	49	22.00	[4.20; 110.00]
Tie, J−2016	1	99/131	230 (——)	4w to 10w	All	20	210	13.00	[6.60; 27.00]
Tie, J−2016	1	——/——	44 (——)	4w to 10w	Yes	3	41	11.00	[1.80; 68.00]
Tie, J−2016	1	——/——	178 (27)	4w to 10w	No	14	164	18.00	[7.90; 40.00]
Watanabe, Jun−2023	1	——/——	2083 (——)	1m	——	286	1797	12.00	[9.10; 15.00]
Zhou, Jian−2022	1	——/——	40 (——)	8d	——	6	34	16.57	[3.01; 91.36]
Mo, S.−2023	1	——/——	255 (50)	1m	——	59	196	17.50	[8.90; 34.40]
Total (common effect)								6.94	[6.13; 7.85]
Total (random effect)								7.88	[5.75; 10.79]
Heterogeneity: χ^2_{23} = 168.06 (P < .001), I^2 = 86%									
2									
Tarazona, N−2019	2	33/61	94 (18)	4m/follow−up	——	32	62	8.03	[1.79; 35.98]
Henriksen, T. V−2022	2	——/——	114 (24)	——	——	22	92	50.80	[14.90; 172.00]
Mo, S.−2023	2	——/——	226 (——)	3m/follow−up	——	47	179	13.50	[5.40; 33.80]
Chen, G−2021	2	.	137 (——)	6m/once−3m/follow−up	——	25	100	32.02	[10.79; 95.08]
Reinert, T−2019	2	——/——	75 (16)	3m/follow−up	——	15	60	43.50	[9.80; 193.50]
Total (common effect)								22.97	[13.55; 38.91]
Total (random effect)								23.49	[12.34; 44.70]
Heterogeneity: χ^2_4 = 5.86 (P = .21), I^2 = 32%									
3									
Li,Y−2022	3	——/——	124 (36)	——	Yes	11	113	2.75	[1.06; 7.13]
Tarazona, N−2019	3	——/——	25 (10)	——	Yes	7	18	10.02	[9.20; 307.30]
Chen, G−2021	3	——/——	137 (——)	——	Yes	12	125	12.76	[5.39; 30.19]
Mo, S.−2023	3	——/——	149 (22)	——	Yes	18	131	13.80	[5.90; 32.10]
Reinert, T−2019	3	13/45	58 (14)	——	Yes	7	51	17.50	[5.40; 56.50]
Henriksen, T. V−2022	3	——/——	93 (20)	——	Yes	10	83	50.76	[15.40; 167.00]
Total (common effect)								11.76	[7.71; 17.95]
Total (random effect)								12.47	[5.73; 27.12]
Heterogeneity: χ^2_5 = 15.35 (P = .009), I^2 = 67%									
Total (common effect)								7.65	[6.81; 8.58]
Total (random effect)								9.74	[7.31; 12.98]

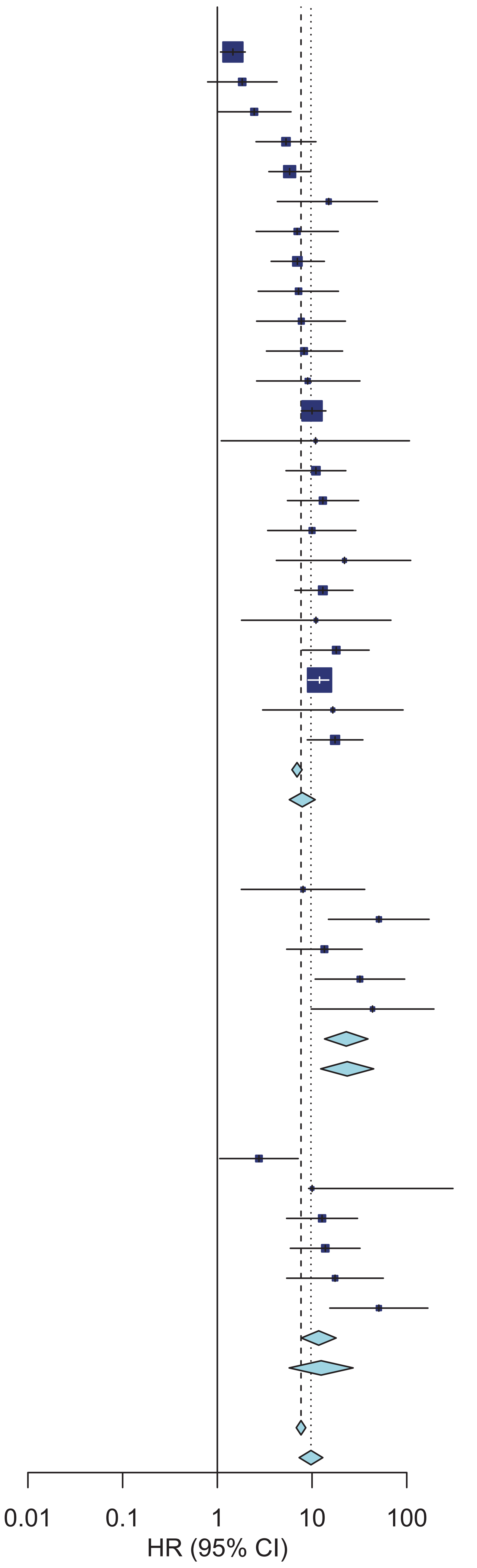

Heterogeneity: χ^2_{34} = 212.34 (P < .001), I^2 = 84%
Test for subgroup differences (common effect): χ^2_2 = 23.07 (P < .001)
Test for subgroup differences (random effects): χ^2_2 = 9.28 (P = .010)

Figure S6 Subgroup for pooled HR of univariate analysis of CRC recurrence monitoring time; 1=landmark detection, 2=longitudinal detection, 3=post-adjuvant therapy; Negative=ctDNA-; Positive=ctDNA+; Detection=the time of ctDNA detection after surgery; Adj=adjuvant therapy; d=day; w=week; m=month; y=year; Two arms: Tie, J-2022; Li,Y-2022; Loupakis, F- 2021; Three arms: Henriksen, T. V-2022, Tie, J-2019, Tie, J-2016, Mo, S.-2023, Chen, G- 2021; Tarazona, N-2019; Reinert, T-2019. N of event: total sample (sample of recurrence). Solid line is invalid line, and 95% confidence interval crossing is not statistically significant. Vertical dashed lines are pooled HR. I^2 was estimated by Higgins' approach. x^2 was estimated by Q-test.
