# Supplementary material for: Circulating tumour DNA-Based molecular residual disease detection in resectable cancers: a systematic review and meta-analysis
Source: eBioMedicine. 2024 Apr 13;103:105109. doi: 10.1016/j.ebiom.2024.105109 (PMC11021841; doi:10.1016/j.ebiom.2024.105109)
Supplement: Figure S7 [file mmc19.pdf]

Source	Time	Sex (female/male)	N of event	Detection	Adj	Positive	Negative	HR	95% CI
1									
Sharma, P−2022	1	——/——	47 (14)	——	——	16	31	3.02	[1.01; 9.01]
Coombes, R. C.−2019	1	——/——	49 (18)	——	——	9	40	11.80	[4.30; 32.50]
Chen, Y. H.−2017	1	——/——	33 (13)	——	——	4	29	12.60	[3.06; 52.20]
Zhou, Y−2021	1	——/——	32 (7)	3d to 7d	Yes	5	27	23.53	[1.90; 290.90]
Garcia−Murillas, Isaac−2015	1	37/0	37 (12)	2w to 4w	——	7	30	25.10	[4.08; 130.50]
Total (common effect)								9.04	[4.98; 16.44]
Total (random effect)								9.90	[4.39; 22.31]
Heterogeneity: $\chi^2_4 = 6.22$ (P = .18), I^2 = 36%									
2									
Garcia−Murillas, Isaac−2015	2	43/0	43 (15)	6m/follow−up	——	13	30	12.00	[3.36; 43.07]
Coombes, R. C.−2019	2	——/——	49 (18)	——	——	16	33	35.80	[8.00; 161.30]
Total (common effect)								18.97	[7.18; 50.15]
Total (random effect)								19.23	[6.66; 55.57]
Heterogeneity: $\chi^2_1 = 1.18$ (P = .28), I^2 = 15%									
Total (common effect)								11.08	[6.66; 18.43]
Total (random effect)								12.05	[6.17; 23.53]

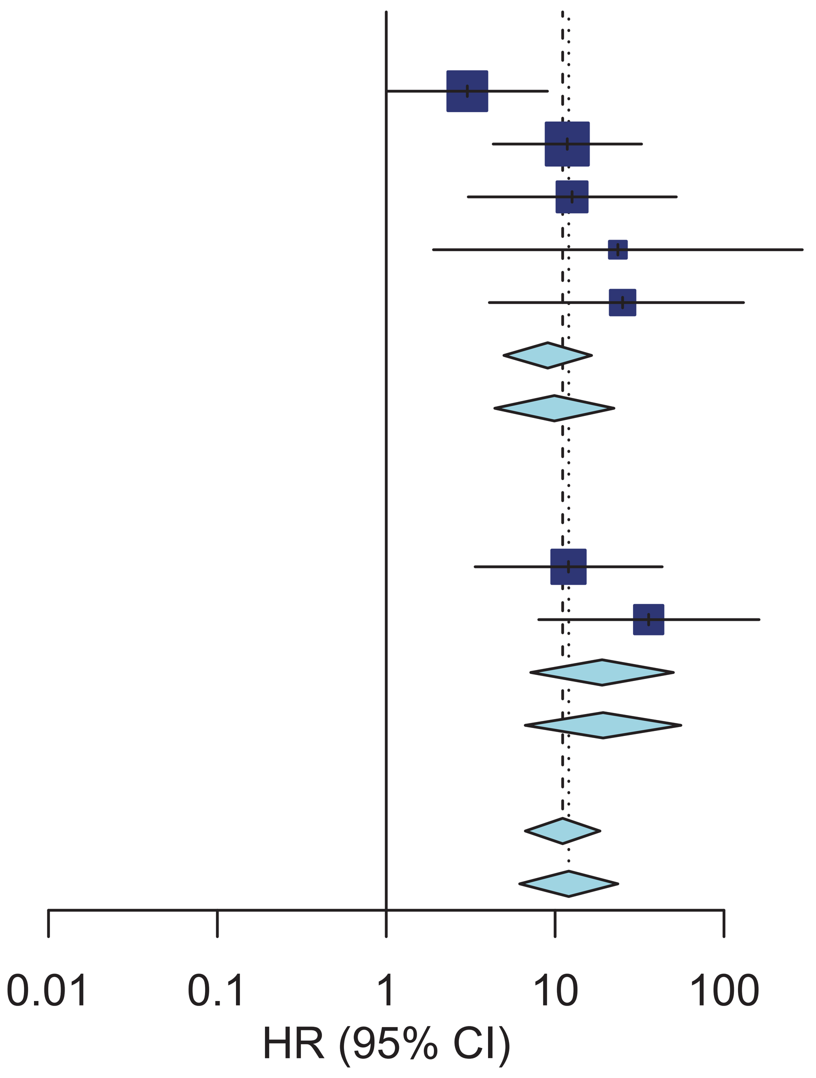

Heterogeneity: $\chi^2_6 = 9.03$ (P = .17), I^2 = 34%
Test for subgroup differences (common effect): $\chi^2_1 = 1.62$ (P = .20)
Test for subgroup differences (random effects): $\chi^2_1 = 0.95$ (P = .33)

Figure S7 Subgroup for pooled HR of univariate analysis of BC recurrence monitoring time; 1=landmark detection, 2=longitudinal detection, Negative=ctDNA-; Positive=ctDNA+; Detection=the time of ctDNA detection after surgery; Adj=adjuvant therapy; d=day; w=week; m=month; y=year; Two arms: Coombes, R. C-2019; Garcia Murillas, Isaac-2015; N of event: total sample (sample of recurrence). Solid line is invalid line, and 95% confidence interval crossing is statistically significant. Vertical dashed lines are pooled HR. I^2 was estimated by Higgins' approach. x^2 was estimated by Q-test.
