# Supplementary material for: Circulating tumour DNA-Based molecular residual disease detection in resectable cancers: a systematic review and meta-analysis
Source: eBioMedicine. 2024 Apr 13;103:105109. doi: 10.1016/j.ebiom.2024.105109 (PMC11021841; doi:10.1016/j.ebiom.2024.105109)
Supplement: Figure S8 [file mmc20.pdf]

Source	Time	Sex (female/male)	N of event	Detection	Adj	Positive	Negative	HR	95% CI
1									
Openshaw, M. R.−2020	1	——/——	22 (13)	——	——	6	16	3.70	[1.15; 11.89]
Takei, Shogo−2023	1	——/——	28 (——)	4w	——	3	25	14.00	[3.00; 62.00]
Morimoto, Y.−2023	1	3/13	16 (7)	1m or 3m	——	6	10	16.90	[1.92; 149.40]
Gerlinger, Marco−2023	1	——/——	24 (——)	——	——	6	18	27.00	[3.00; 241.00]
Liu, T.−2021	1	——/——	23 (5)	1w	——	4	19	27.50	[2.80; 273.10]
Total (common effect)								9.60	[4.54; 20.31]
Total (random effect)								11.08	[4.38; 28.01]
Heterogeneity: $\chi^2_4 = 4.73$ (P = .32), I^2 = 15%									
2									
Ococks, E.−2021	2	——/——	63 (26)	——	——	10	53	5.35	[2.10; 13.63]
Openshaw, M. R.−2020	2	——/——	22 (13)	——	——	9	13	5.90	[1.66; 20.93]
Total (common effect)								5.54	[2.61; 11.75]
Total (random effect)								5.54	[2.61; 11.75]
Heterogeneity: $\chi^2_1 = 0.01$ (P = .90), I^2 = 0%									
Total (common effect)								7.30	[4.29; 12.41]
Total (random effect)								7.30	[4.29; 12.41]

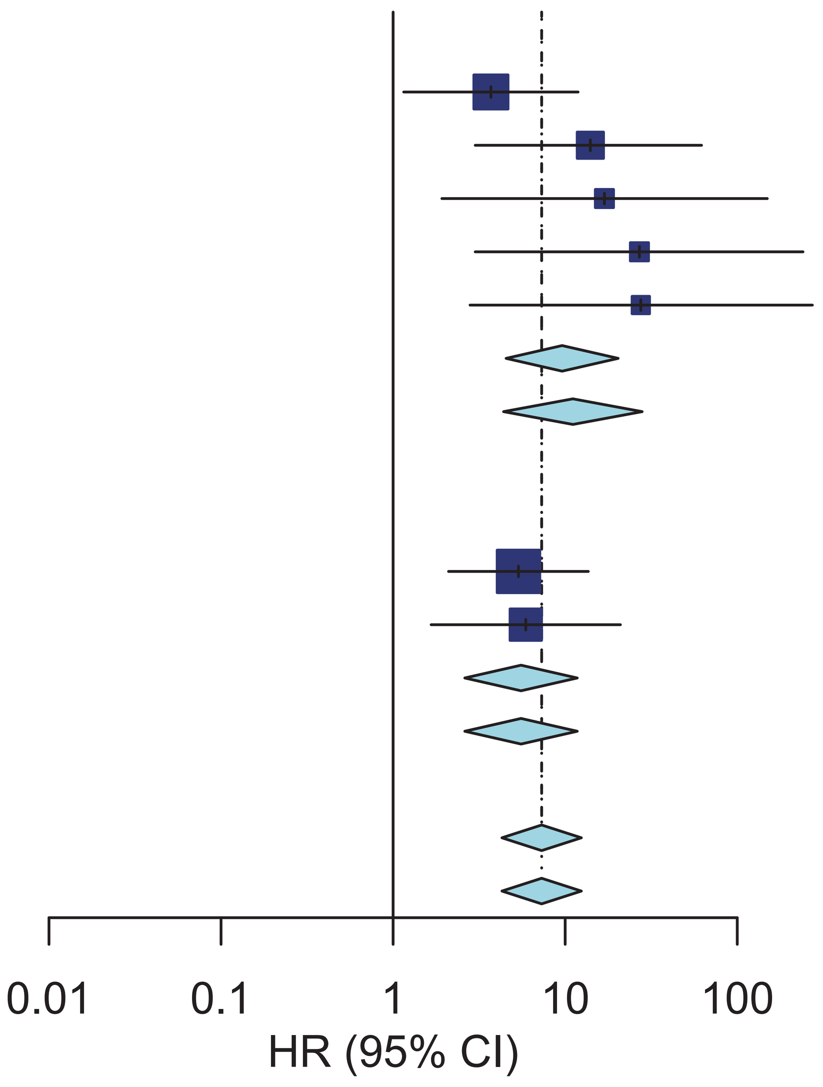

Heterogeneity: $\chi^2_6 = 5.77$ (P = .45), I^2 = 0%
Test for subgroup differences (common effect): $\chi^2_1 = 1.03$ (P = .31)
Test for subgroup differences (random effects): $\chi^2_1 = 1.30$ (P = .25)

Figure S8 Subgroup for Pooled HR of univariate analysis of ESCA recurrence monitoring time; 1=landmark detection; 2=longitudinal detection; Negative=ctDNA-; Positive=ctDNA+; Detection =the time of ctDNA detection after surgery; Adj=adjuvant therapy; d=day; w=week; m=month; y=year; Two arms: Openshaw, M. R-2020; N of event: total sample (sample of recurrence); Solid line is invalid line, and 95% confidence interval crossing is not statistically significant. Vertical dashed lines are pooled HR. I^2 was estimated by Higgins' approach. x^2 was estimated by Q-test.
