# Supplementary material for: Circulating tumour DNA-Based molecular residual disease detection in resectable cancers: a systematic review and meta-analysis
Source: eBioMedicine. 2024 Apr 13;103:105109. doi: 10.1016/j.ebiom.2024.105109 (PMC11021841; doi:10.1016/j.ebiom.2024.105109)
Supplement: Figure S9 [file mmc21.pdf]

Source	Time	Sex (female/male)	N of event	Detection	Adj	Positive	Negative	HR	95% CI
1									
Wang, D. S−2021	1	29/53	82 (47)	38d	——	34	48	2.67	[1.49; 4.80]
Liu, W−2023	1	36/98	134 (84)	31d	——	42	92	2.96	[1.91; 4.60]
Nishioka, Y.−2022	1	42/63	105 (66)	——	——	32	73	3.10	[1.92; 5.01]
Michael J−2017	1	——/——	54 (——)	17d	——	24	30	3.10	[1.70; 9.10]
Newhook, T. E−2022	1	——/——	48 (34)	18d (13 to 68d)	——	18	30	3.23	[1.61; 6.49]
Bolhuis, K.−2021	1	8/15	23 (17)	3m	Yes	6	17	3.30	[1.10; 9.60]
Jiang, H−2023	1	29/38	67 (41)	28d	Yes	15	52	3.60	[1.48; 8.74]
Schøler, L. V−2017	1	6/15	21 (10)	3m	——	6	15	4.90	[1.50; 15.70]
Tie, J−2021	1	14/35	49 (21)	4w to 10w	——	12	37	6.31	[2.59; 15.37]
Reinert, T−2022	1	——/——	40 (28)	30d	——	13	27	7.60	[3.00; 19.70]
Total (common effect)								3.39	[2.73; 4.21]
Total (random effect)								3.39	[2.73; 4.21]
Heterogeneity: $\chi^2_9 = 6.3$ (P = .71), I^2 = 0%									
2									
Reinert, T−2022	2	——/——	67 (47)	3m/follow−up	——	34	33	4.30	[2.20; 8.10]
3									
Wang, D. S−2021	3	19/30	49 (12)	——	——	22	27	2.46	[1.15; 5.28]
Tie, J−2021	3	——/——	45 (——)	——	——	11	34	14.90	[4.94; 44.70]
Total (common effect)								4.41	[2.36; 8.25]
Total (random effect)								5.78	[0.99; 33.72]
Heterogeneity: $\chi^2_1 = 6.95$ (P = .008), I^2 = 86%									
Total (common effect)								3.55	[2.92; 4.32]
Total (random effect)								3.55	[2.92; 4.32]

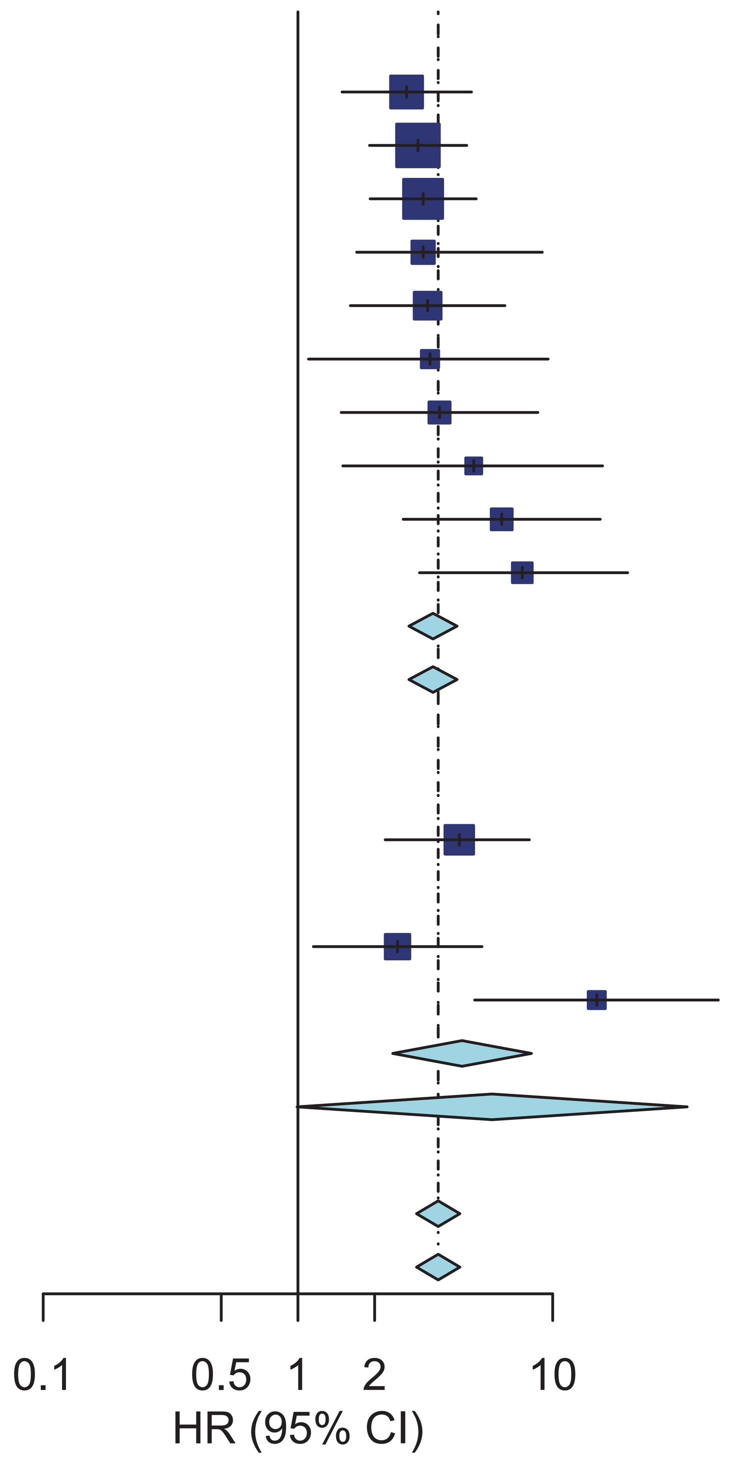

Heterogeneity: $\chi^2_{12} = 14.21$ (P = .29), I^2 = 16%

Test for subgroup differences (common effect): $\chi^2_2 = 0.96$ (P = .62)

Test for subgroup differences (random effects): $\chi^2_2 = 0.78$ (P = .68)

Figure S9 Subgroup for pooled HR of univariate analysis of CRLM recurrence monitoring time; 1=landmark detection, 2=longitudinal detection, 3=post-adjuvant therapy; Negative=ctDNA-; Positive=ctDNA+; Detection=the time of ctDNA detection after surgery; Adj=adjuvant therapy; d=day; w=week; m=month; y=year; Two arms: Wang, D. S-2021; Tie, J-2021; Reinert, T-2022. N of event: total sample (sample of recurrence). Solid line is invalid line, and 95% confidence interval crossing is not statistically significant. Vertical dashed lines are pooled HR. I^2 was estimated by Higgins' approach. x^2 was estimated by Q-test.
