# Supplementary material for: Circulating tumour DNA-Based molecular residual disease detection in resectable cancers: a systematic review and meta-analysis
Source: eBioMedicine. 2024 Apr 13;103:105109. doi: 10.1016/j.ebiom.2024.105109 (PMC11021841; doi:10.1016/j.ebiom.2024.105109)
Supplement: Figure S10 [file mmc22.pdf]

Source	Time	Sex (female/male)	N of event	Detection	Adj	Positive	Negative	HR	95% CI
1									
Yuan, Shu−Qiang−2023	1	34/68	100 (33)	4d (1~7d)	——	25	75	2.74	[1.37; 5.48]
Yang, J.−2020	1	——/——	38 (17)	1m	——	7	31	7.54	[2.68; 21.21]
Xue, Pei−2023	1	——/——	13 (3)	2w to 4w	——	1	12	8.00	[1.39; 46.13]
Leal, A.−2020	1	——/——	50 (6)	6.5w	Yes	9	11	21.80	[3.90; 123.10]
Total (common effect)								4.71	[2.80; 7.93]
Total (random effect)								6.24	[2.59; 15.01]
Heterogeneity: χ^2_3 = 6.52 (P = .09), I^2 = 54%									
2									
Yang, J.−2020	2	——/——	44 (36)	3m/1y−6m/follow−up	——	17	27	17.87	[5.01; 63.76]
3									
Yang, J.−2020	3	——/——	23 (——)	——	——	5	18	9.87	[2.28; 42.74]
Yuan, Shu−Qiang−2023	3	15/26	41 (24)	——	——	10	31	14.99	[3.08; 72.96]
Total (common effect)								11.97	[4.08; 35.08]
Total (random effect)								11.97	[4.08; 35.08]
Heterogeneity: χ^2_1 = 0.14 (P = .70), I^2 = 0%									
Total (common effect)								6.46	[4.16; 10.03]
Total (random effect)								8.44	[4.34; 16.39]

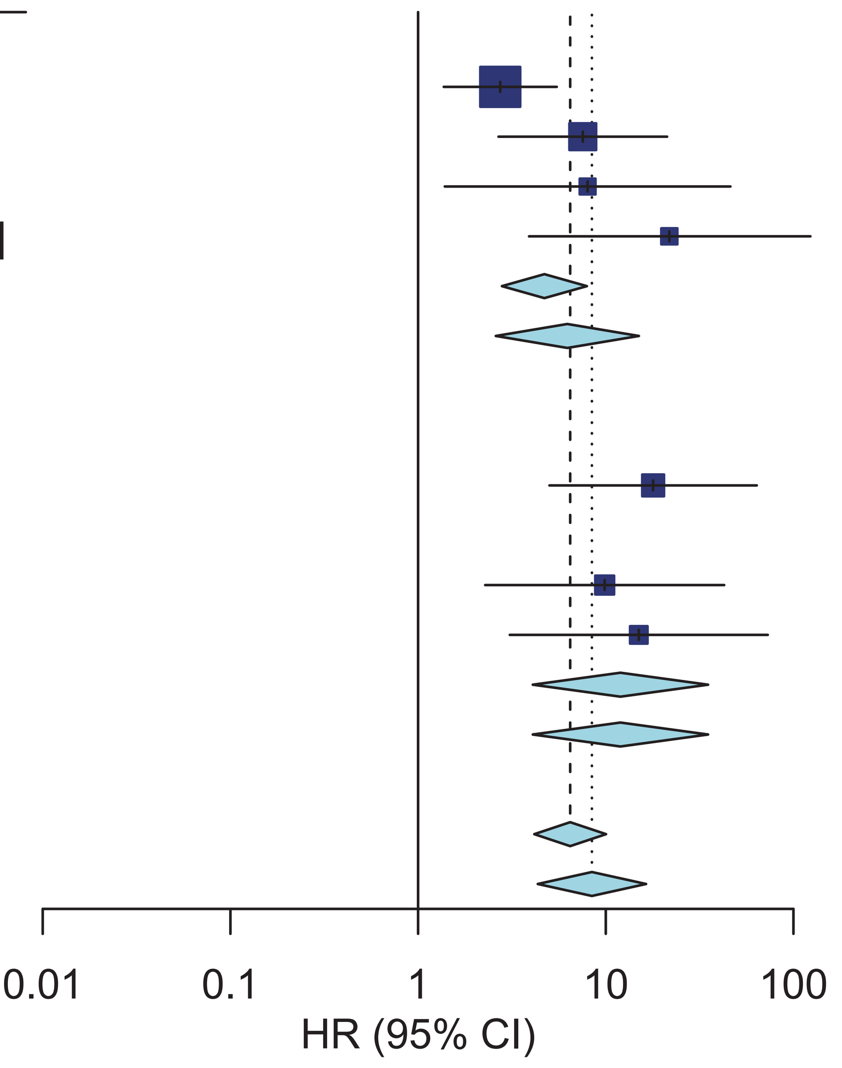

Heterogeneity: χ^2_6 = 11.80 (P = .07), I^2 = 49%
Test for subgroup differences (common effect): χ^2_2 = 5.13 (P = .08)
Test for subgroup differences (random effects): χ^2_2 = 2.00 (P = .37)

Figure S10 Subgroup for pooled HR of univariate analysis of GC recurrence monitoring time; 1=landmark detection, 2=longitudinal detection, 3=post-adjuvant therapy; Negative=ctDNA-; Positive=ctDNA+; Detection=the time of ctDNA detection after surgery; Adj=adjuvant therapy; d=day; w=week; m=month; y=year; Two arms: Yuan, Shu-Qiang-2022; Three arms: Yang, J.-2020; N of event: total sample (sample of recurrence). Solid line is invalid line, and 95% confidence interval crossing is not statistically significant. Vertical dashed lines are pooled HR. I^2 was estimated by Higgins' approach. x^2 was estimated by Q-test.
