# Supplementary material for: Circulating tumour DNA-Based molecular residual disease detection in resectable cancers: a systematic review and meta-analysis
Source: eBioMedicine. 2024 Apr 13;103:105109. doi: 10.1016/j.ebiom.2024.105109 (PMC11021841; doi:10.1016/j.ebiom.2024.105109)
Supplement: Figure S11 [file mmc23.pdf]

Source	Time	Sex (female/male)	N of event	Detection	Adj	Positive	Negative	HR	95% CI
1									
Powles, T−2021	1	62/221	281 (——)	10w	No	98	183	6.30	[4.45; 8.92]
Szabados, B.−2022	1	——/——	36 (6)	——	——	5	31	78.22	[8.64; 707.78]
Total (common effect)								6.70	[4.75; 9.44]
Total (random effect)								17.38	[1.54; 195.81]
Heterogeneity: $\chi_1^2 = 4.9$ (P = .03), I^2 = 80%									
2									
Christensen, Emil−2019	2	——/——	64 (13)	——	——	17	47	131.30	[16.60; 16993.60]
Total (common effect)								6.89	[4.90; 9.70]
Total (random effect)								25.86	[3.29; 202.94]

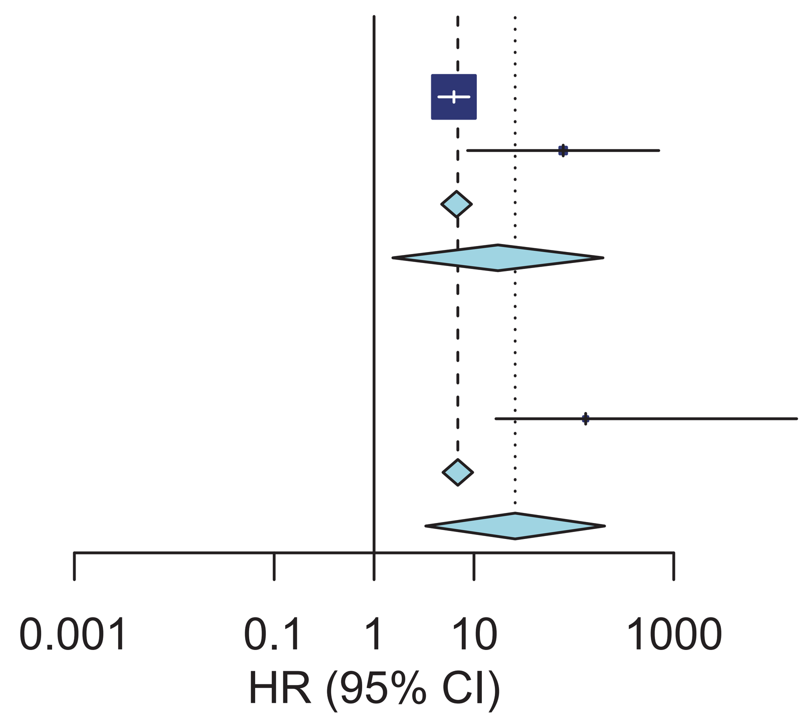

Heterogeneity: $\chi_2^2 = 7.71$ (P = .02), I^2 = 74%
Test for subgroup differences (common effect): $\chi_1^2 = 2.80$ (P = .09)
Test for subgroup differences (random effects): $\chi_1^2 = 0.88$ (P = .35)

Figure S11 Subgroup for pooled HR of univariate analysis of BLCA recurrence monitoring time; 1=landmark detection, 2=longitudinal detection; Negative= ctDNA-; Positive=ctDNA+; Detection=the time of ctDNA detection after surgery; Adj=adjuvant therapy; d=day; w=week; m=month; y=year; N of event: total sample (sample of recurrence). Solid line is invalid line, and 95% confidence interval crossing is not statistically significant. Vertical dashed lines are pooled HR. I^2 was estimated by Higgins' approach. x^2 was estimated by Q-test.
