# Supplementary material for: Circulating tumour DNA-Based molecular residual disease detection in resectable cancers: a systematic review and meta-analysis
Source: eBioMedicine. 2024 Apr 13;103:105109. doi: 10.1016/j.ebiom.2024.105109 (PMC11021841; doi:10.1016/j.ebiom.2024.105109)
Supplement: Figure S12 [file mmc24.pdf]

Cancer	Positive	Negative	HR(95% CI)	Iv2 (H)	P (Q)
Pancreatic adenocarcinoma (PAAD)					
1	54	128	3.46 (2.01 to 5.95)	0.24	0.26
Colorectal liver metastases (CRLM)					
1	202	421	3.39 (2.73 to 4.21)	0	0.71
2*	34	33	4.30 (2.20 to 8.10)	——	——
3	33	61	5.78 (0.99 to 33.72)	0.86	0.008
Ovarian cancer(OV)					
1	81	49	4.47 (2.38 to 8.40)	0	0.44
Gastric cancer (GC)					
1	42	129	6.24 (2.59 to 15.01)	0.54	0.09
2*	17	27	17.87 (5.01 to 63.76)	——	——
3	15	49	11.97 (4.08 to 35.08)	0	0.7
Melanoma					
1	22	104	7.40 (3.34 to 16.38)	0.14	0.31
Non−small cell lung cancer (NSCLC)					
1	217	1403	5.95 (4.30 to 8.23)	0.43	0.05
2	297	824	10.42 (5.58 to 19.45)	0.8	<0.001
3	16	84	4.12 (1.77 to 9.60)	0.04	0.31
Esophageal carcinoma(ESCA)					
1	25	88	11.08 (4.38 to 28.01)	0.15	0.32
2	19	66	5.54 (2.61 to 11.75)	0	0.9
Breast cancer(BC)					
1	41	157	9.90 (4.39 to 22.31)	0.36	0.18
2	29	63	19.23 (6.66 to 55.57)	0.15	0.28
Colorectal cancer (CRC)					
1	1074	5764	7.88 (5.75 to 10.79)	0.86	<0.001
2	141	493	23.49 (12.34 to 44.70)	0.32	0.21
3	65	521	12.47 (5.73 to 27.12)	0.67	0.009
Hepatocellular carcinoma (HCC)*					
2*	21	38	11.77 (4.96 to 27.96)	——	——
Bladder carcinoma(BLCA)					
1	103	214	17.38 (1.54 to 195.81)	0.8	0.03
2*	17	47	131.30 (16.60 to 16993.60)	——	——

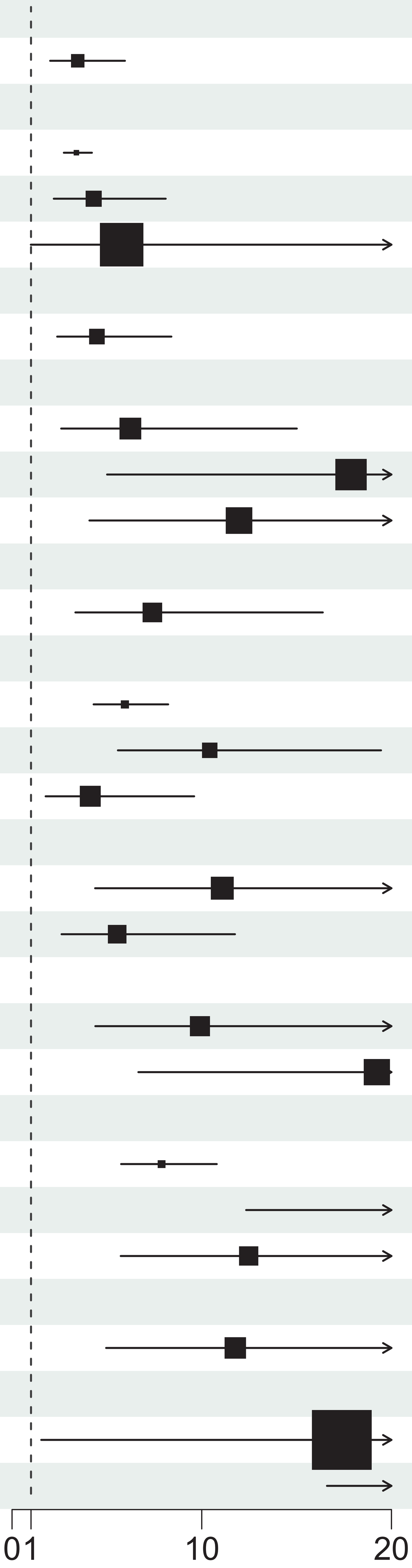

Figure S12 Subgroup for pooled HR of univariate analysis of recurrence detection of CRC, NSCLC, CRLM, PAAD, BLCA, melanoma, BC, GC, HCC, OV and ESCA; 1=landmark detection, 2=longitudinal detection, 3=post-adjuvant therapy; *=single study; Negative=ctDNA-; Positive=ctDNA+; Vertical dashed lines are invalid lines, and 95% confidence interval crossing is not statistically significant; H=Higgins' approach, Q=Q-test.
