# Supplementary material for: Circulating tumour DNA-Based molecular residual disease detection in resectable cancers: a systematic review and meta-analysis
Source: eBioMedicine. 2024 Apr 13;103:105109. doi: 10.1016/j.ebiom.2024.105109 (PMC11021841; doi:10.1016/j.ebiom.2024.105109)
Supplement: Figure S13 [file mmc25.pdf]

Source	Time	Sex (female/male)	N of event	Detection	Adj	Positive	Negative	HR	95% CI
1									
Taieb, J−2021	1	441/576	1017 (298)	42d	——	140	877	1.46	[1.08; 1.97]
Tie, J−2022	1	——/——	291 (23)	4w or 7w−re/de	——	45	246	1.83	[0.79; 4.27]
Tie, J−2022	1	——/——	291 (22)	4w or 7w−re	——	45	246	2.45	[1.00; 5.99]
Li,Y−2022	1	——/——	151 (39)	30d	——	24	127	5.31	[2.56; 10.99]
Loupakis, F−2021	1	40/72	112 (82)	27d	Yes	61	51	5.80	[3.50; 9.70]
Loupakis, F−2021	1	——/——	47 (35)	27d	No	30	17	15.00	[4.30; 49.00]
Tarazona, N−2019	1	——/——	69 (18)	6w to 8w	——	14	55	6.96	[2.57; 18.91]
Henriksen, T. V−2022	1	——/——	140 (38)	2w	——	20	120	7.00	[3.70; 13.50]
Reinert, T−2019	1	——/——	94 (17)	30d	——	10	84	7.20	[2.70; 19.00]
Bryant,C −2021	1	——/——	45 (27)	3w	——	25	20	7.70	[2.60; 22.50]
Han, S. W.−2022	1	——/——	71 (23)	3w	——	13	58	8.23	[3.30; 21.00]
Anandappa, Gayathri−2021	1	——/——	64 (14)	——	——	11	53	9.00	[2.60; 32.00]
Kotani, D−2023	1	489/550	1039 (196)	4w	——	187	852	10.00	[7.70; 14.00]
Hofste, Lisa S. M.−2023	1	——/——	19 (5)	1w to 2w	——	3	16	10.90	[1.10; 106.70]
Chen, G−2021	1	106/134	240 (23)	3d to 7d	——	20	220	10.98	[5.31; 22.72]
Tie, J−2019	1	52/107	159 (23)	4w to 10w	All	19	140	13.00	[5.50; 31.00]
Tie, J−2019	1	——/——	102 (——)	4w to 10w	Yes	11	91	10.00	[3.40; 29.00]
Tie, J−2019	1	——/——	57 (——)	4w to 10w	No	8	49	22.00	[4.20; 110.00]
Tie, J−2016	1	99/131	230 (——)	4w to 10w	All	20	210	13.00	[6.60; 27.00]
Tie, J−2016	1	——/——	44 (——)	4w to 10w	Yes	3	41	11.00	[1.80; 68.00]
Tie, J−2016	1	——/——	178 (27)	4w to 10w	No	14	164	18.00	[7.90; 40.00]
Watanabe, Jun−2023	1	——/——	2083 (——)	1m	——	286	1797	12.00	[9.10; 15.00]
Zhou, Jian−2022	1	——/——	40 (——)	8d	——	6	34	16.57	[3.01; 91.36]
Mo, S.−2023	1	——/——	255 (50)	1m	——	59	196	17.50	[8.90; 34.40]
Li, N−2022	1	——/——	116 (——)	1m	——	12	104	3.04	[1.22; 7.58]
Kuang, P. P.−2020	1	——/——	35 (9)	2w	——	8	27	3.69	[1.11; 12.25]
Wang, S−2022	1	——/——	116 (——)	7d	——	16	100	3.90	[1.85; 8.20]
Qiu, B−2021	1	——/——	85 (33)	30d	——	18	67	3.95	[1.96; 7.96]
Chen, K−2023	1	——/——	148 (——)	3d to 1m	——	23	125	4.13	[1.90; 9.00]
Wang, S−2022	1	——/——	114 (——)	3m	——	16	98	4.32	[2.06; 9.08]
Fu, R−2023	1	——/——	146 (38)	1m (+−7d)	——	36	110	5.07	[2.33; 11.01]
Wang, S−2022	1	——/——	89 (——)	6m	——	11	78	6.19	[2.44; 15.69]
Chen, K−2019	1	——/——	25 (9)	3d	——	7	18	7.55	[2.09; 27.27]
Waldeck, S−2022	1	——/——	16 (8)	1w to 2w	——	4	12	10.64	[1.65; 68.76]
Xia, L−2022	1	——/——	329 (70)	3d to 1m	——	26	303	11.10	[6.50; 19.00]
Zhang, J. T.−2022	1	——/——	245 (47)	1m(±7d)	——	21	224	12.50	[3.08; 50.60]
Chen, K−*2023	1	——/——	156 (——)	1m	——	19	137	16.40	[7.84; 34.31]
Yuan, Shu−Qiang−2022	1	34/68	100 (33)	4d (1~7d)	——	25	75	2.74	[1.37; 5.48]
Yang, J.−2020	1	——/——	38 (17)	1m	——	7	31	7.54	[2.68; 21.21]
Xue, Pei−2023	1	——/——	13 (3)	2w to 4w	——	1	12	8.00	[1.39; 46.13]
Leal, A.−2020	1	——/——	50 (6)	6.5w	Yes	9	11	21.80	[3.90; 123.10]
Hata, Tatsuo−2022	1	21/45	66 (——)	2w	——	16	50	2.11	[1.04; 4.28]
Popova, A.−2020	1	——/——	37 (10)	7d (5−9d)	——	14	23	2.90	[1.01; 8.50]
Jiang, J.−2020	1	——/——	27 (14)	7d	——	9	18	3.55	[0.90; 13.89]
Lee, B−2019	1	——/——	35 (23)	4w to 8w	——	13	22	5.40	[1.90; 15.20]
Wang, Xiuchao−2023	1	——/——	17 (4)	7d	——	2	15	16.87	[2.51; 113.24]
Wang, D. S−2021	1	29/53	82 (47)	38d	——	34	48	2.67	[1.49; 4.80]
Liu, W−2023	1	36/98	134 (84)	31d	——	42	92	2.96	[1.91; 4.60]
Nishioka, Y.−2022	1	42/63	105 (66)	——	——	32	73	3.10	[1.92; 5.01]
Michael J−2017	1	——/——	54 (——)	17d	——	24	30	3.10	[1.70; 9.10]
Newhook, T. E−2022	1	——/——	48 (34)	18d (13 to 68d)	——	18	30	3.23	[1.61; 6.49]
Bolhuis, K.−2021	1	8/15	23 (17)	3m	Yes	6	17	3.30	[1.10; 9.60]
Jiang, H−2023	1	29/38	67 (41)	28d	Yes	15	52	3.60	[1.48; 8.74]
Schøler, L. V−2017	1	6/15	21 (10)	3m	——	6	15	4.90	[1.50; 15.70]
Tie, J−2021	1	14/35	49 (21)	4w to 10w	——	12	37	6.31	[2.59; 15.37]
Reinert, T−2022	1	——/——	40 (28)	30d	——	13	27	7.60	[3.00; 19.70]
Sharma, P−2022	1	——/——	47 (14)	——	——	16	31	3.02	[1.01; 9.01]
Coombes, R. C.−2019	1	——/——	49 (18)	——	——	9	40	11.80	[4.30; 32.50]
Chen, Y. H.−2017	1	——/——	33 (13)	——	——	4	29	12.60	[3.06; 52.20]
Zhou, Y−2021	1	——/——	32 (7)	3d to 7d	Yes	5	27	23.53	[1.90; 290.90]
Garcia−Murillas, Isaac−2015	1	37/0	37 (12)	2w to 4w	——	7	30	25.10	[4.08; 130.50]
Openshaw, M. R.−2020	1	——/——	22 (13)	——	——	6	16	3.70	[1.15; 11.89]
Takei, Shogo−2023	1	——/——	28 (——)	4w	——	3	25	14.00	[3.00; 62.00]
Morimoto, Y.−2023	1	3/13	16 (7)	1m or 3m	——	6	10	16.90	[1.92; 149.40]
Gerlinger, Marco−2023	1	——/——	24 (——)	——	——	6	18	27.00	[3.00; 241.00]
Liu, T.−2021	1	——/——	23 (5)	1w	——	4	19	27.50	[2.80; 273.10]
Ananda, Sumitra−2023	1	——/——	81 (——)	——	——	63	18	3.28	[1.39; 7.72]
Chao, A.−2022	1	——/——	29 (8)	7d to 10d	——	11	18	5.34	[1.87; 15.27]
Chapman, Jocelyn S.−2021	1	——/——	20 (7)	——	——	7	13	12.75	[1.70; 94.00]
Powles, T−2021	1	62/221	281 (——)	10w	No	98	183	6.30	[4.45; 8.92]
Szabados, B.−2022	1	——/——	36 (6)	——	——	5	31	78.22	[8.64; 707.78]
Tan, L.−2019	1	——/——	52 (29)	2w	——	13	39	10.00	[4.30; 24.00]
Genta, Sofia−2022	1	12/33	45 (——)	——	——	4	41	2.71	[0.60; 12.31]
Eroglu, Zeynep−2023	1	9/20	29 (——)	——	——	5	24	10.77	[1.77; 65.57]
Total (common effect)								5.86	[5.37; 6.39]
Total (random effect)								6.24	[5.26; 7.40]
Heterogeneity: $\chi^2_{73} = 270.97$ ($P < .001$), $I^2 = 73\%$									
2									
Tarazona, N−2019	2	33/61	94 (18)	4m/follow−up	——	32	62	8.03	[1.79; 35.98]
Henriksen, T. V−2022	2	——/——	114 (24)	——	——	22	92	50.80	[14.90; 172.00]
Mo, S.−2023	2	——/——	226 (——)	3m/follow−up	——	47	179	13.50	[5.40; 33.80]
Chen, G−2021	2	——/——	137 (——)	6m/once−3m/follow−up	——	25	100	32.02	[10.79; 95.08]
Reinert, T−2019	2	——/——	75 (16)	3m/follow−up	——	15	60	43.50	[9.80; 193.50]
Peng, M−2020	2	20/51	71 (31)	——	——	30	41	3.11	[1.47; 6.55]
Li, N−2022	2	49/70	119 (28)	3m to 6m/follow−up	——	37	82	3.46	[1.59; 7.55]
Fu, R−2023	2	81/96	177 (41)	3m to 6m/follow−up	——	55	122	7.14	[3.60; 14.15]
Wang, S−2022	2	——/——	117 (34)	3m/follow−up	——	40	77	7.59	[3.53; 16.32]
Yang, W.−2020	2	49/33	82 (5)	3m/1y−6m/2~3y	——	15	67	8.50	[1.30; 56.30]
Qiu, B−2021	2	——/——	89 (34)	3m/follow−up	——	35	54	8.55	[3.70; 19.74]
Chen, K−2023	2	——/——	63 (——)	——	——	8	55	15.14	[4.45; 51.45]
Tan, A.−2021	2	23/34	57 (——)	——	——	8	49	22.00	[3.60; 133.80]
Chen, K−*2023	2	56/54	110 (19)	——	——	23	87	37.42	[10.76; 130.16]
Zhang, J. T.−2022	2	——/——	236 (47)	3m to 6m/follow−up	——	46	190	50.00	[25.00; 100.00]
Yang, J.−2020	2	——/——	44 (36)	3m/1y−6m/follow−up	——	17	27	17.87	[5.01; 63.76]
Reinert, T−2022	2	——/——	67 (47)	3m/follow−up	——	34	33	4.30	[2.20; 8.10]
Garcia−Murillas, Isaac−2015	2	43/0	43 (15)	6m/follow−up	——	13	30	12.00	[3.36; 43.07]
Coombes, R. C.−2019	2	——/——	49 (18)	——	——	16	33	35.80	[8.00; 161.30]
Ococks, E.−2021	2	——/——	63 (26)	——	——	10	53	5.35	[2.10; 13.63]
Openshaw, M. R.−2020	2	——/——	22 (13)	——	——	9	13	5.90	[1.66; 20.93]
Christensen, Emil−2019	2	——/——	64 (13)	——	——	17	47	131.30	[16.60; 16993.60]
Zhao, L−2022	2	——/——	59 (27)	——	——	21	38	11.77	[4.96; 27.96]
Total (common effect)								10.36	[8.43; 12.74]
Total (random effect)								12.23	[8.29; 18.05]
Heterogeneity: $\chi^2_{22} = 74.37$ ($P < .001$), $I^2 = 70\%$									
3									
Li,Y−2022	3	——/——	124 (36)	——	Yes	11	113	2.75	[1.06; 7.13]
Tarazona, N−2019	3	——/——	25 (10)	——	Yes	7	18	10.02	[9.20; 307.30]
Chen, G−2021	3	——/——	137 (——)	——	Yes	12	125	12.76	[5.39; 30.19]
Mo, S.−2023	3	——/——	149 (22)	——	Yes	18	131	13.80	[5.90; 32.10]
Reinert, T−2019	3	13/45	58 (14)	——	Yes	7	51	17.50	[5.40; 56.50]
Henriksen, T. V−2022	3	——/——	93 (20)	——	Yes	10	83	50.76	[15.40; 167.00]
Qiu, B−2021	3	——/——	64 (——)	——	Yes	8	56	3.22	[1.26; 8.18]
Kuang, P. P.−2020	3	——/——	36 (9)	——	Yes	8	28	8.76	[1.63; 47.01]
Yang, J.−2020	3	——/——	23 (——)	——	Yes	5	18	9.87	[2.28; 42.74]
Yuan, Shu−Qiang−2022	3	15/26	41 (24)	——	Yes	10	31	14.99	[3.08; 72.96]
Wang, D. S−2021	3	19/30	49 (12)	——	Yes	22	27	2.46	[1.15; 5.28]
Tie, J−2021	3	——/——	45 (——)	——	Yes	11	34	14.90	[4.94; 44.70]
Total (common effect)								7.98	[5.86; 10.87]
Total (random effect)								9.11	[5.27; 15.75]
Heterogeneity: $\chi^2_{11} = 33.3$ ($P < .001$), $I^2 = 67\%$									
Total (common effect)								6.48	[5.99; 7.00]
Total (random effect)								7.48	[6.39; 8.77]

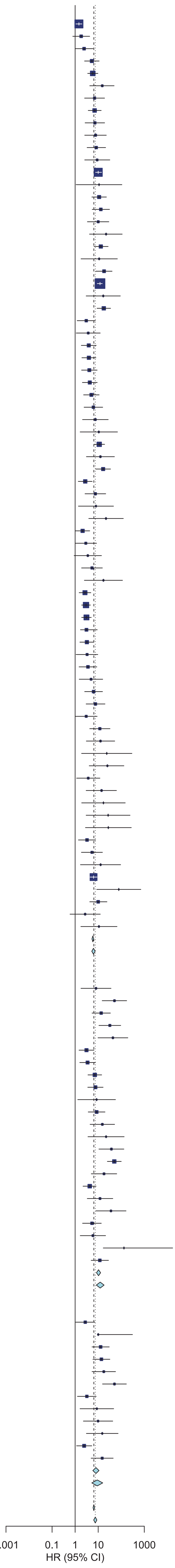

Heterogeneity: $\chi^2_{108} = 405.43$ ($P < .001$), $I^2 = 73\%$
Test for subgroup differences (common effect): $\chi^2_2 = 26.79$ ($P < .001$)
Test for subgroup differences (random effects): $\chi^2_2 = 10.51$ ($P = .005$)

Figure S13 Subgroup analysis for landmark detection, longitudinal detection and post-adjuvant therapy for pooled HR of univariate analysis of pan-cancer recurrence monitoring; Negative=ctDNA-; Positive=ctDNA+; 1=landmark detection, 2=longitudinal detection, 3=post-adjuvant therapy; re/de=the outcome of recurrence or death; re=the outcome of recurrence; Detection=the time of ctDNA detection after surgery; Adj=adjuvant therapy; d=day; w=week; m=month; y=year; Two arms: Tie, J-2022; Li,Y-2022; Loupakis, F-2021; Chen, K-2023; Chen, K-*2023; Fu, R-2023; Kuang, P. P.-2020; Li, N-2022; Zhang, J. T.-2022; Yuan, Shu Qiang-2023; Wang, D. S-2021; Tie, J-2021; Reinert, T-2022; Coombes, R. C-2019; Garcia Murillas, Isaac-2015; Openshaw, M. R-2020; Three arms: Henriksen, T. V-2022, Tie, J-2019, Tie, J-2016, Mo, S.-2023, Chen, G-2021; Qiu, B-2021; Yang, J.-2020; Tarazona, N-2019; Reinert, T-2019; Four arms: Wang, S-2022. N of event: total sample (sample of recurrence). Solid line is invalid line, and 95% confidence interval crossing is not statistically significant. Vertical dashed lines are pooled HR. I^2 was estimated by Higgins' approach. x^2 was estimated by Q-test.
