# Supplementary material for: Circulating tumour DNA-Based molecular residual disease detection in resectable cancers: a systematic review and meta-analysis
Source: eBioMedicine. 2024 Apr 13;103:105109. doi: 10.1016/j.ebiom.2024.105109 (PMC11021841; doi:10.1016/j.ebiom.2024.105109)
Supplement: Figure S14 [file mmc26.pdf]

Source	Time	Sex (female/male)	N of event	Detection	Adj	Positive	Negative	HR	95% CI
**Yes**									
Loupakis, F−2021	1	40/72	159 (23)	27d	Yes	61	51	5.80	[3.50; 9.70]
Tie, J−2019	1	——/——	66 (——)	4w to 10w	Yes	11	91	10.00	[3.40; 29.00]
Tie, J−2016	1	——/——	105 (66)	4w to 10w	Yes	3	41	11.00	[1.80; 68.00]
Total (common effect)								6.63	[4.24; 10.35]
Total (random effect)								6.63	[4.24; 10.35]
Heterogeneity: $\chi_2^2 = 1.13$ (P = .57), I^2 = 0%									
**No**									
Loupakis, F−2021	1	——/——	230 (——)	27d	No	30	17	15.00	[4.30; 49.00]
Tie, J−2019	1	——/——	82 (47)	4w to 10w	No	8	49	22.00	[4.20; 110.00]
Tie, J−2016	1	——/——	23 (17)	4w to 10w	No	14	164	18.00	[7.90; 40.00]
Total (common effect)								17.67	[9.47; 32.96]
Total (random effect)								17.67	[9.47; 32.96]
Heterogeneity: $\chi_2^2 = 0.14$ (P = .93), I^2 = 0%									
Total (common effect)								9.23	[6.42; 13.27]
Total (random effect)								10.90	[6.38; 18.64]

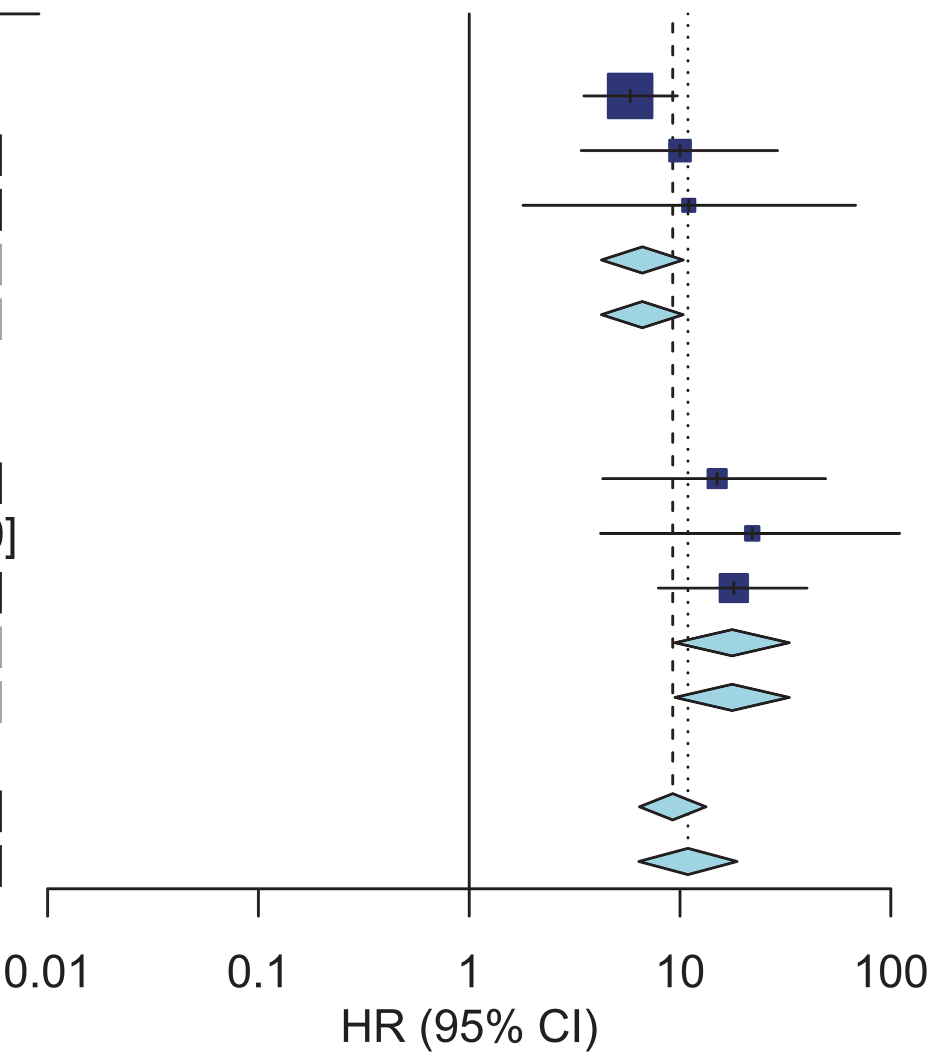

Heterogeneity: $\chi_5^2 = 7.55$ (P = .18), I^2 = 34%
Test for subgroup differences (common effect): $\chi_1^2 = 6.29$ (P = .01)
Test for subgroup differences (random effects): $\chi_1^2 = 6.29$ (P = .01)

Figure S14 Subgroup analysis for whether adjuvant therapy after operation in landmark detection for pooled HR of CRC recurrence monitoring; Negative=ctDNA-; Positive=ctDNA+. N of event: total sample (sample of recurrence). Solid line is invalid line, and 95% confidence interval crossing is not statistically significant. Vertical dashed lines are pooled HR. I^2 was estimated by Higgins' approach. x^2 was estimated by Q-test.
