# Supplementary material for: Circulating tumour DNA-Based molecular residual disease detection in resectable cancers: a systematic review and meta-analysis
Source: eBioMedicine. 2024 Apr 13;103:105109. doi: 10.1016/j.ebiom.2024.105109 (PMC11021841; doi:10.1016/j.ebiom.2024.105109)
Supplement: Figure S15 [file mmc27.pdf]

a

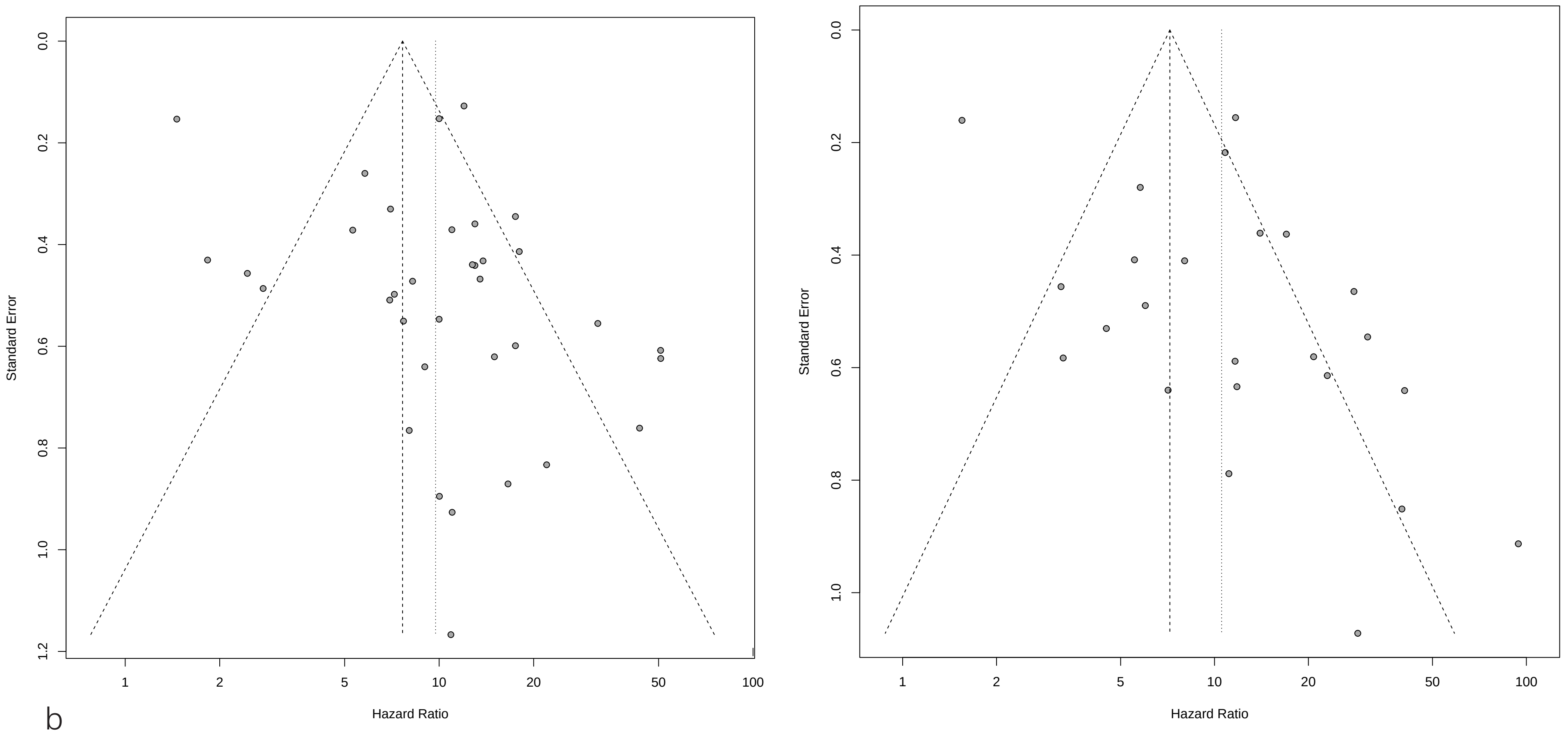

b

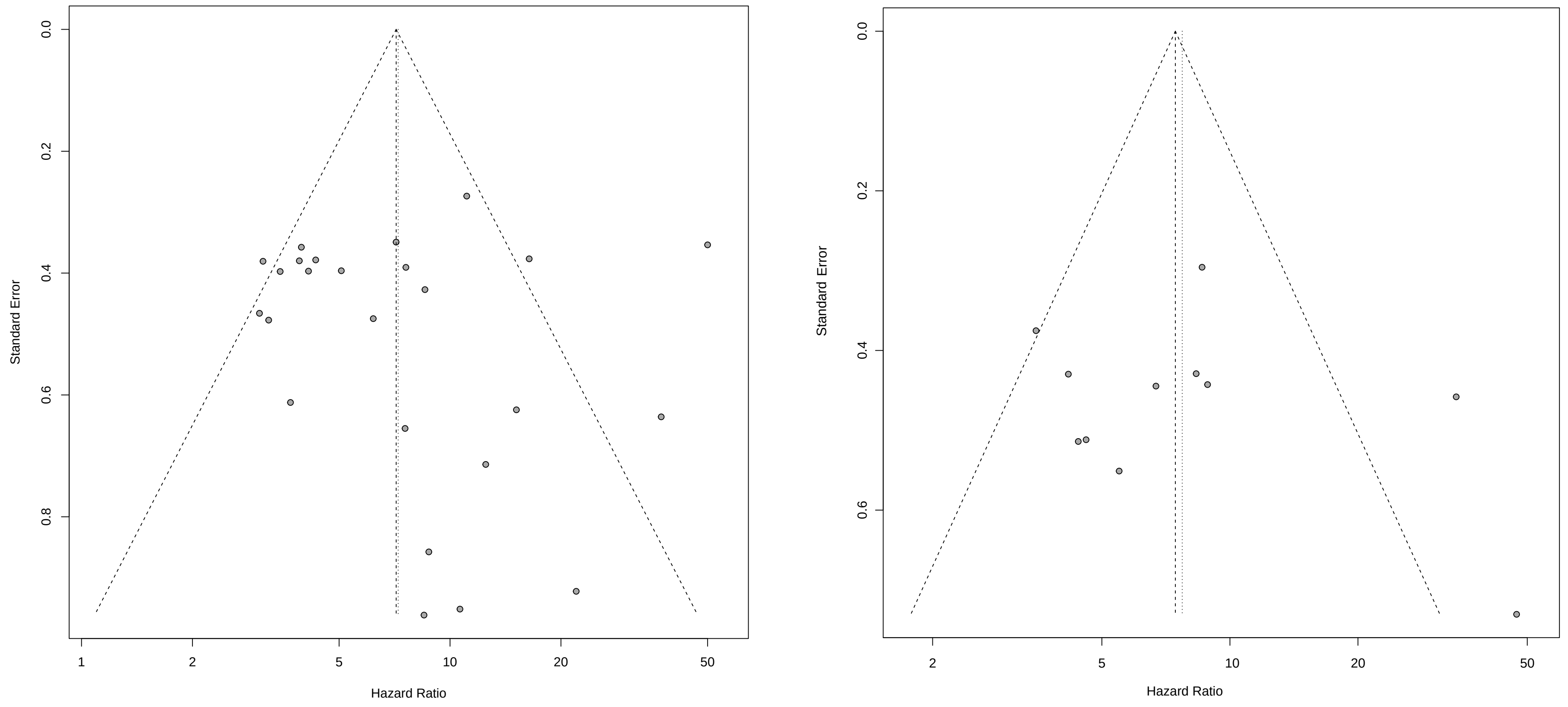

c

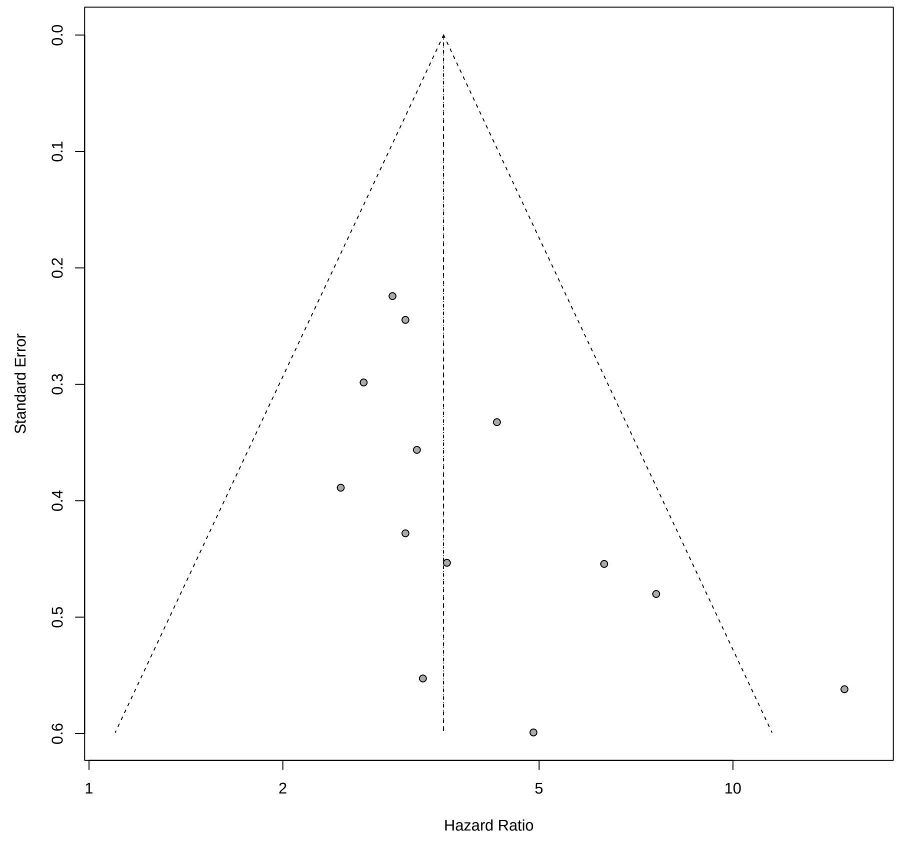

Figure S15 Funnel plot of HR of recurrence of CRC (a):left-Univariate analysis, right-Multivariable analysis, NSCLC (b): left-Univariate analysis, right-Multivariable analysis and CRLM (c): left-Univariate analysis.
