# Supplementary material for: Circulating tumour DNA-Based molecular residual disease detection in resectable cancers: a systematic review and meta-analysis
Source: eBioMedicine. 2024 Apr 13;103:105109. doi: 10.1016/j.ebiom.2024.105109 (PMC11021841; doi:10.1016/j.ebiom.2024.105109)
Supplement: Figure S17 [file mmc29.pdf]

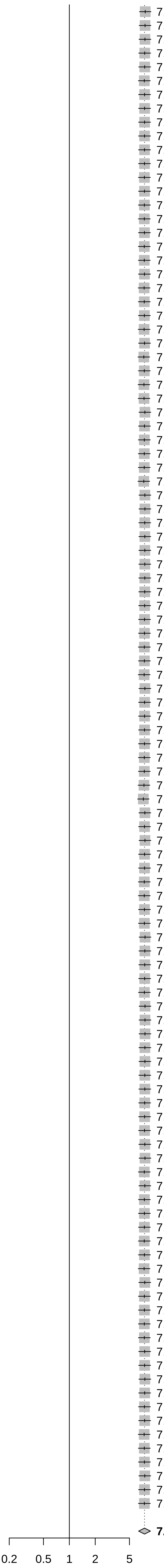

Study	HR	95%−CI	P−value	Tau2	Tau	I2
Omitting Taieb, J−2021	7.62	[6.54; 8.89]	< 0.01	0.3660	0.6050	65%
Omitting Tie, J−2022	7.59	[6.48; 8.88]	< 0.01	0.3993	0.6319	73%
Omitting Tie, J−2022	7.57	[6.46; 8.87]	< 0.01	0.4077	0.6385	73%
Omitting Li,Y−2022	7.52	[6.41; 8.83]	< 0.01	0.4185	0.6469	74%
Omitting Loupakis, F−2021	7.52	[6.40; 8.83]	< 0.01	0.4199	0.6480	74%
Omitting Loupakis, F−2021	7.44	[6.35; 8.73]	< 0.01	0.4131	0.6427	73%
Omitting Tarazona, N−2019	7.50	[6.39; 8.80]	< 0.01	0.4182	0.6467	74%
Omitting Henriksen, T. V−2022	7.50	[6.39; 8.80]	< 0.01	0.4199	0.6480	74%
Omitting Reinert, T−2019	7.49	[6.39; 8.79]	< 0.01	0.4183	0.6467	74%
Omitting Bryant,C −2021	7.49	[6.38; 8.79]	< 0.01	0.4177	0.6463	74%
Omitting Han, S. W.−2022	7.48	[6.38; 8.78]	< 0.01	0.4182	0.6467	74%
Omitting Anandappa, Gayathri−2021	7.48	[6.38; 8.77]	< 0.01	0.4166	0.6454	74%
Omitting Kotani, D−2023	7.46	[6.35; 8.76]	< 0.01	0.4187	0.6471	73%
Omitting Hofste, Lisa S. M.−2023	7.48	[6.38; 8.76]	< 0.01	0.4140	0.6434	74%
Omitting Chen, G−2021	7.45	[6.35; 8.75]	< 0.01	0.4166	0.6455	73%
Omitting Tie, J−2019	7.44	[6.34; 8.73]	< 0.01	0.4143	0.6437	73%
Omitting Tie, J−2019	7.47	[6.37; 8.76]	< 0.01	0.4166	0.6455	74%
Omitting Tie, J−2019	7.44	[6.34; 8.71]	< 0.01	0.4109	0.6410	73%
Omitting Tie, J−2016	7.44	[6.34; 8.72]	< 0.01	0.4141	0.6435	73%
Omitting Tie, J−2016	7.47	[6.37; 8.76]	< 0.01	0.4146	0.6439	74%
Omitting Tie, J−2016	7.41	[6.32; 8.68]	< 0.01	0.4081	0.6388	73%
Omitting Watanabe, Jun−2023	7.43	[6.33; 8.72]	< 0.01	0.4152	0.6444	72%
Omitting Zhou, Jian−2022	7.45	[6.36; 8.74]	< 0.01	0.4130	0.6426	74%
Omitting Mo, S.−2023	7.40	[6.31; 8.67]	< 0.01	0.4074	0.6383	73%
Omitting Tarazona, N−2019	7.49	[6.38; 8.78]	< 0.01	0.4161	0.6450	74%
Omitting Henriksen, T. V−2022	7.35	[6.28; 8.59]	< 0.01	0.3919	0.6260	73%
Omitting Mo, S.−2023	7.44	[6.34; 8.73]	< 0.01	0.4139	0.6433	73%
Omitting Chen, G−2021	7.37	[6.30; 8.63]	< 0.01	0.3991	0.6318	73%
Omitting Reinert, T−2019	7.39	[6.31; 8.65]	< 0.01	0.4018	0.6338	73%
Omitting Li,Y−2022	7.56	[6.45; 8.86]	< 0.01	0.4106	0.6408	73%
Omitting Tarazona, N−2019	7.48	[6.38; 8.77]	< 0.01	0.4150	0.6442	74%
Omitting Chen, G−2021	7.44	[6.35; 8.73]	< 0.01	0.4146	0.6439	73%
Omitting Mo, S.−2023	7.44	[6.34; 8.72]	< 0.01	0.4134	0.6430	73%
Omitting Reinert, T−2019	7.43	[6.34; 8.71]	< 0.01	0.4112	0.6413	73%
Omitting Henriksen, T. V−2022	7.34	[6.28; 8.58]	< 0.01	0.3908	0.6252	73%
Omitting Li, N−2022	7.56	[6.44; 8.86]	< 0.01	0.4119	0.6418	73%
Omitting Peng, M−2020	7.56	[6.45; 8.87]	< 0.01	0.4111	0.6412	73%
Omitting Kuang, P. P.−2020	7.53	[6.42; 8.83]	< 0.01	0.4150	0.6442	74%
Omitting Wang, S−2022	7.55	[6.43; 8.85]	< 0.01	0.4151	0.6443	73%
Omitting Qiu, B−2021	7.55	[6.43; 8.85]	< 0.01	0.4153	0.6444	73%
Omitting Chen, K−2023	7.54	[6.43; 8.85]	< 0.01	0.4160	0.6450	74%
Omitting Wang, S−2022	7.54	[6.42; 8.84]	< 0.01	0.4165	0.6454	74%
Omitting Fu, R−2023	7.52	[6.41; 8.83]	< 0.01	0.4181	0.6466	74%
Omitting Wang, S−2022	7.51	[6.40; 8.81]	< 0.01	0.4184	0.6468	74%
Omitting Chen, K−2019	7.49	[6.38; 8.78]	< 0.01	0.4169	0.6457	74%
Omitting Waldeck, S−2022	7.47	[6.37; 8.76]	< 0.01	0.4146	0.6439	74%
Omitting Xia, L−2022	7.45	[6.35; 8.74]	< 0.01	0.4167	0.6456	73%
Omitting Zhang, J. T.−2022	7.46	[6.36; 8.75]	< 0.01	0.4146	0.6439	74%
Omitting Chen, K−*2023	7.41	[6.32; 8.69]	< 0.01	0.4096	0.6400	73%
Omitting Li, N−2022	7.55	[6.44; 8.86]	< 0.01	0.4134	0.6429	73%
Omitting Fu, R−2023	7.50	[6.39; 8.80]	< 0.01	0.4197	0.6478	74%
Omitting Wang, S−2022	7.49	[6.38; 8.79]	< 0.01	0.4192	0.6474	74%
Omitting Yang, W.−2020	7.48	[6.38; 8.77]	< 0.01	0.4150	0.6442	74%
Omitting Qiu, B−2021	7.48	[6.37; 8.78]	< 0.01	0.4184	0.6468	74%
Omitting Chen, K−2023	7.44	[6.35; 8.73]	< 0.01	0.4130	0.6427	73%
Omitting Tan, A.−2021	7.44	[6.35; 8.72]	< 0.01	0.4115	0.6415	73%
Omitting Chen, K−*2023	7.37	[6.30; 8.63]	< 0.01	0.3994	0.6320	73%
Omitting Zhang, J. T.−2022	7.25	[6.22; 8.45]	< 0.01	0.3637	0.6031	71%
Omitting Qiu, B−2021	7.55	[6.44; 8.85]	< 0.01	0.4130	0.6426	73%
Omitting Kuang, P. P.−2020	7.48	[6.38; 8.77]	< 0.01	0.4154	0.6445	74%
Omitting Yuan, Shu−Qiang−2022	7.57	[6.46; 8.88]	< 0.01	0.4077	0.6385	73%
Omitting Yang, J.−2020	7.49	[6.38; 8.79]	< 0.01	0.4179	0.6465	74%
Omitting Xue, Pei−2023	7.49	[6.38; 8.78]	< 0.01	0.4154	0.6445	74%
Omitting Leal, A.−2020	7.44	[6.35; 8.72]	< 0.01	0.4113	0.6413	73%
Omitting Yang, J.−2020	7.43	[6.34; 8.71]	< 0.01	0.4114	0.6414	73%
Omitting Yang, J.−2020	7.47	[6.37; 8.77]	< 0.01	0.4156	0.6447	74%
Omitting Yuan, Shu−Qiang−2023	7.45	[6.36; 8.74]	< 0.01	0.4135	0.6430	74%
Omitting Hata, Tatsuo−2022	7.59	[6.48; 8.88]	< 0.01	0.4003	0.6327	73%
Omitting Popova, A.−2020	7.55	[6.44; 8.85]	< 0.01	0.4122	0.6420	73%
Omitting Jiang, J.−2020	7.53	[6.42; 8.83]	< 0.01	0.4148	0.6441	74%
Omitting Lee, B−2019	7.51	[6.40; 8.82]	< 0.01	0.4176	0.6462	74%
Omitting Wang, Xiuchao−2023	7.46	[6.36; 8.74]	< 0.01	0.4130	0.6427	74%
Omitting Wang, D. S−2021	7.58	[6.47; 8.88]	< 0.01	0.4054	0.6367	73%
Omitting Liu, W−2023	7.58	[6.47; 8.88]	< 0.01	0.4068	0.6378	73%
Omitting Nishioka, Y.−2022	7.57	[6.46; 8.88]	< 0.01	0.4086	0.6393	73%
Omitting Michael J−2017	7.56	[6.45; 8.86]	< 0.01	0.4118	0.6417	73%
Omitting Newhook, T. E−2022	7.56	[6.45; 8.87]	< 0.01	0.4116	0.6415	73%
Omitting Bolhuis, K.−2021	7.54	[6.43; 8.85]	< 0.01	0.4138	0.6433	74%
Omitting Jiang, H−2023	7.55	[6.43; 8.85]	< 0.01	0.4143	0.6437	73%
Omitting Schøler, L. V−2017	7.52	[6.41; 8.82]	< 0.01	0.4168	0.6456	74%
Omitting Tie, J−2021	7.51	[6.40; 8.81]	< 0.01	0.4186	0.6470	74%
Omitting Reinert, T−2022	7.49	[6.38; 8.79]	< 0.01	0.4183	0.6468	74%
Omitting Reinert, T−2022	7.54	[6.43; 8.85]	< 0.01	0.4165	0.6453	74%
Omitting Wang, D. S−2021	7.58	[6.47; 8.88]	< 0.01	0.4059	0.6371	73%
Omitting Tie, J−2021	7.44	[6.34; 8.72]	< 0.01	0.4130	0.6426	73%
Omitting Sharma, P−2022	7.55	[6.44; 8.85]	< 0.01	0.4128	0.6425	73%
Omitting Coombes, R. C.−2019	7.45	[6.36; 8.74]	< 0.01	0.4155	0.6446	74%
Omitting Chen, Y. H.−2017	7.46	[6.36; 8.75]	< 0.01	0.4146	0.6439	74%
Omitting Zhou, Y−2021	7.46	[6.36; 8.74]	< 0.01	0.4123	0.6421	74%
Omitting Garcia−Murillas, Isaac−2015	7.43	[6.34; 8.71]	< 0.01	0.4103	0.6405	73%
Omitting Garcia−Murillas, Isaac−2015	7.46	[6.36; 8.75]	< 0.01	0.4150	0.6442	74%
Omitting Coombes, R. C.−2019	7.40	[6.32; 8.67]	< 0.01	0.4047	0.6361	73%
Omitting Openshaw, M. R.−2020	7.53	[6.42; 8.83]	< 0.01	0.4151	0.6442	74%
Omitting Takei, Shogo−2023	7.46	[6.36; 8.74]	< 0.01	0.4139	0.6433	74%
Omitting Morimoto, Y.−2023	7.46	[6.37; 8.75]	< 0.01	0.4131	0.6427	74%
Omitting Gerlinger, Marco−2023	7.45	[6.35; 8.73]	< 0.01	0.4113	0.6414	74%
Omitting Liu, T.−2021	7.45	[6.36; 8.73]	< 0.01	0.4115	0.6415	74%
Omitting Ococks, E.−2021	7.52	[6.41; 8.82]	< 0.01	0.4179	0.6464	74%
Omitting Openshaw, M. R.−2020	7.50	[6.40; 8.80]	< 0.01	0.4170	0.6457	74%
Omitting Ananda, Sumitra−2023	7.55	[6.44; 8.86]	< 0.01	0.4128	0.6425	73%
Omitting Chao, A.−2022	7.51	[6.40; 8.82]	< 0.01	0.4175	0.6461	74%
Omitting Chapman, Jocelyn S.−2021	7.47	[6.37; 8.75]	< 0.01	0.4140	0.6434	74%
Omitting Powles, T−2021	7.51	[6.40; 8.82]	< 0.01	0.4209	0.6488	74%
Omitting Szabados, B.−2022	7.41	[6.33; 8.68]	< 0.01	0.4053	0.6366	73%
Omitting Christensen, Emil−2019	7.44	[6.35; 8.72]	< 0.01	0.4098	0.6402	73%
Omitting Tan, L.−2019	7.47	[6.36; 8.76]	< 0.01	0.4173	0.6460	74%
Omitting Genta, Sofia−2022	7.54	[6.43; 8.83]	< 0.01	0.4132	0.6428	74%
Omitting Eroglu, Zeynep−2023	7.47	[6.37; 8.76]	< 0.01	0.4147	0.6440	74%
Omitting Zhao, L−2022	7.45	[6.35; 8.74]	< 0.01	0.4156	0.6447	73%
**Random effects model**	**7.48**	**[6.39; 8.77]**	**< 0.01**	**0.4122**	**0.6420**	**73%**

Figure S17 The result of sensitivity analysis of HR (Univariate analysis) of recurrence of pan-cancer.
