# Supplementary material for: Circulating tumour DNA-Based molecular residual disease detection in resectable cancers: a systematic review and meta-analysis
Source: eBioMedicine. 2024 Apr 13;103:105109. doi: 10.1016/j.ebiom.2024.105109 (PMC11021841; doi:10.1016/j.ebiom.2024.105109)
Supplement: Figure S19 [file mmc31.pdf]

Study	Hazard Ratio	HR	95%−CI	P−value	Tau2	Tau	I2
Omitting Taieb, J−2021		7.28	[5.86; 9.05]	< 0.01	0.4802	0.6929	73%
Omitting Benhaim, L.−2021		7.18	[5.73; 8.99]	< 0.01	0.5364	0.7324	79%
Omitting Reinert, T−2019		7.13	[5.69; 8.94]	< 0.01	0.5420	0.7362	79%
Omitting Li,Y−2022		7.12	[5.68; 8.92]	< 0.01	0.5453	0.7384	79%
Omitting Loupakis, F−2021		7.12	[5.67; 8.93]	< 0.01	0.5473	0.7398	79%
Omitting Tie, J−2019		7.10	[5.67; 8.90]	< 0.01	0.5444	0.7378	79%
Omitting McNamara, S−2022		7.08	[5.65; 8.87]	< 0.01	0.5420	0.7362	79%
Omitting Chen, G−2021		7.07	[5.64; 8.86]	< 0.01	0.5448	0.7381	79%
Omitting Kotani, D−2023		7.01	[5.59; 8.79]	< 0.01	0.5411	0.7356	79%
Omitting Tarazona, N−2019		7.03	[5.61; 8.80]	< 0.01	0.5381	0.7336	79%
Omitting Watanabe, Jun−2023		6.99	[5.58; 8.77]	< 0.01	0.5386	0.7339	78%
Omitting Tie, J−2016		6.98	[5.57; 8.74]	< 0.01	0.5330	0.7301	79%
Omitting Mo, S.−2023		6.94	[5.55; 8.68]	< 0.01	0.5254	0.7248	79%
Omitting Tie, J−2016		6.88	[5.52; 8.57]	< 0.01	0.5062	0.7115	79%
Omitting Anandappa, Gayathri−2021		6.99	[5.60; 8.74]	< 0.01	0.5286	0.7271	79%
Omitting Henriksen, T. V−2022		6.89	[5.52; 8.58]	< 0.01	0.5076	0.7125	79%
Omitting Tarazona, N−2019		7.04	[5.63; 8.81]	< 0.01	0.5376	0.7332	79%
Omitting Chen, G−2021		6.94	[5.56; 8.67]	< 0.01	0.5225	0.7228	79%
Omitting Reinert, T−2019		6.93	[5.56; 8.65]	< 0.01	0.5177	0.7195	79%
Omitting Henriksen, T. V−2022		6.88	[5.52; 8.57]	< 0.01	0.5038	0.7098	79%
Omitting Li,Y−2022		7.16	[5.72; 8.96]	< 0.01	0.5375	0.7331	79%
Omitting Reinert, T−2019		7.03	[5.61; 8.80]	< 0.01	0.5377	0.7333	79%
Omitting Chen, G−2021		6.95	[5.56; 8.69]	< 0.01	0.5245	0.7242	79%
Omitting Henriksen, T. V−2022		6.87	[5.52; 8.55]	< 0.01	0.5005	0.7074	79%
Omitting Qiu, B−2021		7.18	[5.73; 8.99]	< 0.01	0.5380	0.7335	79%
Omitting Wang, S−2022		7.15	[5.70; 8.96]	< 0.01	0.5418	0.7360	79%
Omitting Wang, S−2022		7.13	[5.69; 8.94]	< 0.01	0.5424	0.7365	79%
Omitting Wang, S−2022		7.11	[5.67; 8.91]	< 0.01	0.5432	0.7370	79%
Omitting Xia, L−2022		7.06	[5.62; 8.85]	< 0.01	0.5455	0.7386	79%
Omitting Chen, K−*2023		7.05	[5.63; 8.84]	< 0.01	0.5433	0.7371	79%
Omitting Qiu, B−2021		7.09	[5.66; 8.89]	< 0.01	0.5451	0.7383	79%
Omitting Wang, S−2022		7.06	[5.63; 8.85]	< 0.01	0.5441	0.7377	79%
Omitting Zhang, J. T.−2022		6.84	[5.50; 8.51]	< 0.01	0.4946	0.7033	78%
Omitting Chen, K−*2023		6.89	[5.53; 8.58]	< 0.01	0.5058	0.7112	79%
Omitting Qiu, B−2021		7.14	[5.70; 8.94]	< 0.01	0.5419	0.7362	79%
Omitting Yuan, Shu−Qiang−2022		7.22	[5.77; 9.02]	< 0.01	0.5255	0.7249	79%
Omitting Yang, J.−2020		7.10	[5.66; 8.89]	< 0.01	0.5422	0.7363	79%
Omitting Fedyanin, M.−2020		7.09	[5.66; 8.87]	< 0.01	0.5405	0.7352	79%
Omitting Yang, J.−2020		7.01	[5.60; 8.77]	< 0.01	0.5343	0.7309	79%
Omitting Yang, J.−2020		7.10	[5.68; 8.89]	< 0.01	0.5384	0.7337	79%
Omitting Yuan, Shu−Qiang−2022		7.05	[5.63; 8.82]	< 0.01	0.5380	0.7335	79%
Omitting Yamaguchi, T−2021		7.26	[5.83; 9.05]	< 0.01	0.4957	0.7041	78%
Omitting Hata, Tatsuo−2022		7.20	[5.76; 9.01]	< 0.01	0.5294	0.7276	79%
Omitting Jiang, J.−2020		7.15	[5.71; 8.95]	< 0.01	0.5390	0.7341	79%
Omitting Lee, B−2019		7.10	[5.66; 8.90]	< 0.01	0.5445	0.7379	79%
Omitting Liu, W−2023		7.25	[5.82; 9.05]	< 0.01	0.5048	0.7105	78%
Omitting Nishioka, Y.−2022		7.24	[5.80; 9.04]	< 0.01	0.5115	0.7152	78%
Omitting Tie, J−2021		7.16	[5.72; 8.97]	< 0.01	0.5367	0.7326	79%
Omitting Bolhuis, K.−2021		7.13	[5.70; 8.93]	< 0.01	0.5403	0.7350	79%
Omitting Reinert, T−2022		7.03	[5.62; 8.81]	< 0.01	0.5394	0.7344	79%
Omitting Reinert, T−2022		7.14	[5.69; 8.95]	< 0.01	0.5448	0.7381	79%
Omitting Wang, D. S−2022		7.21	[5.77; 9.02]	< 0.01	0.5253	0.7248	79%
Omitting Schneider, B. P.−2022		7.25	[5.81; 9.04]	< 0.01	0.5099	0.7141	79%
Omitting Chen, Y. H.−2017		7.06	[5.64; 8.84]	< 0.01	0.5388	0.7340	79%
Omitting Garcia−Murillas, Isaac−2015		7.01	[5.61; 8.77]	< 0.01	0.5318	0.7292	79%
Omitting Garcia−Murillas, Isaac−2015		7.05	[5.63; 8.83]	< 0.01	0.5395	0.7345	79%
Omitting Liu, T.−2021		7.00	[5.61; 8.74]	< 0.01	0.5272	0.7261	79%
Omitting Ococks, E.−2021		7.13	[5.69; 8.94]	< 0.01	0.5433	0.7371	79%
Omitting Chao, A.−2022		7.06	[5.64; 8.85]	< 0.01	0.5415	0.7358	79%
Omitting Carrasco, R−2022		7.13	[5.69; 8.93]	< 0.01	0.5401	0.7349	79%
Omitting Powles, T−2021		7.11	[5.66; 8.92]	< 0.01	0.5488	0.7408	79%
Omitting Christensen, Emil−2019		7.00	[5.61; 8.74]	< 0.01	0.5272	0.7261	79%
Omitting Tan, L.−2019		7.03	[5.61; 8.80]	< 0.01	0.5392	0.7343	79%
Omitting Zhao, L−2022		7.06	[5.64; 8.85]	< 0.01	0.5423	0.7364	79%
**Random effects model**		**7.07**	**[5.66; 8.83]**	**< 0.01**	**0.5317**	**0.7292**	**79%**

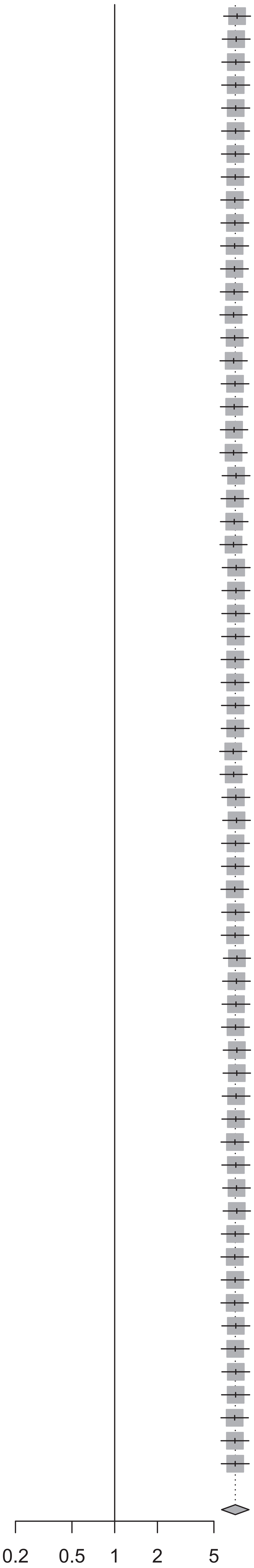

Figure S19 The results of sensitivity analysis of HR of recurrence (Multivariable analysis) of pan-cancer.
